# Patterns and Emerging Trends in Global Ocean Health

**DOI:** 10.1371/journal.pone.0117863

**Published:** 2015-03-16

**Authors:** Benjamin S. Halpern, Catherine Longo, Julia S. Stewart Lowndes, Benjamin D. Best, Melanie Frazier, Steven K. Katona, Kristin M. Kleisner, Andrew A. Rosenberg, Courtney Scarborough, Elizabeth R. Selig

**Affiliations:** 1 National Center for Ecological Analysis and Synthesis, Santa Barbara, California, United States of America; 2 Bren School of Environmental Science and Management, University of California Santa Barbara, Santa Barbara, California, United States of America; 3 Imperial College London, Silwood Park Campus, Ascot, United Kingdom; 4 Nicholas School of the Environment, Duke University, Durham, North Carolina, United States of America; 5 Betty and Gordon Moore Center for Science and Oceans, Conservation International, Arlington, Virginia, United States of America; 6 *Sea Around Us* Project, University of British Columbia, Vancouver, British Columbia, Canada; 7 Union of Concerned Scientists, Cambridge, Massachusetts, United States of America; Aristotle University of Thessaloniki, GREECE

## Abstract

International and regional policies aimed at managing ocean ecosystem health need quantitative and comprehensive indices to synthesize information from a variety of sources, consistently measure progress, and communicate with key constituencies and the public. Here we present the second annual global assessment of the Ocean Health Index, reporting current scores and annual changes since 2012, recalculated using updated methods and data based on the best available science, for 221 coastal countries and territories. The Index measures performance of ten societal goals for healthy oceans on a quantitative scale of increasing health from 0 to 100, and combines these scores into a single Index score, for each country and globally. The global Index score improved one point (from 67 to 68), while many country-level Index and goal scores had larger changes. Per-country Index scores ranged from 41–95 and, on average, improved by 0.06 points (range -8 to +12). Globally, average scores increased for individual goals by as much as 6.5 points (coastal economies) and decreased by as much as 1.2 points (natural products). Annual updates of the Index, even when not all input data have been updated, provide valuable information to scientists, policy makers, and resource managers because patterns and trends can emerge from the data that have been updated. Changes of even a few points indicate potential successes (when scores increase) that merit recognition, or concerns (when scores decrease) that may require mitigative action, with changes of more than 10–20 points representing large shifts that deserve greater attention. Goal scores showed remarkably little covariance across regions, indicating low redundancy in the Index, such that each goal delivers information about a different facet of ocean health. Together these scores provide a snapshot of global ocean health and suggest where countries have made progress and where a need for further improvement exists.

## Introduction

International, national and local-scale policies increasingly call for actions to improve ocean ecosystem health (e.g., [1–4]). For example, the European Union has set the overall objective of achieving ‘good environmental status’ for marine waters and has initiated broad ranging and binding directives to motivate and support Member States to pursue this goal [5]. Similar objectives have been set in the United States with the recent National Ocean Policy, which strives to achieve healthy oceans [6]. In all of these cases, quantitative measures – or indicators – of ecosystem health are the only way to gauge whether ecosystem health is improving, and thus whether management objectives are being achieved.

Indicators of ecosystem health are most useful if they are sufficiently comprehensive to provide information about the status of and potential interactions among all components of the ecosystem. Indicators that concentrate on a single component of an ecosystem are valuable for focused analyses, but cannot represent the ‘health’ of the system as a whole because they may miss important interactions among components. In addition, since people are the end-users, ecosystem health indicators are most useful if they focus on the full suite of components that people value and can influence through their actions. The Ocean Health Index addresses these needs by describing and measuring the health of ocean systems through ten widely shared goals or values pertaining to how people interact with and benefit from marine ecosystems (Table 1; [7]). As such, we define a healthy ocean through the lens of coupled socio-ecological systems as ‘one that sustainably delivers a range of benefits to people now and in the future’ [7]. An important consequence of tracking different factors together is that one can develop an understanding of potential trade-offs or synergies between components, thus making informed choices on how to improve health.

**Table 1:** The ten goals and their component sub-goals that comprise the Ocean Health Index, along with a brief description of the benefit measured by the goal.

Goal/sub-goal	Benefit measured
Food provision	Sustainable food production
Fisheries, wild capture	Sustainably harvested wild-capture seafood
Mariculture	Sustainability and productivity of mariculture
Artisanal fishing opportunities	Availability of fishing opportunities to those who need them
Biodiversity	Conservation of species and habitats for their existence value
Species	Conservation of species for their existence value
Habitat	Conservation of habitats for their existence value
Clean waters	Clean ocean waters free of pollution
Sense of place	Conservation of relevant places and species for their cultural value
Lasting special places	Conservation of relevant places for their cultural value
Iconic species	Conservation of species for their cultural value
Coastal livelihoods & economies	Employment (livelihoods) and revenues (economies) from marine sector
Livelihoods	Employment from marine sector
Economies	Revenues from marine sector
Tourism & recreation	Number of tourists and quality of their experience
Coastal protection	Conservation of key protective habitats
Carbon storage	Conservation of key carbon storing habitats
Natural products	Amount of sustainably harvested non-food products

Further details are provided in the Supplementary Methods in S1 File.

Ecosystem health indicators best track progress if measured repeatedly and consistently over time. Doing so can not only reveal current condition and direction of change, but also help track how decline or improvement is linked with past actions and identify where future actions are most needed to mitigate declines. Even when not all component data layers have been updated since the previous assessment, an new assessment adds value because it can 1) incorporate any newly-developed methods, 2) include and evaluate the impact of newly-available data, and 3) improve precision for scores with component data that have been revised (for example, when governments revise past economic growth and labor market statistics). Here, we repeated and improved upon the initial global assessment of ocean health [7], demonstrating the utility of repeated annual assessments and hopefully catalyzing efforts to continue such assessments long into the future.

A key challenge for any indicator is to remain flexible and adaptive to new and improved data and scientific understanding so the ‘best available science’ is incorporated as it becomes accessible while still permitting temporal comparability. In fact, an effective indicator will promote the collection of new data and stimulate the development and refinement of assessment methods. As new data and models become available, ecosystem health indicators should incorporate them. However, any changes in methods and data sources made between assessments make it difficult to determine whether changes in scores through time are due to changes in methodology or actual changes in the system being assessed, which is the only measure used by managers, policy makers and scientists. As such, it is important to recalculate previous assessments with updated models and data so that differences between assessments are due to change in ecosystem condition rather than updated data or methodology.

This second annual assessment of the Ocean Health Index incorporates fifty new reporting regions (countries and territories) previously aggregated due to data limitations (Table A in S1 File), improved models for several goals designed to better capture goal objectives and leverage improved data, and new and updated data for more than half of all data used in the Index. These improvements incorporate the best available science, but also include methodological changes that could confound temporal comparisons. To allow such comparisons, we recalculated Index scores for the previous assessment using the new methods. However, in a few cases, such as when a new data source becomes available, such hindsight calculations are not possible. Fortunately, methods for composite indices tend to stabilize over time (e.g., GDP, WGI; [8–9]), allowing for increasingly comparable assessments.

We also performed analyses to better understand the relationship among goals, and to compare how the Index scores relate to other descriptive and commonly assessed measures of countries (Human Development Index, Gross Domestic Product, and population size). Although limited to just two years, the assessments of spatial patterns of change in ocean health presented here offer valuable insight into where and why ocean conditions, and the human communities that depend on them, are changing.

## Methods

We provide here a brief overview of the methods for calculating the Index. Extensive details are provided in the Supplementary Methods in S1 File, Table H in S1 File, and [7, 10]. Kleisner *et al*. [11] and Selig *et al*. [12] provide detailed explorations of methods and results for previous assessments of the food provision and biodiversity goals, respectively. Here we focus primarily on describing changes and updates that were developed and applied for this second annual global assessment. We refer to this current assessment as the 2013 assessment and the previous year as the 2012 assessment to reflect when the assessments were completed; these assessments are based on the most current data available at that time. Actual data used in each assessment span a number of recent years (Table I in S1 File), representing the most recent available information for each data layer.

An overall Index score for each country or region, *I*, is calculated as the weighted sum of the scores for each goal assessed, *G*, in the Index [7], such that:

$$I=\sum_{i=1}^{N}\alpha_{i}G_{i},$$ (Eq. 1)

where α is the importance (i.e. weight) placed on each goal *i* and is initially assumed to be equal for all *N* goals, as was done previously [7]. Goal scores are calculated as the average of current (*x*_*i*_) and likely future status (${\hat{x}}_{i,F}$), with current status measured as present value (*X*_*i*_) relative to a reference value (*X*_*i,R*_), such that *x*_*i*_ = *X*_*i*_ / *X*_*i,R*_, and likely future status measured as current status modified by the recent trend (*T*), cumulative pressures (*p*), and resilience (*r*), such that:

$${\hat{x}}_{i,F}={\left(1+\delta \right)}^{-1}\left[1+\beta T_{i}+(1-\beta )(r_{i}-p_{i})\right]x_{i},$$ (Eq. 2)

where δ is the discount rate (δ = 0) and β is the relative importance of trend versus the difference between pressures and resilience in determining the likely future status (β = 0.67). We used a discount rate of 0% when calculating overall Index scores because discount rates tend to be smaller when assessing something with intrinsic, aesthetic, or spiritual value, regardless of the time horizon, as these values tend to be time-independent. Many of the goals have strong non-market values associated with them. Sensitivity analyses for past global assessments showed scores to be robust to higher discount rates [7]. β gives the trend twice as much importance as pressures and resilience in determining the likely future status. We define reference points as the maximum sustainable level of production of each goal, which is generally equivalent to a management goal or ‘target’ [10, 13]. However, in some cases management may choose a target lower than these reference points for practical or socio-political reasons. Tables J and K in S1 File provide details on how pressure and resilience data are applied to each goal.

The first global assessment [7] focused on Exclusive Economic Zone (EEZ)-level results, but aggregated information from smaller areas and territorial holdings into larger reporting units in cases where available data were particularly sparse. For the 2013 assessment, we report most of these regions separately (Table A in S1 File), but doing so required a variety of procedures to fill data gaps so that these additional, often data-limited, locations could receive scores (see section 6, Supplementary Methods in S1 File, for full details). Both approaches make assumptions about how well data represent reality across different scales, but reporting more regions preserves higher resolution data when they are available and allows each region to receive its own score, albeit in some cases with potentially less certainty. Because these new regions tend to be data limited, it is not possible to know whether this new method increased or decreased uncertainty. A full list of reporting regions used in the current assessment is provided in Table G is S1 File.

### Fisheries sub-goal

We changed the methods for calculating the status of the fisheries sub-goal of the food provision goal in response to improved models and available data, and both informal and formal suggestions [14–15]. The indicator aims to assess the amount of wild-caught seafood that can be sustainably harvested, with sustainability defined by multi-species yield, and with penalties assigned for both over- and under-harvesting. This approach requires establishing a reference point at which harvest is maximized within sustainable bounds. Previously this reference point was derived from an estimate of multi-species maximum sustainable yield (*mMSY*; [7, 11]), modified from Srinivasan et al. [16] to provide stock status assessments using catch data only (i.e., a ‘data-limited’ approach where variables generally required by formal stock assessment methods are unknown, as is the case for most commercially exploited species across the majority of areas reported to the Food and Agriculture Organization; FAO). In the current assessment, we modified our approach in multiple ways.

First, we changed the model used to assess the status of each individual stock. Since the initial assessment [7], several new data-limited approaches have been developed to assess fisheries that leverage globally-available catch data [17–21]. Building on these methodological advances, we developed a new approach to assessing food provision from wild caught fisheries that is based on estimating population biomass relative to the biomass that can deliver maximum sustainable yield (*B/B*_*MSY*_) for each landed stock. Estimates of *B/B*_*MSY*_ were obtained by applying a model developed by Martell & Frœse [19], modified according to methods described in Rosenberg et al. [21], and hereafter referred to as the “catch-MSY” method. This method was chosen over other data-limited methods because simulation-tests demonstrated that it performed well in predicting stock status for simulated stocks having a broad range of life history traits and different known sources of uncertainty, i.e., environmental stochasticity, time-series length, initial depletion, and temporal autocorrelation [21]. The original catch-MSY method [19] is derived from stock reduction analysis [22], whereby a time series of catch is combined with an estimate of the final biomass relative to an unfished or initial biomass state (i.e., depletion level) in order to estimate historical biomass trends. A Schaefer surplus production model is used to produce ‘viable’ combinations of the intrinsic rate of growth, *r*, and the carrying capacity, *K*. ‘Viable’ was defined as any pair of *r* and *K* that did not allow the stock biomass to collapse or to exceed carrying capacity. In the original formulation of Martell & Froese [19], the geometric mean values of *r* and *K* were used to derive an estimate of *MSY*. Rosenberg et al. [21] modified this method by producing a biomass time series for each of the viable *r-K* pairs using the surplus production model. The arithmetic mean biomass time series was selected and the current year stock abundance (*B*) relative to the abundance that achieves MSY (*B*_*MSY*_) produced a measure, *B/B*_*MSY*_.

The model applies a constraint on the prior distribution used by the Bayesian model to predict final biomass (i.e., 0.01–0.4) based on whether the ratio of catch in the final year relative to historical peak catch is less than 0.5. When the ratio is greater than 0.5, the ratio is set 0.3–0.7. In applying this approach to the global catch data, we found this prior distribution caused the model to frequently estimate a decline in B/B_MSY_ for stocks with declining catch in the final years of the time-series. Explorations suggested that these included cases of managed fisheries where reduced catch was likely due to declining effort rather than declining population biomass. As an alternative, we applied a uniform prior, thereby removing this constraint. This resulted in estimates of biomass that were increasing when catch in the final years was declining (i.e., biomass increases due to a reduction in fishing pressure). This is an unlikely outcome in poorly regulated fisheries. Therefore, for analyses here we applied these two formulations of the prior distribution discriminately based on the level of management of a given stock in a given region; we assumed that the original constrained prior on final biomass is more appropriate in poorly regulated fisheries, while places with stronger fisheries management regulations were best modeled using a uniform prior on final biomass. In order to discriminate between these two cases, we assigned a governance-based resilience score to each stock, S_r_. The resilience score was a mean of the fisheries resilience score (see S1 File) across all regions where the stock was caught, weighted by the relative mean catch in each of the regions:

$$S_{r}=\sum_{z=1}^{n}r_{z}*\frac{c_{z}}{\sum c_{j}}$$ (Eq. 3)

where n is the number of regions z (EEZs or high seas) in which the stock is caught, r_z_ is the fisheries resilience score assigned to that region, c_z_ is the mean catch of that stock in that region through time, and c_j_ is the mean catch of each of that stock in each of the regions. We estimated B/B_MSY_ with a uniform prior for all stocks with a resilience score of 0.6 or above, and used the model with the original constrained prior for all stocks with resilience scores less than 0.6. We recognize there is no precedent for using the model this way, and further testing would be valuable to establish more rigorous rules for how the priors are defined. Nevertheless, based on current knowledge and understanding, this approach was the best option. The estimated *B/B*_*MSY*_ was then used to produce stock status scores, *SS*, for each individual stock.

The catch-MSY approach improves upon the method used in Halpern et al. [7] by using a less simplistic relationship between catch and stock status. It is based on a mechanistic understanding of the connection between harvest dynamics and population dynamics and uses this functional link to infer stock depletion levels (see also [20]). Simply put, it takes into account life history traits and observed relationships between catch trajectories and depletion level, rather than just using an empirically-observed relationship between peak catch and MSY. Because the model uses more information from the catch time-series, it may more accurately describe stock status. In the case of developing fisheries that have not yet exploited stocks to their full productive potential (i.e., with catch levels that steadily increase over time), both approaches are flawed. The previous approach assigned a perfect score to these stocks. The new method does not resolve this issue fully, but it can be more informative at least in some cases where the time series is not monotonically increasing.

Second, we modified the approach to modeling fisheries to capture the portfolio effect afforded by preserving catch diversity [23], the status of wild caught fisheries (*x*_*FIS*_) was calculated as the geometric mean of the stock status scores. The geometric mean allows stocks that are doing poorly, in particular smaller ones, to have a stronger influence on the overall score than they would using an arithmetic mean, even though their catch, *C*, may contribute relatively little to the overall tonnage of harvested seafood. The behavior of the geometric mean is such that improving a well-performing stock is not rewarded as much as improving one that is doing poorly. We believe this response is desirable because the recovery of stocks in poor condition requires more management effort and can have more important effects on the system than increasing the abundance of a species that is already abundant. Use of the geometric mean operationalizes our view that a healthy ocean sustainably provides a range of benefits to people now and in the future because it values both absolute tons of fish produced, the distribution of that catch among species, and the condition (*B/B*_*MSY*_) of all harvested species in the system. Thus, it gives credit for preserving the health of the full range of species. The stock status scores for each taxon landed within each FAO major fishing area (*A*, noted below) were then combined weighting each taxon by its relative contribution to overall catch (*C*) within each reporting region and year, such that:

$$x_{FIS}=\prod_{i=1}^{n}SS_{i}{}^{{}^{\left(\frac{C_{i}}{ƩC_{i}}\right)}}$$ (Eq. 4)

where *i* is an individual taxon and *n* is the total number of taxa in the reported catch for that country or region throughout the time-series, and *C* was calculated as the taxon catch averaged across the time series from the first non-null record within each of our reporting regions.

Because many fish populations straddle the boundaries of EEZs, we calculated *B/B*_*MSY*_ by applying the catch-MSY model to catch aggregated within each major FAO fishing area, *A*. These values of stock status were then assigned to our reporting regions. Goal status was calculated at the spatial scale of our reporting regions, with each taxon’s stock status weighted by its mean catch relative to each reporting region’s total catch (note that for a geometric mean, weights appear in the exponent). This differs from the previous iteration, where the catch stream from each EEZ (based on catches that were spatially allocated to EEZs by the *Sea Around Us* project) was analyzed separately and aggregated up to the previous Ocean Health Index reporting regions. Any aggregation method will be biased in some way, but populations with the largest catches are most often straddling stocks, so they are not likely to be subject to aggregation biases. Instead, erroneous aggregation of catch could occur more often with high patchy species that primarily include small, sedentary populations that contribute little to a country or region’s fisheries and thus have little influence on overall catch.

The taxonomic level of each reported taxon was assigned to 1 of 6 categories derived from its ISSCAAP code (FAO International Standard Statistical Classification of Aquatic Animals and Plants, http://www.fao.org/fishery/collection/asfis/en, see ‘S5.68. Spatially-allocated catch data’ in S1 File for more details). *B/B*_*MSY*_ values could be directly calculated only when catch was reported at the species level, i.e., taxon group level 6, as the time-series of catch across miscellaneous taxa is unlikely to fit required model assumptions (the method of deriving stock status scores for higher level taxa is described below). Overall, we were able to evaluate a total of 1874 stocks. The estimated species level values of *B/B*_*MSY*_ were used to derive a stock status score, *SS*, such that the best score is achieved for stocks at *B/B*_*MSY*_ = 1, with a 5% error buffer, and the score decreases as the distance of *B* from *B*_*MSY*_ increases, due to under- or over-exploitation. For each species reported, within each major fishing area *A, SS* was calculated as:

$${\text{SS}}_{A,g=6}=\{\begin{array}{lll} {\text{B/B}}_{MSY} & if & {\text{B/B}}_{MSY}<0.95\\ 1 & if & 0.95\leq {\text{B/B}}_{MSY}\leq 1.05\\ max\left\{1-\alpha \left({\text{B/B}}_{MSY}-1.05\right),\beta \right\} & if & {\text{B/B}}_{MSY}>1.05 \end{array}$$ (Eq. 5)

where, for *B/B*_*MSY*_ < 1 (-5% buffer), *SS* declines with direct proportionality to the decline of *B* with respect to *B*_*MSY*_, while for *B/B*_*MSY*_ > 1 (+5% buffer), *SS* declines at a rate α, where α = 0.5, so that as the distance of *B* from *B*_*MSY*_ increases, *SS* is penalized by half of that distance. For *B/B*_*MSY*_ > 1 (+5% buffer), β is the minimum score a stock can get, and was set at β = 0.25. The α value ensures that the penalty for under-harvested stocks is half of that for over-harvested stocks (α = 1.0 would assign equal penalty). The β value ensures stocks with *B/B*_*MSY*_ > 1.4 due to, for example, an exceptionally productive year, are not unduly penalized, and also recognizes that goal scores are more easily improved when stocks are under-harvested (i.e., by increasing fishing pressure) than when they are over-harvested and need to be rebuilt. Both parameters α and β were chosen arbitrarily because there is no established convention for this particular approach. Thus, consistent with previous work [7], countries or regions are rewarded for having wild stocks at the biomass that can sustainably deliver the maximum sustainable yield, +/-5% to allow for measurement error, and are penalized for both over- or under-harvesting.

Third, the method of penalization for poorly reported catch statistics was modified. In the previous version [7], we used a taxonomic reporting index, *T*_*C*_, to incorporate the consequence of under-reporting catch. This indicator was based on the assumption that if a country (or region) reported catching a species, and this species’ area of distribution overlapped with the EEZ of another country, then the other country must also be fishing it even if they do not report it as part of their catch. This *T*_*C*_ variable was intended to capture the proportion of a country’s catch that was not being officially reported and hence not monitored or managed, but it was subject to underestimation in countries with very large coastlines or remote locations, and over-penalizing places with many neighboring countries. In the calculations performed in 2012, this parameter strongly penalized scores in most countries [11]. Here, for taxa reported at a lower resolution than species, we developed a method to estimate stock status that accounts for coarser resolution data. The species-based estimates within the same fishing area and year were used to generate missing scores. An increasing penalty was applied for increasingly coarser taxonomic reporting, as this is considered a sign of minimal monitoring and management, so that, for a given taxonomic aggregation *g* (i.e., increasing coarseness of taxonomic reporting, when *g*<6), a proxy value for *B/B*_*MSY*_ was estimated as follows:

$${\text{B/B}}_{\text{MSY}}{}_{A,g<6}=\{\begin{array}{lll} 0.01*\text{median}\left\{{\text{B/B}}_{\text{MSY}}{}_{A,g,\forall g=6}\right\} & if & g=1\\ 0.25*\text{median}\left\{{\text{B/B}}_{\text{MSY}}{}_{A,g,\forall g=6}\right\} & if & g=2\\ 0.50*\text{median}\left\{{\text{B/B}}_{\text{MSY}}{}_{A,g,\;\forall g=6}\right\} & if & g=3\\ 0.80*\text{median}\left\{{\text{B/B}}_{\text{MSY}}{}_{A,g,\;\forall g=6}\right\} & if & g=4\\ 0.90*\text{median}\left\{{\text{B/B}}_{\text{MSY}}{}_{A,g,\;\forall g=6}\right\} & if & g=5 \end{array}$$ (Eq. 6)

The resulting value was then used to obtain the stock status score as shown in Equation 4. Thus, this new model adopts a more accurate estimate of individual stock status, accounts for multi-species effects by aggregating stock status scores through a geometric mean, and applies penalties for coarse taxonomic reporting as a reflection of weak monitoring effort. However, the philosophical approach first introduced by Halpern et al. [7] is maintained in its essence through two key properties: individual stock status scores are penalized for both over- and under-exploitation, and all species reported are assessed, albeit through a data-limited approach. Further details on this sub-goal model are provided in the Supplementary Methods in S1 File.

### Mariculture Sub-goal

We also improved the method for calculating the status and reference point for the mariculture sub-goal of the food provision goal, based on sensitivity analyses exploring how scores were affected by choice of reference point [11]. The new approach calculates a country or region’s mariculture score based on total yield from mariculture (*Y*_*M*_), the species-specific sustainability of that harvest (*S*_*M,k*_), and coastal population density (*P*_*C*_). Yield (and its sustainability) is adjusted by coastal population instead of coastal area (as was done previously in [7]) under the assumption that locally available workforce, coastal access and infrastructure needed for mariculture were proportional to that density. The current status of mariculture (*x*_*MAR*_) is thus:

$$x_{MAR}=\frac{Y_{M}}{Y_{ref}},$$ (Eq. 7)

where:

$$Y_{M}=\frac{\sum_{1}^{k}Y_{k}S_{M,k}}{P_{C}},$$ (Eq. 8)

and the reference point (*Y*_*ref*_) is:

$$Y_{ref}=P_{95}\left(Max\left\{Y_{M}\right\}\right),$$ (Eq. 9)

with the 95^th^ percentile value (P_95_) used due to the high skew in data.

### Tourism & Recreation Goal

The tourism & recreation goal aims to capture the number of people, and the quality of their experience, visiting coastal and marine areas and attractions. The economic benefits of coastal tourism industries (i.e., jobs, wages, revenue), which can be important to coastal economies, are assessed separately as part of the coastal livelihoods & economies goal. Few non-economic indicators of tourism and recreation exist at the global scale, and thus the original approach in the 2012 assessment approximated this goal by measuring the number of international tourists arriving by airplane to coastal countries or regions, adjusting these values to the region’s population density to allow comparability across regions, and accounting for their average length of stay. This approach was sub-optimal, in part, because it did not account for domestic tourism, which is a large part of tourism in many countries, especially large countries such as Brazil, Canada, Russia, Australia and the USA. In the 2013 assessment we develop a different model to capture tourism and recreation, one that better accounts for both international and domestic tourism.

We used employment in the tourism sector as a reasonable proxy measure for the total number of people engaged in coastal tourism and recreation activities. Employment within this sector should respond dynamically to the number of people participating in tourist activities, based on the assumption that the number of hotel employees, travel agents and employees of other affiliated professions will increase or decrease with changing tourism demand within different regions.

Ideally there would be data available specifically for employment in coastal tourism industries, however the best data available at a global scale report total number of jobs, not just coastal jobs, within the travel and tourism industries (World Travel and Tourism Council (WTTC)). These data include jobs for both leisure and business that are directly connected to the tourism industry, including accommodation services, food and beverage services, retail trade, transportation services, and cultural, sports and recreational services, but exclude investment industries and suppliers [24]. Unfortunately it was not possible to determine the proportion of jobs affiliated with strictly leisure tourism. However, some (unknown) proportion of business travelers also enjoy the coast for leisure during their visit to coastal areas, such that we assumed all travel and tourism employment was related to tourism and recreation values. Regional assessments of the Index can make use of better-resolved data and more direct measures of how people enjoy and recreate in coastal areas, as has been done within the US West Coast [13], where data for participation in 19 different coastal recreational activities were available.

To approximate coastal travel and tourism employment using WTTC data, we calculated the proportion of direct employment in the tourism industry (*E*_*d*_) relative to total labor force (*E*_*t*_). As in 2012, we used the travel and tourism competitiveness index (TTCI) from the World Economic Forum [25] to capture the sustainability (*S*_*t*_) of the tourism industry.

Therefore, the status of tourism & recreation (*x*_*TR*_) is:

$$x_{TR}=E_{d}\cdot S_{t},$$ (Eq. 10)

where *E*_*d*_ is defined as the proportion of employees directly involved in the travel and tourism industry (*E*_*t*_) relative to the total employees in that country (or region), calculated as the country’s total labor force (*L*_*t*_) corrected by the percent of the population that is unemployed (*U*_*t*_), such that:

$$E_{d}=\frac{E_{t}}{L_{t}-\left(L_{t}\times U_{t}\right)}.$$ (Eq. 11)

Because we do not know how employment patterns vary geographically within sectors for each country, we assume that the proportion employed in the tourism industry is the same in coastal areas as it is away from the coast, and thus *E*_*i*_ is the same whether applied solely to coastal areas or to the entire country. As such, the status of this goal could be increased by increasing a) the number people employed in the tourist industry relative to unemployment-corrected changes in the labor force within the whole country or b) the sustainability of tourism and recreation (measured with the TTCI).

Data for *E*_*t*_ existed for 148 regions (i.e., data were missing for 63 reporting regions; see Fig. C in S1 File). To fill the gaps for missing regions we used final goal scores rather than *E*_*t*_ values for the 148 regions and then followed the gap-filling guidelines described in section 6 of S1 File. We avoided gap-filling the *E*_*t*_ data layer because doing so created cases where the number of tourism jobs exceeded the reported labor force (data from the World Bank).

Given perfect data, one would use the best score across all regions and all years as the reference point (Saba in 2008). However, the highest-scoring regions were outliers in the distribution of scores (the second best performing region was 53% of the maximum and the third was 18%) and most scores were clustered around zero; we therefore rescaled all scores to the 90^th^ percentile score (the 21^st^ ranked region, Belize, with a score of 3). All regions above this score received a current status score of 100.

### Data and Additional Analyses

Table I in S1 File lists all data layers, indicating which of the 77 layers were a) new data (8% of data layers used), b) updates of data sources used previously [7] but now containing new year(s) of data (47%), c) used previously but not updated because no new values have been reported (42%), and d) used previously but no longer included (4%). Details on all these data are provided in S1 File. Changes in category b include several data layers that were revised retrospectively for previous years, similar to how countries adjust economic data for past quarters and years. Such revisions were most notable for data from the Food and Agriculture Organization (FAO), used in the fisheries, mariculture and natural products goals. Additionally, Fig. C in S1 File provides a summary of which goals for each region required some level of spatial gap-filling due to non-reporting, with results summarized as the proportion of layers per goal, per region, that required gap-filling.

To assess how Index scores compared to other measures of the health of a region, we applied a regression model to determine whether the variation in OHI scores varied with national gross domestic product from 2012 (GDP data, USD; [9]), population data from 2011 [9], and Human Development Index (HDI) scores from 2012 (HDI, [26]). GDP and population variables were natural log- transformed prior to analysis. We compared models with every combination of these variables and selected the best model based on the Bayesian Information Criterion (BIC). To assess the relationship between goal scores, we determined the Pearson correlation coefficients between each pair of goals for all 221 reporting regions and fit scatterplots of each pair using locally-weighted polynomial regression (the lowess function in R).

## Results

**Fig 1:**
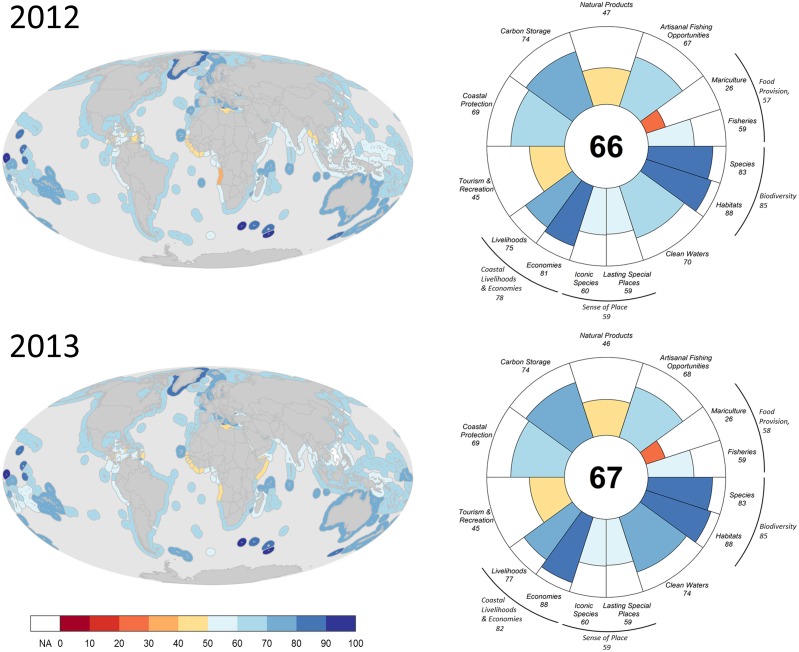
Global scores for the Ocean Health Index. Maps of overall Index scores for each reporting region (left panels) and global Index and goal scores (right panels) for newly calculated 2013 values (bottom panels) and recalculated 2012 values using updated methods and data (top panels). Colors in all cases indicate score values.

**Fig 2:**
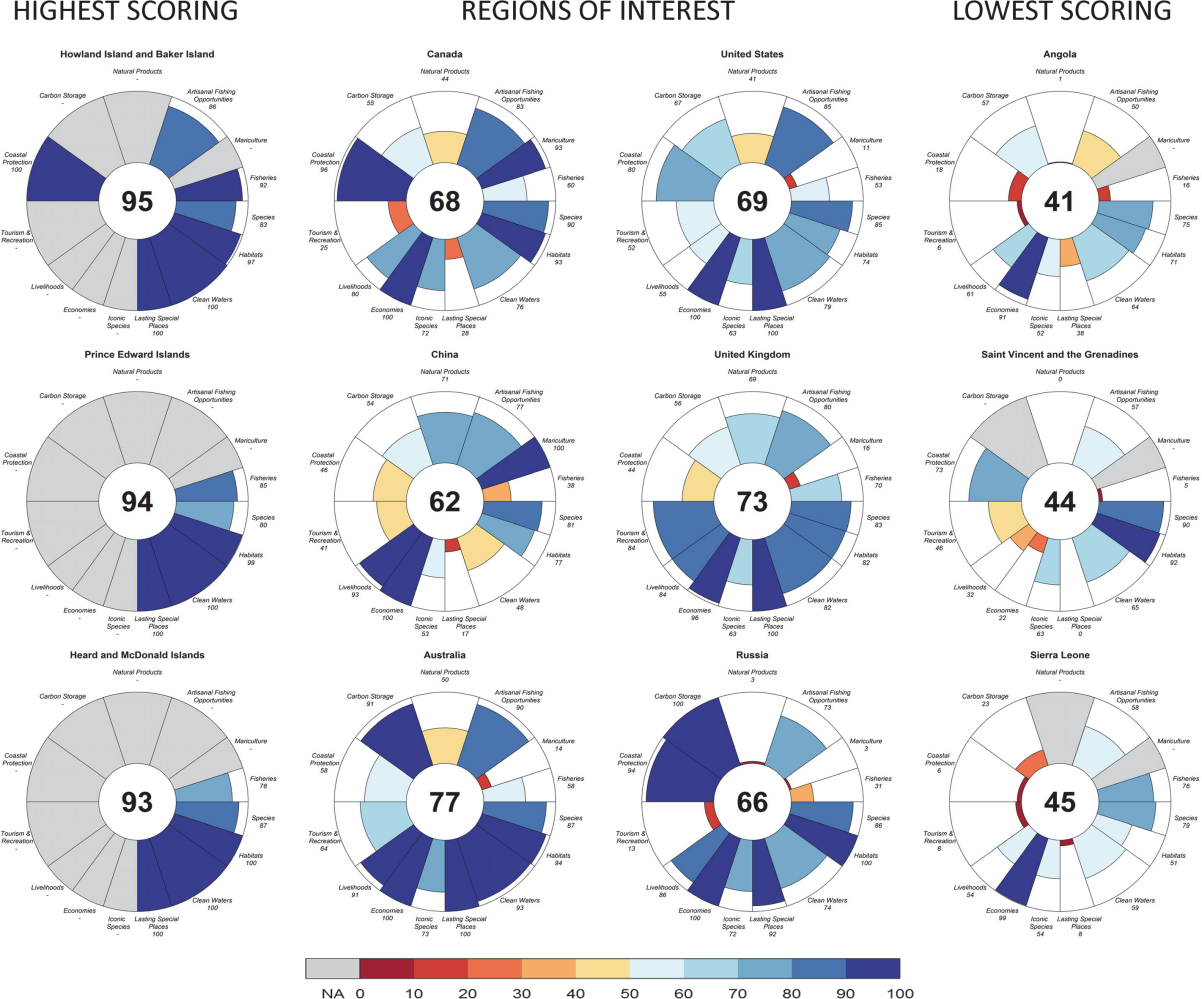
Index and goal scores for representative countries and territories for 2013. Examples shown include three of the top five highest-scoring regions in the world, the three lowest scoring regions, and regions of potential interest.

Scores by country or region in 2013 ranged from 41 to 95, and the global Index score was 68 (Fig. 1). The ten highest scores were all obtained by island territories or nations, including Howland Island and Baker Island (95), Prince Edward Islands (94), Heard and McDonald Islands (93), Macquarie Island (87), Kerguelen Islands (87), Jarvis Island (87), Crozet Islands (86), Greenland (83), Johnston Atoll (82), and Malta (81) (see Fig. 2; Table L in S1 File). Five of these regions are in the southern hemisphere, either uninhabited or with small populations, and three of the others are tropical island nations or territories.

Heard and McDonald Islands’ score (93) was the highest for any populated region, though the population is only 110. Malta’s score (81) was the highest for a nation with a larger population (nearly 450,000). Top scores for progressively larger populations were Greenland (83) for a region with more than 10,000, New Zealand (79) for more than one million, Australia (77) for more than 10 million, and Germany (74) for more than 50 million people. The 10 lowest scoring countries or regions (in descending order) were Guinea Bissau (48), East Timor (48), Liberia (47), Sierra Leone (45), Haiti (45), Nicaragua (45), Ivory Coast (45), Democratic Republic of the Congo (45), Saint Vincent and the Grenadines (44), and Angola (41) (see Fig. 2; Table L in S1 File).

**Fig 3:**
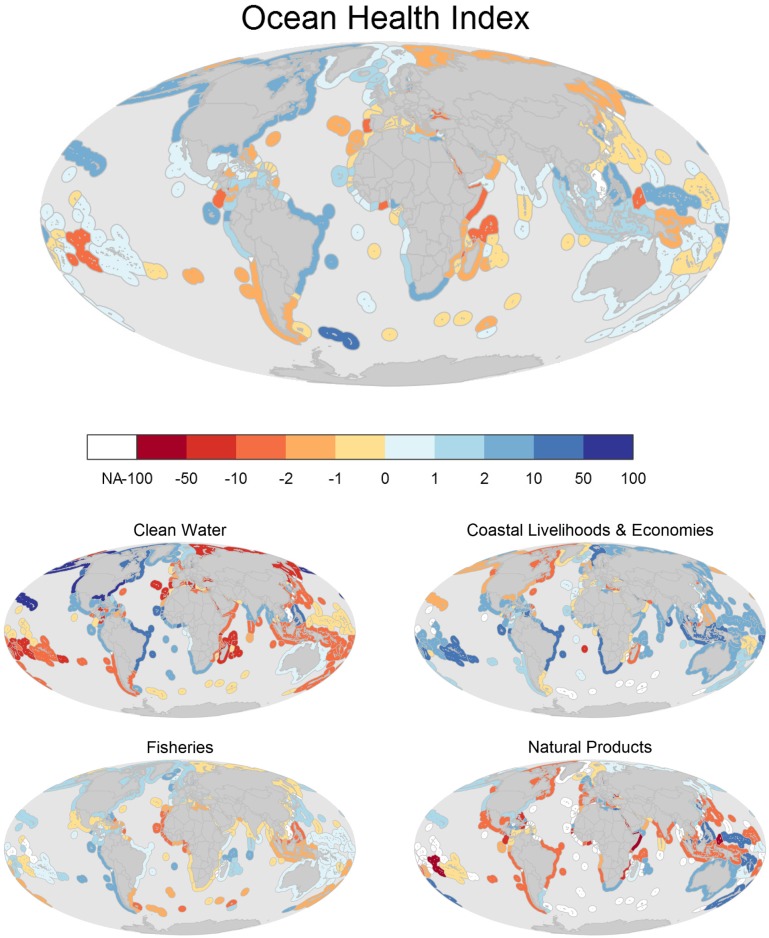
Change in Index and goal scores. Maps of the absolute difference of 2013 scores minus 2012 scores for each region for the overall Index (top panel) and four goals and sub-goals (bottom panels). Maps for all goals and sub-goals are presented in Figs. A-B in S1 File.

Index scores for most countries (or regions) changed relatively little compared to recalculated scores from the 2012 assessment, with only nine countries changing scores by 5 or more points (mean ± SD = 0.06 ± 2.22 points; Figs. 1 and 3, Table M in S1 File). The score for South Georgia and South Sandwich Islands (77) showed the greatest year-to-year increase for any region (+12 points, or 16%) due primarily to designation last year of 1 million square kilometers of its highly productive waters as a marine protected area, which more than doubled its score for the sense of place goal. Cook Islands (56) saw the largest annual decrease (-8 points, or -14%), primarily because of large decreases in its natural products goal (-64 points) due to large decreases in harvest of ornamental fishes and shells. Table 2 shows the regions with the greatest positive and negative changes in scores from 2012 to 2013, with the full list of regions in Table M in S1 File.

**Table 2:** Regions with the greatest positive (>+5 points) or negative (>-5 points) change in overall Index scores from 2012 to 2013, with changes in goal scores also shown (see Table M in S1 File for full results).

Country/EEZ	Index	Food Provision	Artisanal Fishing Opportunities	Natural Products	Carbon Storage	Coastal Protection	Coastal Livelihoods & Economies	Tourism & Recreation	Sense of Place	Clean Water	Biodiversity
**Greatest increases**											
South Georgia and the South Sandwich Islands	12	-1.8	NA	NA	NA	NA	NA	NA	47.6	0	1.5
Bulgaria	9.3	33	0.2	44	NA	NA	-0.1	-0.1	-0.1	-2.3	-0.1
United States	8.4	1.3	1.3	1.3	0.1	1.8	-1.3	0.6	0.2	79	0.5
Saint Pierre and Miquelon	7.4	2.3	1.0	NA	NA	NA	0.1	0.6	0.1	48	0.2
Myanmar	7.1	-0.1	0.9	NA	0.3	0.1	8.9	0	0.2	8.5	0.2
Nigeria	5.0	-2.8	0.2	NA	0	0	6.0	0	0.1	2.6	-0.1
**Greatest decreases**											
Eritrea	-5.0	-1.0	0.1	-37.1	0	-0.1	0	-0.1	-0.1	-11.6	-0.1
Somalia	-7.2	0.2	0.2	-64.3	0.1	-0.1	2.1	0	0	-9.8	-0.1
Cook Islands	-7.7	-0.6	-0.7	-63.7	-0.6	-1.2	6.0	-0.8	-0.5	-13.2	-1.2

NA indicates the goal was not relevant to the region and so not given a score. Values outside the range of -10 to +10 are rounded to the nearest whole number.

Goal scores changed very little globally, with only one goal changing more than one point: natural products (-1.2 points; Table M in S1 File). Specific countries and regions showed much larger per-goal changes, with the largest positive changes for the lasting special places sub-goal in South Georgia Islands (+95 points) due to the addition of a very large MPA, the coastal economies sub-goal in Tuvalu (+71 points) due to a nearly 100-fold increase in revenue from aquarium trade (ornamental) fishing, and the clean waters goal in the United States (+79 points) due to a sharp drop in marine debris pollution. The largest negative changes were in the natural products goal in Somalia and Cook Islands (both -64 points) due to very large decreases in shell harvest in both cases and also large declines in ornamental fish harvest in Cook Islands, and the clean waters goal in Croatia (-27 points) and Turkey (-23 points) due to increases in marine debris pollution (see Table M in S1 File for a full list of changes, Figs. A and B in S1 File).

Improved methodologies for the fisheries and mariculture sub-goals and the tourism & recreation goal led to higher scores for recalculated 2012 scores (Fig. 1) compared to originally calculated scores in the 2012 assessment [7]. Globally, the fisheries sub-goal was 34 points higher, mariculture 16 points higher, and tourism & recreation 35 points higher with recalculated 2012 scores. These changes in scores occurred as a result of revised past data (e.g., FAO revised previous harvest data for natural products and food provision goals), improved methods for modeling the goals (in particular for setting sustainable reference points), and improved gap-filling procedures (see S1 File). All other goals and sub-goals changed scores in the recalculated 2012 assessment due primarily to better data gap-filling methods. Natural products improved 7 points due to more robust data processing techniques. Increased numbers of assessed species led to a 4 point increase in the species sub-goal but a 10 point decrease in the iconic species sub-goal. Changes in carbon storage (decreased 1 point), coastal protection (decreased 4 points), lasting special places (increased 18 points), and artisanal fishing opportunities (decreased 20 points) occurred due to the ability to assess mangrove habitat extent more precisely (both habitat-based goals), inclusion of recent data that were previously unavailable on protected areas (lasting special places), and updated GDP data (artisanal fishing opportunities). Coastal economies increased 14 points due to exclusion of a previously-used adjustment factor, revised economic data, and improved gap-filling methods, and coastal livelihoods decreased 9 points because of increased number of reporting regions.

**Fig 4:**
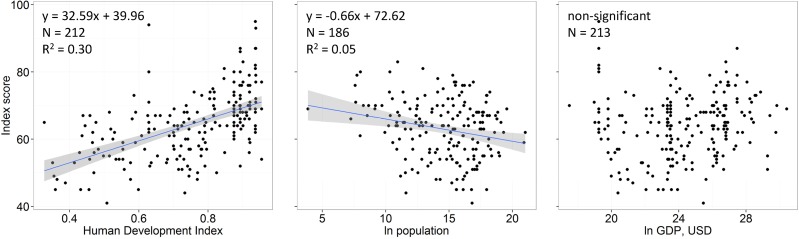
Relationship between Ocean Health Index scores and potential predictor variables. Scores per region are compared to A) human development index (HDI) scores, B) country-level population (natural log-transformed), and C) country-level gross domestic product (GDP, natural log-transformed).

**Fig 5:**
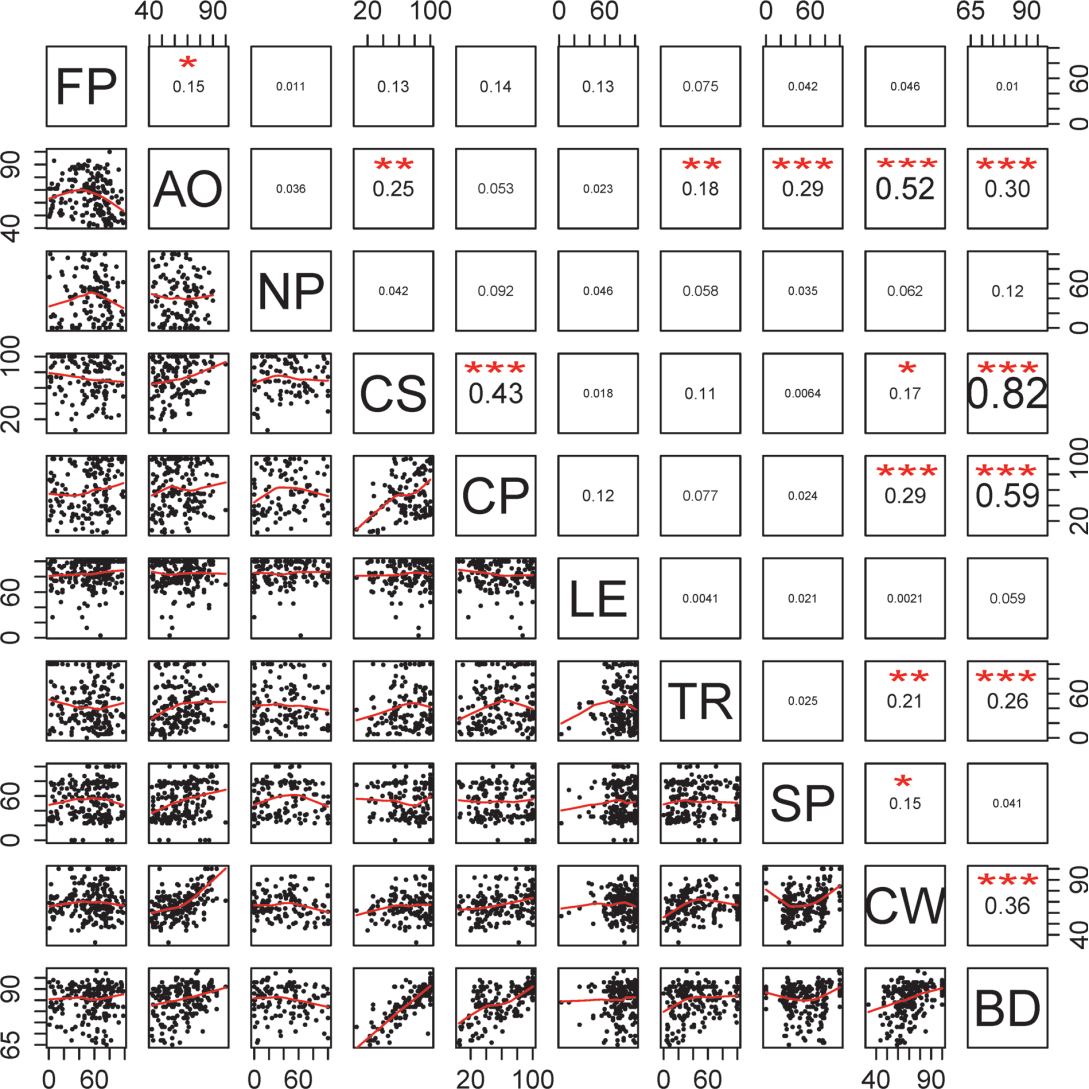
Correlation matrix of per-country pairwise goal scores. Two-letter codes in the diagonal are goal labels (FP = food provision, AO = artisanal fishing opportunity, NP = natural products, CS = carbon storage, CP = coastal protection, LE = coastal livelihoods and economies, TR = tourism and recreation, SP = sense of place, CW = clean water, and BD = biodiversity). Values in the upper right are the correlation coefficients for each comparison, with larger font size indicating stronger significance of the result. Plots in lower left are scatterplots of the data with locally-weighted polynomial regression (LOWESS) fits shown in red.

Index scores tended to be higher in countries or regions with higher HDI scores (Fig. 4A; Table N in S1 File) according to the best performing model (i.e., lowest BIC). There was some evidence that GDP and population may also be correlated with Index scores. Population is negatively correlated with Index scores when analyzed by itself (Fig. 4B), and according to AIC scores, models including HDI and GDP performed slightly better than models with only HDI (with a positive correlation between GDP and Index scores; Table N in S1 File). However, adding population or GDP into a model with HDI only slightly increased the amount of variance explained by the model (Table N in S1 File, R^2^ values), suggesting that HDI, by far, is the best predictor of Index scores. Goal scores showed remarkably little correlation with each other, with only twelve pairs of goals showing highly significant correlations (p<0.01) and an additional three significant at p<0.05 (Fig. 5). The strongest correlations were for biodiversity with carbon storage and coastal protection, clean waters with artisanal fishing opportunities, and carbon storage with coastal protection.

## Discussion

Overall ocean health scored 68, improving only slightly compared to the 2012 Ocean Health Index score. The 2012 score is 7 points higher than what was estimated in the previously calculated Ocean Health Index [7], nonetheless this adjusted score still leaves ample room for improvement (Fig. 1). Food provision, natural products, tourism & recreation, and sense of place goals had the lowest scores globally, emphasizing the need to improve the sustainable extraction of products (for food or trade) and better protect and deliver cultural and recreational values derived from ocean ecosystems. Many countries on both coasts of central Africa and several in the Caribbean had the lowest Index scores, while remote (often uninhabited) islands in the Southern and South Pacific Oceans had the highest scores (Fig. 1; Table L in S1 File). Many populated countries also scored relatively high in many cases (e.g., Malta, New Zealand, Estonia, Australia, Denmark, and Norway all scored 77–81; see Table L in S1 File), but in all cases 14–18 points lower than the highest scoring uninhabited locations. As noted previously [7], the Index can produce high scores for both relatively unexploited and sustainably used locations.

Many of the lowest scoring countries (Fig 2, Table L in S1 File) are poorer and have a recent history of war, civil strife, ethnic conflict and/or dictatorship. Countries with those conditions generally do not have the resources or opportunity to address social or environmental needs effectively (e.g., [27]), and they cannot easily take the governance actions necessary to reduce social and environmental pressures. Substantial increases in global Ocean Health Index scores will be limited if such countries cannot escape from conditions that currently constrain their opportunities. However, economic growth alone is not likely to improve ocean health, as indicated by the lack of relationship between Index scores and GDP (Fig. 4).

Worldwide, Index scores remained largely unchanged from the 2012 assessment, with countries generally showing at most one to two points difference (Fig. 3, Table M in S1 File). This is not surprising, as the overall composite Index scores, especially at very large scales, should not change much from year-to-year unless dramatic negative (such as an environmental disaster) or positive (such as a large-scale conservation effort) changes occur in the system. Furthermore, data layers that do not have updated values dampen changes in scores. The countries with the largest change in Index scores were small regions where a relatively large area was set aside within a Marine Protected Area (South Georgia and South Sandwich Islands) or countries where individual goal scores dropped dramatically (e.g., natural products in Cook Islands) due to a large decrease in ornamental fish and shell harvest. Natural products, coastal protection, and species biodiversity were the only three goals with negative year-to-year changes (Fig. 3, Fig. A in S1 File), indicating that efforts to improve sustainable harvest of natural products, protect and restore coastal habitats, and better manage and protect biodiversity are key areas for mitigating losses and halting further declines in ocean health. Although the Index can help identify where changes are occurring, it cannot always explain why they are occurring, beyond flagging which data sources are driving the changes. The underlying causes of changes in those data sources require further investigation. For example, Croatia and Turkey saw large changes in their clean water goal scores (Fig. 3; Table M in S1 File), which the underlying data (http://www.ohi-science.org) reveal is due to sharp annual changes in marine debris. Whether these changes are due to actual changes in marine debris or to the quality of data reporting remains unknown, although the use of the same data source collected in the same way each year suggests the changes are real.

The lack of correlation for nearly all pairwise comparisons of goal scores (Fig. 5) suggests that there is low redundancy across goal indicators. In other words, a subset of goals would not easily deliver the same information, demonstrating the utility of measuring multiple facets of ocean health simultaneously through a range of public goals.

The complete absence of any negative relationships (Fig. 5) suggests that potential tradeoffs in the delivery of goals are not strong, at least at the country-level scale, or perhaps are country-specific such that a decline in food provision might cause a decline in coastal livelihoods & economies in certain countries, but not others. Most tradeoffs likely occur at sub-national scales and may take several years to emerge in the system, such that they would not be detectable by the Ocean Health Index with only a year or two of assessments. Regional assessments repeated through time offer a key tool for understanding the consequences of possible tradeoffs, and thus informing comprehensive ecosystem-based management.

We expect some tradeoffs to result from the pursuit of a goal, hampering the delivery of other goals. However, if the pressures generated from the activities associated with pursuing a goal are depressing all goals simultaneously, these simultaneous pressures may mask potential tradeoffs. The positive correlations between carbon storage, coastal protection, and biodiversity are primarily due to the large overlap in the data used (habitat condition and extent) to assess the goals. The correlation between clean waters and tourism & recreation is in part due to the dominant role that water pollution pressures play in driving scores for both goals. The other significantly positive correlations are not primarily due to shared data. The correlation between clean water and biodiversity suggests (intuitively) that conservation-focused goals of unpolluted waters and protected biodiversity tend to co-vary spatially. The other significant pairwise correlations are less intuitive. Future assessments should help shed more light on these relationships as additional years of data allow for longer-term temporal comparisons.

Although many data layers did not have updated values for the current assessment, there is still great utility in an annual re-calculation of the Index. Where and when actions are taken or changes in the system occur, the Index will reflect those changes. Without annual assessments, reporting these changes would be delayed and would limit the short-term feedback that decision makers need in order to assess if or how well actions are working. The single global scores for the Index and goals are not expected to change much year to year, but changes are expected at the regional scale, where most decisions are made, even when only a subset of the full set of data layers is updated. Annual calculations also help highlight where data are not being updated; if those goals or aspects of ocean health are important for our understanding of how oceans are changing, then identifying those gaps can help motivate future monitoring and assessment. Finally, annual assessments allow real-time incorporation of data providers’ revised estimates of data from previous years, as often occurs with FAO data used for natural products and food provision goals.

### Methodological changes

A key change in how Index scores were calculated and reported, compared to the first global assessment in 2012 [7], was the inclusion of 50 new reporting regions. Previously, these regions were aggregated into groups of primarily territorial holdings, but often the individual territories or islands are separated from each other by hundreds to thousands of kilometers and have different cultural and economic interactions with ocean ecosystems. Many are also relatively data-limited regions, which is why they were previously aggregated. To calculate Index scores for these regions, we developed a number of data gap-filling procedures (see S1 File). Consequently, even though scores are now reported for these regions, they generally have higher uncertainty than the scores for other reporting regions, so results should be interpreted with caution (see Fig. C in S1 File).

Methodological improvements in how the fisheries and mariculture sub-goals and the tourism & recreation goal were calculated provide more robust assessments of these goals than was previously possible. For all three cases, global area-weighted average scores increased relative to previous methods [7], although scores for individual countries showed decreases as well as increases. Thus, in these cases, previous data-limited approaches underestimated the overall likely health of these goals; future improvement in methods or data quality for other goals could have the opposite consequence, for example if more extensive or finer-scale data revealed greater levels of pollution or habitat loss. In either case, methodological improvements produce results with higher certainty. Therefore, although changes in assessed results highlight possible sources of error, and future methodological improvements may cause other small changes, imperfect but informed results are more useful than no information. Furthermore, for an Index to remain relevant and based on the best available science, it needs to adapt to improved data and scientific understanding. When such improvements occur, recalculating past scores with the new information, as we did here, allows for direct comparisons among years.

### Policy implications

Many countries around the world have enacted policy encouraging or mandating actions that promote sustainable ocean ecosystems in a way largely synonymous with how the Index defines healthy oceans. In particular, the European Union has set an objective of ‘good environmental status’ [5], and the United States and Australia both have stated objectives of ‘healthy oceans’ that focus on environmentally sustainable development [1,6]. As such, the Index provides a unique quantitative tool for assessing progress towards meeting these comprehensive objectives.

Looking forward, we have identified three ways to increase the utility of the Ocean Health Index in informing and guiding management decisions. First, continuing to conduct annual assessments will allow for deeper insights into longer-term trends and stronger attribution of change to specific actions in particular locations. Thus, over time the Index can help assess management effectiveness. Second, translation of our data gap filling assessments (Fig. C in S1 File) into guidance on priorities for additional monitoring to fill those gaps and more quantitative measures of uncertainty will provide managers with key information about what is known with greater and less certainty, allowing for more strategic planning and investment. This is a current focus of our research and will be reported elsewhere. Third, regional applications of the Index at sub-national, national, or multinational scales based on local data and indicators provides more accurate regional assessments than possible through global assessments such as the one reported here, and thus better guidance for local management. As the number of these regional assessments increases, an opportunity emerges to leverage the lessons learned and assessment methodologies from those efforts for new regional applications. Several regional assessments have recently been completed [12–13, 28], and others are underway.

## Conclusions

Our main intent with this study was to demonstrate that the Index can be calculated annually and thus fit within policy timelines, and that that it is possible to observe emerging, albeit incomplete, trends that are useful to managers and scientists. Given the emerging interest in the Index, there is also great value in regularly incorporating and presenting updated methods and results to provide improved estimates of health and so that ongoing and future regional assessments can take advantage of these updates. Country-specific results from this global assessment offer heuristic guidance on the status and trajectory of ocean health that can be used by policy makers; specific and smaller-scale management decisions would be best informed by regional assessments tailored to the best available science in the region. Ultimately, tracking the Ocean Health Index’s global results, along with those from other global indices and studies, is the only way to chronicle humanity’s progress toward achieving sustainable engagement with Earth’s natural systems.

## Supporting Information

S1 File Complete set of supplementary information, including Supplementary Methods, Tables A-N, and Figures A-C. (PDF)
