# Supplementary material for: Patterns and Emerging Trends in Global Ocean Health
Source: PLoS One. 2015 Mar 16;10(3):e0117863. doi: 10.1371/journal.pone.0117863 (PMC4361765; doi:10.1371/journal.pone.0117863)
Supplement: S1 File — (PDF) [file pone.0117863.s001.pdf]

Supplementary Information
Patterns and emerging trends in global ocean health

Halpern et al. PLoS ONE

1. Overview ... 4
2. Reporting Units ... 5
3. Reference Points ... 7
4. Goal Models ... 8
4.1 Food Provision ... 9
4.1.1 Fisheries ... 9
4.1.2 Mariculture ... 14
4.2 Artisanal Fishing Opportunities ... 15
4.3 Natural Products ... 15
4.4 Carbon Storage ... 16
4.5 Coastal Protection ... 16
4.6 Coastal Livelihoods and Economies ... 16
4.6.1 Coastal Livelihoods ... 18
4.6.2 Coastal Economies ... 18
4.7 Tourism & Recreation ... 18
4.8 Sense of Place ... 19
4.8.1 Iconic Species ... 20
4.8.2 Lasting Special Places ... 20
4.9 Clean Waters ... 20
4.10 Biodiversity ... 20
4.10.1 Species ... 20
4.10.2 Habitats ... 20
5. Specific Data Layers ... 20
5.1. Alien species ... 21
5.2. Artisanal fishing: high bycatch ... 21
5.3. Artisanal fishing: low bycatch ... 21
5.4. Artisanal fishing: management effectiveness and opportunity ... 21
5.5. Artisanal fishing: need ... 21
5.6. Chemical pollution: land-based inorganic ... 21
5.7. Chemical pollution: land-based organic ... 22
5.8. Chemical pollution: ocean-based ... 22
5.9. Coastal human population ... 22
5.10. Coastal land and ocean area ... 22
5.11. Commercial fishing: high bycatch ... 23
5.12. Commercial fishing: low bycatch ... 23
5.13. Convention on Biological Diversity (CBD) signatories ... 23
5.14. Convention on Biological Diversity (CBD) survey ... 24
5.15. Convention on International Trade in Endangered Species of Wild Fauna and Flora (CITES) signatories ... 24

5.16. Coral reefs ... 24
5.17. Country land area ... 24
5.18. Ecological integrity ... 24
5.19. Fertilizer trends ... 24
5.20. Fisheries catch data ... 25
5.21. Fisheries management effectiveness ... 25
5.22. Genetic escapes ... 25
5.23. Global Competitiveness Index (GCI) ... 25
5.24. Gross Domestic Product (GDP) ... 25
5.25. Habitat destruction: intertidal ... 25
5.26. Habitat destruction: subtidal hard bottom ... 25
5.27. Habitat destruction: subtidal soft bottom ... 25
5.28. Human Development Index (HDI) ... 26
5.29. Iconic species list ... 26
5.30. International arrivals ... 26
5.31. Labor force ... 26
5.32. Mangroves ... 26
5.33. Mariculture Sustainability Index (MSI): mariculture sustainability and mariculture regulations ... 26
5.34. Mariculture yield ... 26
5.35. Marine jobs: commercial fishing ... 27
5.36. Marine jobs: mariculture ... 27
5.37. Marine jobs: marine mammal watching ... 27
5.38. Marine jobs: tidal energy ... 27
5.39. Marine jobs: tourism ... 27
5.40. Marine protected areas and terrestrial protected areas ... 27
5.41. Marine revenue: aquarium trade fishing ... 27
5.42. Marine revenue: commercial fishing ... 28
5.43. Marine revenue: mariculture ... 28
5.44. Marine revenue: marine mammal watching ... 28
5.45. Marine revenue: marine renewable energy ... 28
5.46. Marine revenue: tourism ... 28
5.47. Marine species ... 28
5.48. Marine wages ... 29
5.49. Multispecies maximum sustainable yield (mMSY) ... 30
5.50. National percent unemployment ... 30
5.51. Natural Products exposure ... 30
5.52. Natural Products harvest ... 30
5.53. Natural Products value ... 31
5.54. Natural Products risk ... 31
5.55. Nutrient pollution ... 32
5.56. Ocean acidification ... 32
5.57. Pathogen pollution ... 32
5.58. Pesticides trends ... 32
5.59. Relative stock biomass ... 33
5.60. Rocky reef ... 33

5.61. Salt marsh ... 33
5.62. Sea ice ... 33
5.63. Sea level rise ... 33
5.64. Sea surface temperature (SST) anomalies ... 34
5.65. Seagrass ... 34
5.66. Sector Evenness ... 34
5.67. Soft-bottom subtidal condition ... 35
5.68. Spatially-allocated catch data ... 35
5.69. Stock exploitation status ... 36
5.70. Targeted harvest ... 36
5.71. Total population ... 36
5.72. Tourist days per stay ... 37
5.73. Trash pollution ... 37
5.74. Travel and Tourism Competitiveness Index (TTCI) ... 37
5.75. Travel and Tourism Direct Contribution to Employment ... 37
5.76. UV radiation ... 37
5.77. Worldwide Governance Indicators (WGI) ... 38
6. Data gap filling procedures ... 38
7. Supplementary Tables ... 43
Table G ... 43
Table H ... 51
Table I. ... 52
Table J. ... 61
Table K ... 64
Table L. ... 65
Table M ... 71
Table N ... 77
8. Supplemental Figures ... 78
Figure A. ... 78
Figure B. ... 79
Figure C ... 80
9. References ... 83

1. Overview

The Ocean Health Index aims to assess the current status and likely future state of ten widely held public goals for ocean ecosystems. These goals represent the full suite of benefits that people want and need from the ocean, including the traditional 'goods and services' people often consider (e.g., fish to eat, coastal protection from nearshore habitats) as well as benefits less commonly accounted for, such as cultural values or biodiversity, and others that are not identified as services, most notably livelihoods and coastal economies derived from the ocean.

The Index assesses the **present state** of each goal relative to a reference point, recent **trends** in current status, existing negative **pressures** that can stress the system and could thus make future delivery of benefits worse, and governance, institutional and ecological factors that provide social and ecological **resilience** to such pressures. Methods for calculating the Index, and the conceptual framework and rationale for how it is constructed, are detailed extensively elsewhere (Halpern *et al.* 2012). Here we focus on methodological changes and data updates, but also indicate where no changes were made.

To allow for more accurate comparisons to be made between Index scores for this assessment (2013) versus the first assessment (2012), we applied methods and used data sources from the 2013 assessment to re-calculate scores for 2012. Thus, comparisons between years will largely be due to actual changes in ocean health; differences between originally reported 2012 scores (Halpern et al. 2012) and recalculated 2012 scores represent changes due to methodological shifts. For a few data layers (noted in section 5), it was not possible to use the same data sources and/or data processing methods changed between assessments. For this reason, a small amount of difference in scores between 2012 and 2013 is due to methodological reasons caused by these underlying data. It is also important to note that not all data layers are reported through 2013, and so our assessment of current (i.e., 2013) ocean health incorporates the most recent year in each data layer (usually 2009 or later), and health for recalculated 2012 scores is one year prior to the most recent year of data. Details on which years are used for each data layer are provided in section 5 below and in Table H.

For those interested in the single score for global ocean health, we note there are two ways to derive this number: a) goal scores are averaged within a region (currently with equal weighting) to create an overall Index score that is then averaged with scores from other regions (weighted by the area of each region) to give the global score, or b) goal scores from every region are averaged (weighted by the area of each region) to give a global score for each goal, and then these global goal scores are averaged (currently with equal weighting) to create the single global Index score. The two methods produce slightly different single global scores because of the order in which area-weighted averaging is done. In method a) a high or low goal score is treated equally with the other nine goal scores when calculating the overall Index, and then area-weighting occurs when combining country-level Index scores to create a global Index score (i.e., area-weighting happens second), whereas in method b) a high or low goal score is weighted by area when calculating global goal scores before being combined into a single Index score (i.e., area-weighting happens first). A variation of this emerges when goals are not relevant to some countries (e.g., mangrove condition in temperate countries or livelihoods and

economies in uninhabited regions), where instead of a low or high score, a goal 'drops out' of assessment. Deriving the single global score from the area-weighted average of country-level Index scores (method a) best captures the core principle that ocean health is the result of interactions among the ten goals, in a location. If one were to first calculate global goal scores and then average across those scores (method b), these interactions would be ignored.

2. Reporting Units

The first global assessment in 2012 (Halpern *et al.* 2012) focused mainly on EEZ-level (i.e., country) results, but aggregated information from smaller regions and territorial holdings in cases where available data were particularly sparse. For the 2013 global assessment we instead report these regions separately and thus preserve higher resolution information whenever possible. For ease of reporting results, we call these regions countries, but they represent countries, semi-independent territorial holdings, and individual and occasionally grouped territories If higher resolution information was not available, we applied coarser resolution information equally across all smaller regions, through a variety of gap-filling methods, to allow calculation of Index scores for as many regions as possible. Both approaches make assumptions about how well data represent reality in data limited regions, but reporting more regions separately preserves higher resolution data when they are available. **Importantly, this approach creates Index scores for locations that are extremely data poor, and thus have very high uncertainty in the scores.** In section 6 below we detail the methods used to gap-fill missing data for the (often small) reporting units for which many data were missing.

A full list of reporting units used in the current assessment is provided in Table G. Table A lists the differences in reporting regions between 2012 and 2013 assessments.

Table A. Changes in reporting units from the 2012 global assessment (Halpern *et al.* 2012) and this current 2013 assessment.

Index 2012 region name	Index 2013 region name
Australian Tropical Territories	Cocos Islands
	Christmas Island
	Norfolk Island
Australian Southern Territories	Macquarie Island
	Heard and McDonald Islands
French Indian Ocean Territories	Mayotte
	Glorioso Islands
	Réunion
	Juan de Nova Island
	Bassas da India
	Ile Europa
	Ile Tromelin
French Southern Ocean Territories	Crozet Islands

	Amsterdam Island and Saint Paul Island
	Kerguelen Islands
British Southern Ocean Territories	Ascension
	Saint Helena
	Tristan da Cunha
	South Georgia and the South Sandwich Islands
	Falkland Islands
South Africa	Prince Edward Islands
	South Africa
British Caribbean Territories	Bermuda
	Turks and Caicos Islands
	Cayman Islands
	British Virgin Islands
	Anguilla
	Montserrat
Netherlands Caribbean Territories	Sint-Maarten
	Curaçao
	Bonaire
	Saba
	Sint-Eustasius
	Aruba
French Caribbean Territories	Guadeloupe and Martinique
	Northern Saint-Martin
French Polynesia	French Polynesia
	Wallis and Futuna
USA Pacific Inhabited Territories	Northern Mariana Islands and Guam
	American Samoa
USA Pacific Uninhabited Territories	Howland Island and Baker Island
	Wake Island
	Palmyra Atoll
	Johnston Atoll
New Zealand	Cook Islands
	Niue
	Tokelau
	New Zealand
Denmark	Faeroe Islands
	Greenland
	Denmark
United Kingdom	United Kingdom
	Jersey
	Guernsey

Spain	Canary Islands
	Spain
Portugal	Azores
	Madeira
	Portugal
India	Andaman and Nicobar
	India
Kiribati	Line Group
	Phoenix Group
	Kiribati
Norway	Bouvet Island
	Jan Mayen
	Norway
East Timor	East Timor
	Oecussi Ambeno
(not present)	Brunei

3. Reference Points

Reference points define what a perfect score for each goal represents and are thus fundamental to interpreting each goal score as well as the overall Index score. The conceptual approach to defining reference points and the importance of such decisions is described, with examples, elsewhere (Samhouri *et al.* 2012). The types of reference points used for each goal and sub-goal in the 2013 analysis are consistent with those in Halpern *et al.* (2012), but the way that reference points were defined changed in a few goals and sub-goals based on the availability of better data or a refined understanding of how to determine appropriate reference points. Table B below summarizes these changes; details for each change are provided in the goal model descriptions in section 4.

With repeated calculation of the Ocean Health Index over time, an additional issue may emerge when setting reference points for several of the goals; three are described here (natural products, coastal livelihoods & economies, clean waters). For natural products, if a country is just beginning to harvest a product, the peak value for harvest (which is used to set the reference point) will be in the latest year, and if in the next year there is more harvest, then the reference point will increase to that new peak and the score for the previous year will drop as measured against the new reference point. This shifting reference point will continue until harvest rates stabilize or begin to decrease. When more appropriate mechanistic models are developed for maximum sustainable harvest levels for these products, they can be used to set more informative reference points, but until then we must rely on oversimplified indicators (data-poor assessments for wild caught fisheries have struggled with this issue for decades). For coastal livelihoods & economies, we use a moving window reference point, such that by definition the reference point changes each year even if there is no change in any values from one year to the next. For clean waters, we rescale pollution layers to 110% of the maximum seen anywhere on the planet (we use 110% to allow for some increase over

time) – if future years have much higher pollution values than previously seen anywhere on the planet, then this rescaling would need to adjust for this new maximum value, affecting clean water scores for all countries.

Table B. Type of reference point used for each goal and sub-goal. Details for reference points that changed are provided in goal model descriptions in section 4. Additional information regarding selection of reference points can be found in Samhouri et al. (2012). Established targets are those determined through social or political processes; known targets are those set through scientific processes.

**Goal**	**Sub-goal: component**	**Reference point type (2012 assessment)**	**Reference point type (2013 assessment)**
Food Provision	Fisheries	Functional relationship	Updated; functional relationship
	Mariculture	Spatial comparison	Updated; spatial comparison
Artisanal Fishing Opportunities		Established Target	No change
Natural Products	Ornamental fish, shells, seaweeds & plants, sponges, corals§	Temporal comparison (historical benchmark)	No change, but improved data processing altered value of reference points
	Fish oil	Functional relationship	Updated; functional relationship
Carbon Storage		Temporal comparison (historical benchmark)	No change
Coastal Protection		Temporal comparison (historical benchmark)	No change
Coastal Livelihoods & Economies	Livelihoods: jobs	Temporal comparison (moving target)	No change
	Livelihoods: wages	Spatial comparison	No change
	Economies: revenue	Temporal comparison (moving target)	No change
Tourism & Recreation		Spatial comparison	Updated; spatial comparison
Sense of Place	Iconic Species	Known target	No change
	Lasting Special Places	Established target	No change
Clean Waters		Established target	No change
Biodiversity	Habitats	Temporal comparison (historical benchmark)	No change
	Species	Known target	No change

4. Goal Models

The models used to calculate the status and score for each goal are summarized in Table H. Below we describe in detail only those cases where changes were made from

previous approaches (Halpern *et al.* 2012). Details for goals not described below can be found in the Supplementary Information from the initial publication (Halpern *et al.* 2012). Most of the details provided below for the fisheries sub-goal and tourism & recreation goal are also provided in the main manuscript.

4.1 Food Provision

Changes were made to both sub-goals. Substantial changes were made to how the fisheries sub-goal was modeled and the data used to assess it. The primary change to the mariculture sub-goal was to modify the approach to setting the reference point (see Table B above and Kleisner *et al.* 2013). We note for clarity that this goal in principle includes food provided by artisanal scale fisheries, but because we currently know little about how much food comes from these sources relative to commercial fisheries and mariculture, it is not yet assessed in the current model. When such data become available, they will be included as a third sub-goal of the overall food provision goal.

4.1.1 Fisheries

Methods overview

This sub-goal model aims to assess the amount of wild-caught seafood that can be sustainably harvested, with sustainability based on multi-species yield, and with penalties assigned for both over- and under-harvesting. As such, one must establish a reference point at which harvest is both maximal and sustainable. Previously this reference point was derived from an estimate of multi-species maximum sustainable yield (*mMSY*; Halpern et al. 2012; Kleisner et al. 2013) based on an approach modified from Srinivasan et al. (2009) that used catch data only (i.e., a ‘data-limited’ approach where variables generally required by formal stock assessment methods are unknown, as is the case for most commercially exploited species). Recently, several new data-limited approaches have been developed to assess fisheries that leverage globally available information (Costello et al., 2012; Martell & Frœse, 2012; Thorson et al., 2013; Rosenberg et al. 2014). Building on these methodological advances, we developed a new approach to assessing food provision from wild caught fisheries that is based on estimating population biomass relative to the biomass that can deliver maximum sustainable yield (B/B_{MSY}) for each landed stock. The estimates of B/B_{MSY} were obtained by applying a model developed by Martell & Frœse (2012), and hereafter referred to as the “catch-MSY” method. The latter was chosen, among other data-limited methods available, based on simulation-testing showing that it most accurately predicted stock status for simulated stocks having a broad range of life history traits and different known sources of uncertainty (i.e., environmental stochasticity, length of available time-series, initial depletion, and temporal autocorrelation; Rosenberg et al., 2014). The catch-MSY approach improves upon the method used in Halpern et al. (2012) in that it: 1) leverages a mechanistic understanding of the connection between harvest dynamics and population dynamics and uses this to infer stock depletion levels (see also Thorson et al., 2013), 2) is an indicator of stock abundance (B) rather than catch, making it more directly informative of stock status, and 3) at least in some cases (i.e., those cases where the catch

trajectory is not a monotonic increase), can be applied to developing fisheries (whereas the previous approach assumed a perfect score in those cases).

Estimating B/B_{MSY} ratios

The catch-MSY method is based on the same assumptions used in many stock assessment models (Schaefer, 1954), namely that the change in a population's biomass depends on its biomass in the previous year and two population-specific parameters: the carrying capacity (*K*) and rate of population increase (*r*). The method estimates the status of a given population using landings time-series as proxies for biomass removals from the population, and using empirically derived relationships of relative peak to current catch values to estimate depletion at the end of the time series (Martell & Frœse, 2012). Then, a sampling procedure is used to estimate the distribution of values of *r* and *K* that are compatible with the estimated current depletion levels, and are constrained within the range that maintains viable population abundance and at the same time does not exceed the population's carrying capacity. In the original formulation of Martell & Froese (2013) the geometric mean *r* and *K* were used to derive an estimate of *MSY*. Rosenberg et al. (2014) modified this method by producing a biomass time series for each of the viable *r*-*K* pairs using the surplus production model. The arithmetic mean biomass time series was selected and the current year stock abundance (*B*) relative to the abundance that achieves MSY (B_{MSY}) produced a measure, B/B_{MSY}. Although model accuracy can be expected to improve by using species-specific "resilience" estimates found in the literature for parameter *r* (Musick, 1999), a compiled list of values for most of the species in our analysis is currently unavailable, therefore a uniform distribution for the *r* prior (as defined in Bayesian modeling, where a 'prior' assumption for a value is used to inform the model) was used instead.

Another parameter, final biomass, is estimated by the model and requires setting a prior distribution. The original catch-MSY method (Martell & Froese, 2012) is derived from stock reduction analysis (Kimura and Tagart 1982), whereby a time series of catch is combined with an estimate of the final biomass relative to an unfished or initial biomass state (i.e., depletion level) in order to estimate historical biomass trends. The model applies a rule to constrain the prior on final biomass based on catch in the final year relative to historical peak catch. However, we found this rule caused the model to frequently estimate a decline in B/B_{MSY} for stocks with declining catch in the final years of the time-series. Explorations suggested that these included cases of managed fisheries where reduced catch was due to declining effort rather than declining population biomass. On the other hand, removing this constraint and assuming a uniform prior caused the model to estimate that all stocks with declining catch in the final years were rebuilding (i.e., biomass was increasing due to a reduction in fishing pressure), which in turn was unrealistic. Therefore, based on these explorations, we assumed that the constrained prior on final biomass is more appropriate with fisheries that are poorly regulated, while places with stronger fisheries management regulations were best modeled using a uniform prior on final biomass. In order to discriminate between these two cases, we assigned a resilience score to each stock, S_r. We estimated B/B_{MSY} with a uniform prior for all stocks with a resilience score of 0.6 or above, and used the model with the original constrained prior for all stocks scoring below that.

Stock resilience scores

The resilience score (S_r) was calculated as the mean of the fisheries resilience score used within the resilience dimension of the fisheries goal calculation (see Table K) across all the regions where the stock was caught, weighted by the relative mean catch in each of the regions, as follows:

$$S_r = \sum_{z=1}^{n} r_z + \frac{c_z}{\sum c_j} \qquad \text{(Eq. S1)}$$

where n is the number of regions z (EEZs or high seas) in which the stock is caught, r_z is the fisheries resilience score assigned to that region, c_z is the mean catch of that stock in that region through time, and c_j is the mean catch of each of that stock in each of the regions. When the stock straddled the high seas, we assigned a resilience score to the regional fisheries management organizations (RFMOs) active in the area if it had an explicit mandate to manage that species. Otherwise the score was considered a 0.

We estimated the governance effectiveness of 15 different Regional Fisheries Management Organizations (RFMOs) based on publically available information. Broadly we scored each organization based on the presence of harvest controls (e.g. setting of MSY, gear management measures, rebuilding strategies, etc.) or harvest monitoring measures (e.g. record of vessels, record of IUU vessels, vessel monitoring systems, etc.). We also scored them for data reporting on catch, effort, gear, and bycatch as well as their transparency (e.g. public website, meeting minutes, etc.). In some cases we assigned partial scores. Scores were scaled according the maximum score of the RFMOs.

If a species is managed by more than one RFMO within a given FAO major fishing area, the score for that stock was determined as a weighted mean of the individual RFMO scores, weighted by the relative area they respectively cover within that FAO region. In coastal oceans, it was not feasible to research the list of species assessed and managed country by country, so the governance score applied equally to all species caught within that country's waters. This was based on the assumption that a poor fisheries governance score is given to places where most species are not well managed, while a high score implies some level of management towards sustainable fishing across all species.

We used fisheries governance scores calculated for other purposes (i.e., for the resilience dimension) to provide an objective and replicable rule for selecting which Catch-MSY model version (uniform vs. constrained prior) was used for each stock. We recognize there is no precedent for using the model this way, and further testing in the future would be valuable in order to establish more rigorous rules for how the priors are defined. Nevertheless, based on current knowledge and understanding, this seemed like the most appropriate option.

Spatial allocation of catch to EEZs

FAO data report commercial fishing landings (here used as a proxy for catch) by fishing country (i.e., for vessels, the flag state) and by major fishing area, but does not provide a finer spatial allocation. In order to assign scores to reporting regions for fisheries within their EEZ boundaries, we previously used the catch allocated as in

Watson et al. (2004). This spatial allocation method distributes FAO catches globally within a grid of one half-degree cell resolution, using maps of spatial distribution of commercial taxa, and allocating catches to fleets based on fishing access agreements. These half-degree resolution maps were then used to estimate the total catch per taxonomic group for each *Sea Around U*s Project reporting region, generally corresponding to EEZs, but in some cases to sub-regions of EEZs (www.seaaroundus.org; Table G). However, since the catch data and allocation rules have not been updated since 2006, we re-allocated FAO catch data from 2007-2011 into EEZs maintaining the relative proportion of stocks per EEZ per fishing area from 2006, but updating the values for total biomass per taxon per major fishing area and, where possible, updating the taxonomic resolution of the reporting (see data description in '5.68: Spatially-allocated catch data' below)

Goal model calculations

The status of wild caught fisheries (x_{FIS}) for each reporting region (*i*) in each year was calculated as the geometric mean of the stock status scores, *SS*. These scores were derived from B/B_{MSY} for each taxon landed within each FAO major fishing area (*A*, noted below) and weighted by its relative contribution to overall catch (*C*), such that:

$$x_{FIS} = \prod_{i=1}^{n} SS_i^{\left(\frac{C_i}{\sum C_i}\right)} \qquad \text{(Eq. S2)}$$

where *i* is an individual taxon and *n* is the total number of taxa in the reported catch for that country throughout the time-series, and *C* was calculated as the taxon average for each of our reporting regions in each year across the catch time-series since the first non-null record. We used the geometric weighted mean to account for the portfolio effect of exploiting a diverse suite of resources, such that small stocks that are doing poorly will have a stronger influence on the overall score than they would using an arithmetic weighted mean, even though their *C* contributes relatively little to the overall tonnage of harvested seafood within a given region. The behavior of the geometric mean is such that improving a well-performing stock is not rewarded as much as improving one that is doing poorly. We believe this indicator behavior is desirable because the recovery of stocks in poor condition requires more effort and can have more important effects on the system than making a species that is already abundant even more abundant. In this way, the score is not solely driven by absolute tonnes of fish produced and accounts for preserving the health of a diversity of species.

Because many fish populations straddle the boundaries of EEZs, we applied the catch-MSY model to catch aggregated within each major fishing area *A*. These values of stock status were then assigned to our reporting regions, weighted by their relative mean catch in that reporting region's catch (note that for a geometric mean weights appear in the exponent). This differs from the previous iteration, where the catch stream from each *Sea Around Us Project* reporting region (or EEZ) was analyzed separately. Any aggregation method will be biased in some way, but populations with the largest catches are most often straddling stocks, so a bias in assessments due to erroneous aggregation of catch could occur more often with cosmopolitan species that include small, sedentary (i.e.,

patchily distributed) populations that are less likely to play a dominant role in a country's fisheries.

Based on the ISSCAAP convention for taxon codes (http://www.fao.org/fishery/collection/asfis/en), 6 levels of taxonomic aggregation (*g*) are defined, from 6 (species) to 1 (order or higher) (see '5.68. Spatially-allocated catch data' for more details). The catch-MSY model was only run when catch was reported at the species level, i.e., taxon group level 6, as the time-series of catch across miscellaneous taxa is unlikely to fit required model assumptions. Overall, we were able to assess a total of 1874 stocks. The estimated species level values of B/B_{MSY} were used to derive a stock status score, *SS*, such that the best score is achieved for stocks at $B/B_{MSY} = 1$, with a 5% error buffer, and it decreases as the distance of *B* from B_{MSY} increases, due to under- or over-exploitation. For each species reported, within each major fishing area *A*, SS was calculated as:

$$SS_{A,g=6} = \begin{cases} \mathrm{B/B}_{MSY} & \text{if} \quad \mathrm{B/B}_{MSY} < 0.95 \\ 1 & \text{if} \quad 0.95 \le \mathrm{B/B}_{MSY} \le 1.05 \\ \max\{1-\alpha(\mathrm{B/B}_{MSY} - 1.05), \beta\} & \text{if} \quad \mathrm{B/B}_{MSY} > 1.05 \end{cases} \quad \text{(Eq. S3)}$$

where, for $B/B_{MSY} < 1$ (-5% buffer), *SS* declines with direct proportionality to the decline of *B* with respect to B_{MSY}, while for $B/B_{MSY} > 1$ (+5% buffer), *SS* declines at a rate α, where α = 0.5, so that as the distance of *B* from B_{MSY} increases, *SS* is penalized by half of that distance. For $B/B_{MSY} > 1.05$, β is the minimum score a stock can get, and was set at $\beta = 0.25$. The α value ensures that the penalty for under-harvested stocks is half of that for over-harvested stocks (α = 1.0 would assign equal penalty). The β value ensures stocks with $B/B_{MSY} > 1.4$ due to, for example, an exceptionally productive year, are not unduly penalized, and also recognizes that it is much easier to improve the goal score when stocks are under-harvested (i.e., increase fishing pressure) than it is when populations are over-harvested and need to be rebuilt. Both parameters α and β were chosen arbitrarily because there is no established convention for this particular approach. Thus, consistent with previous work (Halpern et al. 2012), countries are rewarded for having wild stocks at the biomass that can sustainably deliver the maximum sustainable yield, +/-5% to allow for measurement error, and are penalized for both over- or under-harvesting.

For taxa reported at a higher level than species, we developed a method to account for coarser resolution data. The distribution of the species-based estimates within the same fishing area and year was used to generate missing scores. An increasing penalty was applied for increasingly coarser taxonomic reporting, as this is considered a sign of minimal monitoring and management, so that, for a given taxonomic aggregation *g* (when g<6), a proxy value for B/B_{MSY} was estimated as follows:

$$\mathrm{B/B}_{\mathrm{MSY}\,A,g<6} = \begin{cases} 0.01 * \mathrm{median}\{\mathrm{B/B}_{\mathrm{MSY}\,A,g,\forall g=6}\} & \text{if} \quad g = 1 \\ 0.25 * \mathrm{median}\{\mathrm{B/B}_{\mathrm{MSY}\,A,g,\forall g=6}\} & \text{if} \quad g = 2 \\ 0.50 * \mathrm{median}\{\mathrm{B/B}_{\mathrm{MSY}\,A,g,\forall g=6}\} & \text{if} \quad g = 3 \\ 0.80 * \mathrm{median}\{\mathrm{B/B}_{\mathrm{MSY}\,A,g,\forall g=6}\} & \text{if} \quad g = 4 \\ 0.90 * \mathrm{median}\{\mathrm{B/B}_{\mathrm{MSY}\,A,g,\forall g=6}\} & \text{if} \quad g = 5 \end{cases} \quad \text{(Eq. S4)}$$

The resulting value was then used to obtain the stock status score as shown in equation S2. Previously (Halpern et al. 2012), reporting quality was penalized with a taxonomic reporting coefficient, T_c; we do not use this parameter here as it would be a double-penalty for data reporting quality.

Model limitations

Both the current and previous (Halpern et al. 2012) methods are based on single-species assessments of stock status and thus cannot predict the effect of multi-species interactions. Previously, the sum of single-species MSY was reduced by 25% to obtain a more precautionary estimate of multi-species MSY that might account for the effect of these interactions. The current approach adopts $B=B_{MSY}$ as a single-species reference point, which by various assessment frameworks is considered very conservative (e.g., Frœse et al. 2011), and the fact that the single-species values are aggregated using a geometric mean ensures that some multi-species effects may influence the scores. Nonetheless, a better understanding of the emerging effects of fishing various species at their reference levels would be desirable and will hopefully be possible in the future.

Despite the fact that invertebrates represent 12 of the top 17 species for global caught biomass, and represent the dominant stocks in many EEZs, stock assessment approaches for these taxa are poorly developed. The catch-MSY approach was applied to invertebrates even though the model developers only tested it on fish (Martell & Forese, 2012). Part of the challenge in broadly testing this approach on organisms other than fish is the lack of a large enough collection of invertebrate assessments to use for validation testing (e.g., only 17 invertebrate assessments are currently available in the RAM2 Legacy stock assessment database, less than half of which include estimates of B_{MSY}; Ricard et al. 2012).

Even though the openly accessible RAM Legacy database includes a large collection of B/B_{MSY} estimates from formal stock assessments across many FAO fishing areas, we were unable to include these in our calculations as there were <40 values for 2008 or later. When more updated versions of the database are made public, we will most likely be able to replace data-poor estimates with values from formal assessments.

This approach captures whether stocks have been historically well managed, but it is worth noting that this calculation does not directly measure current food production, contrary to the previous formulation that compared current catch to MSY.

It is also worth noting that, similar to the previous version, current management does not influence the present status, but it does influence the likely future state, through the resilience dimension and potentially through the trend, at least for highly dynamic stocks with quick response to management.

4.1.2 Mariculture

We modified the model, developed previously (Halpern et al. 2012), for this current assessment based on explorations and analyses described elsewhere (Kleisner *et al.* 2013). A basic problem facing assessments of mariculture is the lack of an ecologically- and socially-based reference point for the potential yield of every suitable mariculture species for every type of geographic habitat and location and accounting for

every other use of the oceans that would limit space for mariculture. Determining such reference points for every country at global scale is a daunting challenge, not only because so much information is lacking, but also because species, genotypes and habitats are likely to change. Consequently we based the reference point on proxy information. The previous assessment (Halpern et al. 2012) standardized regions relative to the region with the highest observed production density after the sustainability coefficient was applied (China). This decision was based on the assumption that all coastal area in each region was equally developed and able to produce the same density of mariculture as the reference country and that maximum potential productivity per unit of area is similar across ecosystems and countries. This caused countries with extensive coastline but low population density, such as Canada, to be penalized. The new approach instead bases the reference point on harvested tonnes per coastal inhabitant (i.e., those within 25km of the coast), under the assumption that production depends on the presence of coastal communities that can provide the labor force, infrastructures, and economic demand to support the development and economic viability of mariculture facilities. Thus, two regions with an equal number of coastal inhabitants harvesting an equal tonnage of cultured seafood will score the same, as productivity is commensurate to each region's socio-economic potential to develop mariculture. Stated another way, mariculture development is assumed to scale proportionally with coastal population, which is a proxy for potential logistic limitations to farm development, e.g., presence of infrastructures, coastal access, and locally available workforce. Given the very high skew in the status values per country, we set the reference point to the 95th percentile region (Thailand), with all regions above that value set to a status score = 1.0. Updates to data used for this goal are detailed below in Section 5.

4.2 Artisanal Fishing Opportunities

No changes to the goal model were made. Updates in data used for this goal are detailed below in Section 5.

4.3 Natural Products

The sustainability component for fish oil was changed. All other products were calculated as before, except for improvements in data gap-filling as described here and in section 6 below. Updates in data used for this goal are detailed below in Section 5, but we note here that past years' data were revised by FAO and so some changes are due to updated data.

This goal model calculates overall status by weighting the status of sustainable harvest of each product (tonnes) by its proportional value (US dollars) relative to other harvested products. Because of inconsistencies with how data are reported to FAO on the harvest and monetary value of each product (many countries report only one or the other of the two measures in a given year), there are many cases where harvest data but no value data are reported, and equal numbers of cases with value data but no harvest data. These mismatches in reporting would cause products to 'drop out' of the calculation of overall status, thus losing real data. Because a reported US dollar value must come from

the harvesting of a product, and similarly the reporting of harvested tonnage implies some economic value of that product, we developed several methods to fill these gaps.

To address this and other issues related to inadequate data reporting, we used a correlative model to estimate missing tonnes or US dollar values, assuming that the relationship was linear. This was done after smoothing harvest data (both tonnes and US dollars) with a 4-year moving window (skipping missing values rather than treating them as 0) to help fill gaps and minimize the potential effect of under- or over-reporting of annual harvest. This process required a minimum of 4 years of data from both data layers. We also had to gap fill a few of the sustainability scores for products when yield was reported but no habitat existed in the region with which to calculate exposure (see Halpern et al. 2012). In these cases we used the regional average across all other regions with sustainability scores (ornamentals: 2013 filled value=0.946, 2012 filled value=0.942, N=28; corals: 2013=0.389, 2012=0.383, N=28; sponges: 2013=0.974, 2012= 0.974, N=22; shells: 2013=0.916, 2012= 0.911, N=19; seaweeds: 2013=0.817, 2012=0.823, N=28).

To determine the relative contribution of each product to the overall status of the goal, we used the ratio of the maximum US dollar value for a product (from the smoothed, gap-filled data) across all years of data for the product, relative to the sum of maximum values for all products harvested in the country.

4.4 Carbon Storage

No changes to the goal model were made. Mangrove data used in the goal model were processed differently than described in Halpern et al. 2012: data now include 1km inland in addition to 1km offshore; other data that were updated are detailed below in Section 5.

4.5 Coastal Protection

No changes to the goal model were made. Data that were updated are detailed below in Section 5.

4.6 Coastal Livelihoods and Economies

A few simplifying changes to the goal model were made, and some clarifications from the descriptions provided in Halpern et al. 2012 are provided. Coastal livelihoods and economies models do not include any measure of petrolium extraction (which was incorrectly stated in the supplementary information of Halpern *et al.* 2012), as we do not consider these practices to be related to the biophysical state of the system and, since they rely on a non-renewable resource, they are inherently unsustainable.

Adjustments included in the 2013 assessment were simplified from those used in the 2012 calculations. Wages data were divided by the inflation conversion factor for 2010 so that wage data across years would be comparable in 2010 US dollars (inflation conversion factors were downloaded from http://oregonstate.edu/cla/polisci/sahr/sahr). These data were also multiplied by the purchasing power parity-adjusted per capita GDP (PPPpcGDP). Jobs data, which were not updated, were adjusted by dividing by percent

employment for the corresponding year: (1 – percent unemployment) * total labor force (data from the World Bank). Data from the International Labour Organization (ILO) that were previously used to modify wages data were removed from the 2013 assessment because they were redundant. Revenue data were adjusted by dividing by GDP per country (reported in 2013 USD; data from the World Bank).

As was the case in the previous Index assessment of this goal, three reference points are calculated: for the livelihoods sub-goal, jobs had a moving target temporal comparison and wages had a spatial comparison; and for the economies sub-goal, revenue had a moving target temporal comparison.

The same nine sectors for marine jobs, marine wages, and marine revenue reported in the 2012 analysis were included in the current 2013 analysis, with data updated when available (Table C). No changes were made to the multipliers used in the 2012 analysis.

Table C. Sectors for which data were available (A) and updated (U) for 2013 calculations for each of the three measures for this goal.

Sector	Jobs data	Wages data	Revenue data
Tourism	A	A, U	A, U
Commercial fishing	A	A, U	A
Marine mammal watching	A		A
Aquarium fishing			A, U
Marine renewable energy	A		A
Mariculture	A		A, U
Transportation & shipping		A, U	
Ports & harbors		A, U	
Ship & boatbuilding		A, U	

A note on marine renewable energy, which is an expanding industry with many positive and negative effects on humans and ecosystems beyond the actual energy harnessed (Lam & Roy 2014). Marine renewable energy includes five major technologies: tidal barrages, marine currents, waves, ocean thermal converters and salinity gradients. In global-scale Index assessments, marine renewable energy includes data only for the largest tidal barrage plants, as these data are available. These data are represented only as marine revenue and marine jobs at two plants capturing tidal energy: La Rance Tidal Power Station (France) and Annapolis Royal Generating Station (Canada). These two plants have large enough energy production to be included in the United Nations Energy Statistics database (http://data.un.org/Data.aspx?d=EDATA&f=cmID%3aEO) and have

multiple years of data (Sihwa Lake Tidal Power Station in South Korea, the world's largest tidal power station only became operational in 2011 and could not be included for this reason). Marine renewable energy can also act as pressures and resilience on other goals within OHI; the details of these tradeoffs are very site-specific and best incorporated at regional assessment scales (e.g. Elfes et al. 2014) where information may be available on interactions over time.

4.6.1 Coastal Livelihoods

Updates in data used for this sub-goal are detailed below in Section 5. For marine wages, all sectors were updated. For marine jobs, no data were updated.

4.6.2 Coastal Economies

Data for marine revenue were updated in only three of the six sectors for which data were available in 2012; the updated sectors were aquarium fishing (i.e., ornamental fish harvest), mariculture, and tourism (and sectors for which updated data were not available were commercial fishing, marine mammal watching, and tidal energy; see 5.41-5.46 below, and Table C above).

4.7 Tourism & Recreation

The tourism & recreation goal aims to capture the number of people, and the quality of their experience, visiting coastal and marine areas and attractions. Although coastal tourism industries can be important contributors to coastal economies, the tourism & recreation goal is assessed separately from its economic benefits, which are reported in the coastal livelihoods & economies goal. Few non-economic indicators of tourism and recreation exist at the global scale, and thus the original approach in the 2012 assessment approximated this goal by measuring the number of international tourists arriving by airplane to coastal regions, adjusting these values to the region’s population density to allow comparability across regions, and accounting for their average length of stay. This approach was sub-optimal in part because it did not account for domestic tourism, which is a large part of tourism in many regions, especially large regions such as Canada, Russia, Australia and the USA. In the 2013 assessment we develop a different model to capture the tourism and recreation goal, one that better accounts for both international and domestic tourism.We used employment in the tourism sector as a reasonable proxy measure for the total number of people engaged in coastal tourism and recreation activities. Employment within this sector should respond dynamically to the number of people participating in tourist activities, based on the assumption that the number of hotel employees, travel agents and employees of other affiliated professions will increase or decrease with changing tourism demand within different regions.

Ideally there would be data available specifically for employment in coastal tourism industries, however the best data available at a global scale report total number of jobs, not just coastal jobs, within the travel and tourism industries (World Travel and Tourism Council (WTTC)). These data include jobs for both leisure and business that are directly connected to the tourism industry, including accommodation services, food and

beverage services, retail trade, transportation services, and cultural, sports and recreational services, but exclude investment industries and suppliers (WTTC 2013). Unfortunately it was not possible to determine the proportion of jobs affiliated with strictly leisure tourism. However, some (unknown) proportion of business travelers also enjoy the coast for leisure during their visit to coastal areas, such that we assumed all travel and tourism employment was related to tourism and recreation values. Regional applications of the Index can make use of better-resolved data and more direct measures of tourism, as has been done within the US West Coast (Halpern *et al.* in review), where data for participation in coastal recreational activities across 19 different sectors were available.

To best capture coastal travel and tourism employment using WTTC data, we calculated the proportion of direct employment in the tourism industry relative to total labor force (E_t). As in 2012, we used the tourism competitiveness index (TTCI) from the World Economic Forum (WEF 2013) to capture the sustainability (S_t) of the tourism industry.

Therefore, the status of the tourism & recreation (x_{TR}) is:

$$x_{TR} = E_d \cdot S_t , \qquad \text{(Eq. S5)}$$

where E_d is defined as the proportion of employees directly involved in the travel and tourism industry (E_t) relative to the total employees in that region, calculated as the country's total labor force (L_t) corrected by the percent of the population that is unemployed (U_t), such that:

$$E_d = \frac{E_t}{L_t - (L_t \times U_t)} . \qquad \text{(Eq. S6)}$$

Because we do not know how employment patterns vary geographically within sectors for each region, we assume that the proportion employed in the tourism industry is the same in coastal areas as it is away from the coast, and thus E_i is the same whether applied solely to coastal areas or to the entire region. As such, the status of this goal could be increased by increasing a) the number people employed in the tourist industry relative to changes in the labor force and unemployment within the whole region or b) the sustainability of tourism and recreation (as measured by the TTCI).

Data for E_t existed for 148 regions (i.e., data were missing for 63 reporting regions; see Fig. S3). To fill the gaps for missing regions we used final goal scores rather than E_t values for the 148 regions and then followed the gap-filling guidelines described in section 6 of the Supplementary Information. We avoided gap-filling the E_t data layer because doing so created cases where the number of tourism jobs exceeded the reported labor force (data from the World Bank).

4.8 Sense of Place

No changes to the goal model were made.

4.8.1 Iconic Species

Updates in data used for this goal are detailed below in Section 5.

4.8.2 Lasting Special Places

Updates in data used for this goal are detailed below in Section 5.

4.9 Clean Waters

No changes to the goal model were made. Updates in data used for this goal are detailed below in Section 5.

4.10 Biodiversity

No changes to the goal model were made.

4.10.1 Species

Updates in data used for this goal are detailed below in Section 5 (see 5.47. Marine species). The 2012 scores for the species sub-goal (and therefore the biodiversity goal) were updated to reflect the release of extinction risk estimates for significantly more species during this past year. Actual changes in risk status from last year to this year occurred for only 15 of 6080 species, primarily because species are rarely re-assessed; in other words, only when new species are added, or previously assessed species are reassessed, can the status score of this sub-goal change.

4.10.2 Habitats

No changes to the goal model were made. Updates in data used for this goal are detailed below in Section 5 (see 5.16. Coral reefs; 5.32. Mangroves; 5.61. Salt marsh; 5.62. Sea ice; and 5.65. Seagrass).

5. Specific Data Layers

This section provides detailed descriptions for data layers updated or new to the 2013 assessment, and notes those that have not changed. In addition, Table I lists all data layers used in the 2013 assessment and indicates which were new variables or data sources, which were used in the first assessment (Halpern *et al.* 2012) and have been updated for the 2013 assessment (i.e., the source provided more recent data than those used previously; Halpern *et al.* 2012), and which were used previously and had not been updated for the 2013 analysis. Also indicated in Table I are data used previously but not included in the 2013 analysis. In all cases the data have been updated to account for the new reporting regions used for the 2013 assessment.

5.1. Alien species

Update: no update possible; values from 2012 Index used.

5.2. Artisanal fishing: high bycatch

Update: no update possible; values from 2012 Index used.

5.3. Artisanal fishing: low bycatch

Update: no update possible; values from 2012 Index used.

5.4. Artisanal fishing: management effectiveness and opportunity

Update: no update possible; values from 2012 Index used.

5.5. Artisanal fishing: need

Update: additional year(s) data.
Description: This parameter is estimated by the per capita purchasing power parity (PPP) adjusted gross domestic product (GDP), i.e. GDPpcPPP, as described previously (Halpern *et al.* 2012; the model treats need as 1- GDPpcPPP). Index Mundi (www.indexmundi.com describes GDP and PPP as:

> "GDP is the value of all final goods and services produced within a nation in a given year. A nation's GDP at purchasing power parity (PPP) exchange rates is the sum value of all goods and services produced in the country valued at prices prevailing in the United States in the year noted. This is the measure most economists prefer when looking at per-capita welfare and when comparing living conditions or use of resources across countries. The measure is difficult to compute, as a US dollar value has to be assigned to all goods and services in the country regardless of whether these goods and services have a direct equivalent in the United States (for example, the value of an ox-cart or non-US military equipment); as a result, PPP estimates for some countries are based on a small and sometimes different set of goods and services. In addition, many countries do not formally participate in the World Bank's PPP project that calculates these measures, so the resulting GDP estimates for these countries may lack precision. For many developing countries, PPP-based GDP measures are multiples of the official exchange rate (OER) measure. The differences between the OER- and PPP-denominated GDP values for most of the wealthy industrialized countries are generally much smaller."

Updated GDPpcPPP calculations were available through 2012 from the World Bank (http://data.worldbank.org/indicator/NY.GDP.PCAP.PP.CD) and are reported in 2012 US dollars for all years. For the three EEZs that fall within the China region (China, Macau, and Hong Kong), we combined the values using a population-weighted sum.

5.6. Chemical pollution: land-based inorganic

Update: no update possible; values from 2012 Index used.

5.7. Chemical pollution: land-based organic

Update: additional year(s) available
Description: To allow for more direct comparison between assessments from 2012 (input data from 2003-2006, as was done previously) and 2013 (input data from 2007-2010, the most recent available), we recalculated this layer using updated data but the same methods as before. In particular, we used FAO data on annual country-level pesticide use, averaged over the time periods, with missing values filled in by regression between fertilizer (see nutrient pollution, below) and pesticides when possible, and when not possible with agricultural GDP as proxy (see Halpern et al. 2008 for methods). These country-level values were then dasymetrically distributed over a country's landscape using global land cover data from the years 2005 (for the 2003-2006 time period) and 2009 (for the 2007-2010 time period), derived from the MODIS satellite at ~500m resolution (following methods described in Halpern et al. 2008 but with updated MODIS data not available previously). These values were then aggregated by ~140,000 global basins (developed for and presented in Halpern et al. 2008), and diffusive plumes were modeled from each basin's pourpoint. The final non-zero plumes (about ~76,000) were aggregated into ~1km Mollweide (wgs84) projection rasters to produce a single plume-aggregated pollution raster. These raw values were then log(x+1) transformed and normalized to 0-1. A simple visual and pixel-count comparison of the agriculture land cover classes shows that, globally, these classes have not changed much during the two time periods (at the 500m resolution of the data). However, global pesticide consumption has shown a small, but significant increase globally (roughly 4-8% over our time period).

5.8. Chemical pollution: ocean-based

Update: no update possible; values from 2012 Index used.

5.9. Coastal human population

Update: additional year(s) available
Description: Coastal population, defined as the total population inland of 25 miles, was based on the Gridded Population of the World (GPW) Population Density Grid Future Estimates, v3. These data were accessed from the Center for International Earth Science Information Network (CIESIN)/Columbia University (CIESIN & CIAT 2005). Rasters of population density (number of people per square kilometer) at 2.5 arc-minute resolution were globally available for 2005, 2010, and 2015. Years in between those provided were temporally interpolated. For instance, d2013 = 0.4 * d2010 + 0.6 * d2015. For each year, rasters were projected to 1 km Mollweide, converted to units of total population per cell at the new resolution and summed per region within the 25 mi inland area For the following 19 small island regions, coastal populations were set to the total population due to lack of sufficient resolution from the input rasters (see layer 5.71. Total population): Amsterdam Island and Saint Paul Island, Bassas da India, Bouvet Island, British Indian Ocean Territory, Clipperton Island, Crozet Islands, Glorioso Islands, Heard and McDonald Islands, Ile Europa, Ile Tromelin, Jan Mayen, Johnston Atoll, Juan de Nova Island, Kerguelen Islands, Macquarie Island, Palmyra Atoll, Prince Edward Islands, South Georgia and the South Sandwich Islands, Wake Island.

5.10. Coastal land and ocean area

Update: additional year(s) available

Description: The same land-sea mask was used as last year. Regions this year are more spatially refined and based on a newer version (v7) of exclusive economic zones (VLIZ 2012). All unique exclusive economic zones (EEZ) were used to define a region, except for the following groupings: Trindade into Brazil, Easter Island into Chile, Galapagos Islands into Ecuador, and Alaska and Hawaii into USA. Splitting has since occurred for Antilles (Sint-Maarten, Curaçao, Bonaire, Saba, Sint-Eustasius, Aruba), and Malaysia has been reapportioned to accommodate the new EEZ of Brunei. Many borders have been redrawn, such as the removal of UK claims around Cyprus. Gaps and extensions between this EEZ file and our land-sea mask were resolved through GIS operations (buffer, erase, and polygon neighbor analysis). Ocean area per region was calculated using geodesic area calculations on the region polygons in geographic coordinates. We exclude from regions the inland EEZs of the Caspian Sea and any disputed areas.

5.11. Commercial fishing: high bycatch

Update: additional year(s) available
Description: The most recent spatially-explicit global estimates of catch by gear type were developed and used in Halpern et al. (2008), where catch was allocated to 5 different gear types at half-degree resolution, and have not been updated since. To estimate changes in this pressure, we used the spatially-allocated catch data (see 5.68 below) and calculated the percent change in total annual catch per reporting region as the difference between the most recent reporting period (2009 to 2011) and the period (1999 to 2003) previously used by Halpern et al. (2008), divided by the original period (1999 to 2003). Each of the three high-bycatch 1km raster layers (Halpern et al. 2008) was then multiplied by the percent change specific to each *Sea Around Us* reporting region to create estimates of current spatially-explicit catch by gear type. Although this calculation requires the unlikely assumption that the proportional amount of catch per *Sea Around Us* reporting region has remained the same over the past 10 years, we felt it better to make this assumption than to assume fishing pressure has not changed at all.

Overall pressure per gear class per Index reporting region was then calculated as the mean of all 1km pixel values within the region for each of the two time periods. The maximum possible score (which is used to rescale all values 0-1) was then determined as 110% of the maximum regional score across either time period, such that all regions were rescaled to the same value. For each pressure layer the maximum came from the previous time period (1999-2003; Halpern et al. 2008; Halpern et al. 2012). As was done previously (Halpern et al. 2012), high bycatch commercial fishing was then measured as the average of demersal destructive (e.g., trawl), demersal non-destructive high bycatch (e.g., pots, traps) and pelagic high bycatch (e.g., long-line) gear.

5.12. Commercial fishing: low bycatch

Update: additional year(s) available
Description: Methods for this pressure layer were identical to those described above (5.11) for commercial fishing: high bycatch, except the two low bycatch commercial fishing categories were used (demersal low bycatch, e.g., hook and line; and pelagic low bycatch, e.g., purse seines) and averaged to create the single low bycatch pressure layer.

5.13. Convention on Biological Diversity (CBD) signatories

Update: no update possible; values from 2012 Index used.

5.14. Convention on Biological Diversity (CBD) survey

Update: no update possible; values from 2012 Index used.

5.15. Convention on International Trade in Endangered Species of Wild Fauna and Flora (CITES) signatories

Update: additional year(s) available
Description: An updated list of CITES signatories was accessed in April 2013 (http://www.cites.org/eng/disc/parties/chronolo.php). Three additional countries had joined as CITES signatories since the 2012 Index assessment: Bahrain, Maldives, and Lebanon.

5.16. Coral reefs

Update: no update possible; values from 2012 Index used.

5.17. Country land area

Update: updated data layer based on new Ocean Health Index reporting units (EEZ boundaries).
Description: This value represents the total land area (not including inland lakes or EEZs) within each country. Updated land area data for 2012 were extracted from ESRI (www.esri.com) and any gaps were filled using data for 2012 downloaded from the CIA World Factbook (CIA 2013).

5.18. Ecological integrity

Update: additional year(s) available
Description: This layer is based on the status score for the species sub-goal in the biodiversity goal. Details on updated data used here are provided in '5.47. Marine species' below

5.19. Fertilizer trends

Update: additional year(s) available
Description: Updated fertilizer consumption data were available through 2010 from FAO's statistical database FAOSTAT ((http://faostat3.fao.org/faostat-gateway/go/to/browse/R/*/E). Data were summed across all fertilizer compounds and reported in metric tons. Upon inspection the data included multiple 0 values that are most likely data gaps in the time-series, so they were treated as such and replaced with NA. In addition, regions with only 1 data point and regions where the most recent data point was prior to 2005 were excluded. The data gaps were then filled using coastal population trends for the corresponding reporting region. Uninhabited countries were assumed to have no fertilizer use and thus excluded. Nine regions were inhabited but had no fertilizer or population data. Of these, two were considered close enough to large countries to receive influence of their pollution and were gap-filled using regional trends (i.e., Juan da Nova and Glorioso Islands), and the remaining 7 were considered too remote, hence their trend was assumed to be 0. For the 2013 assessment, the 2010 values were used as the most recent year. When data for 2010 was missing, the trend for 2013 is identical to the trend for the 2012 assessment.

5.20. Fisheries catch data

Update: new data layer
Description: Catch per taxon values from the spatially allocated catch-data (layer 5.68) were summed within each FAO major fishing area (www.fao.org/fishery/area/search/en) to produce a single time-series from 1980 to present for each taxon within a given fishing area. These data are used to run the model estimating B/B_{MSY} (i.e., "catch-MSY", as described in the fisheries sub-goal model above, section 4.1.1; Martell & Frœse, in press). This time frame provides sufficient data for the Catch-MSY model to calculate stock status, although it could mask some fisheries dynamics as fishing-driven stock depletion had already occurred by 1980 for many stocks.

5.21. Fisheries management effectiveness

Update: no update possible; values from 2012 Index used.

5.22. Genetic escapes

Update: no update possible; values from 2012 Index used.

5.23. Global Competitiveness Index (GCI)

Update: no update included

5.24. Gross Domestic Product (GDP)

Update: additional year(s) available
Description: These data are used in the economies sub-goal of the coastal livelihoods and economies goal to adjust revenue data. Updated GDP data through 2012 (reported in 2012 US dollars) were accessed from the World Bank (data.worldbank.org/indicator/NY.GDP.MKTP.CD). For the three EEZs that fall within the China region (China, Macau, and Hong Kong), we combined the values using a population-weighted average.

5.25. Habitat destruction: intertidal

Update: additional year(s) available
Description: This data layer is derived from the 1km buffer coastal population data, described above.

5.26. Habitat destruction: subtidal hard bottom

Update: no update possible; values from 2012 Index used.

5.27. Habitat destruction: subtidal soft bottom

Update: no update possible; values from 2012 Index used.

5.28. Human Development Index (HDI)

Update: no update included.

5.29. Iconic species list

Update: no update possible; values from 2012 Index used, with new reporting regions receiving values from previous aggregated reporting region.

5.30. International arrivals

Update: no longer used

5.31. Labor force

Update: new data layer
Description: Data for total labor force (number of people) was obtained from 1980-2012 from World Bank assessments (data.worldbank.org/indicator/SL.TLF.TOTL.IN). The World Bank defines total labor force based on the International Labour Organisation (ILO)'s definition of an economically active population – those 15 years old and older who can supply labor for the production of goods and services – and includes those employed and unemployed, as well as those in the armed forces, and generally excludes homemakers and other unpaid caregivers and workers in the informal sector. For the three EEZs that fall within the China region (China, Macau, and Hong Kong), we combined the values by summing.

5.32. Mangroves

Update: no update possible; values from 2012 Index used. However, data were processed differently: mangrove data now include 1km inland in addition to 1km offshore.

5.33. Mariculture Sustainability Index (MSI): mariculture sustainability and mariculture regulations

Update: no update possible; values from 2012 Index used but with emergent changes.
Description: Since the Index comprises species-specific sustainability values that are combined as a catch-weighted average, where the list of species harvested changed, the sustainability index used in the mariculture sub-goal model also changed due to shifts in the relative composition of harvested species.

5.34. Mariculture yield

Update: additional year(s) available
Description: Updated mariculture yield data were available from 1950-2011 from FAO using FishstatJ 2.0.0 (www.fao.org/fishery/statistics/software/fishstatj/en; accessed in July, 2013). Mariculture was defined as the production of marine taxa from marine and brackish water environments as assigned by FAO, excluding seaweeds from these environments (seaweeds are included in the Natural Products goal). Values were smoothed with a 4-year running mean to help minimize reporting errors, as was done previously (Halpern et al. 2012). If no mariculture yield

was reported in the most recent year (2011), the US dollar value from the previous year (2010) was used to calculate status and trend; this does not penalize a country with recent mariculture yields but lacking a monetary value in 2011, potentially because of reporting issues. This one-year allowance does penalize the country if it has not reported for more than one year previous. For the three EEZs that fall within the China region (China, Macau, and Hong Kong), we combined the values by summing across these EEZs.

5.35. Marine jobs: commercial fishing

Update: no update possible; values from 2012 Index used.

5.36. Marine jobs: mariculture

Update: no update possible; values from 2012 Index used.

5.37. Marine jobs: marine mammal watching

Update: no update possible; values from 2012 Index used.

5.38. Marine jobs: tidal energy

Update: no update possible; values from 2012 Index used.

5.39. Marine jobs: tourism

Update: although additional data are available, we did not have the time or resources to update this data layer.

5.40. Marine protected areas and terrestrial protected areas

Update: additional year(s) available
Description: Data on protected areas through August, 2013 were accessed from the United Nations Environment Programme - World Conservation Monitoring Centre's World Database on Protected Areas (WDPA) through http://www.protectedplanet.net. We used only WDPA polygons (not points) with a status of "designated" (not "proposed"). These polygons were converted to a 1km Mollweide raster by the value of the year in which the park was decreed "designated". For cases in which polygons overlapped, priority was given first to the parks with a designation type of national (over international) and then the earliest year. In the future, we hope to apply buffers to parks spatially assigned as points based on area with extra logic to portion out the parks based on percentage marine vs terrestrial and limited to within country borders where applicable (Visconti et al. 2013). Spatial subsets used within the Lasting Special Places (LSP) sub-goal include the offshore 3 nm and inland 1km. The most recent full year was used for LSP status to be inclusive as possible, but presume a 3-year lag in exhaustive reporting to better estimate a trend.

5.41. Marine revenue: aquarium trade fishing

Update: additional year(s) available

Description: Updated revenue data for aquarium trade fishing (ornamental fish fishing) were available through 2009 from FAO using FishstatJ 2.0.0 (accessed in July, 2013). Subcategory characterization has been updated with the March 2013 release of version 2.0.0 and within these new subcategories two of the three were included: 'ornamental fish nei' and 'ornamental saltwater fish.' As only data from FAO (reported in US dollars) were used, no conversions from local currencies were necessary; the supplemental information in 2012 incorrectly referred to converting from local currencies due to other data sources including Global Marine Aquarium Database (GMAD) (reported in local currencies) that were explored but not included in final models.

5.42. Marine revenue: commercial fishing

Update: no update possible; values from 2012 Index used.

5.43. Marine revenue: mariculture

Update: additional year(s) available
Description: Updated revenue data from mariculture exports were available through 2011 from FAO using FishstatJ 2.0.0 (accessed in July, 2013). With the updated release of version 2.0.0, we were able to filter to include marine and brackish environments while excluding freshwater environments. Data are reported in US dollars.

5.44. Marine revenue: marine mammal watching

Update: no update possible; values from 2012 Index used.

5.45. Marine revenue: marine renewable energy

Update: no update possible; values from 2012 Index used.

5.46. Marine revenue: tourism

Update: additional year(s) available
Description: Updated total contribution to GDP data were available through 2012 from the World Travel and Tourism Council (WTTC). Total contribution includes revenue from sectors directly and indirectly associated with travel and tourism. This is the sole measure used to inform marine revenue from tourism; the supplemental information in 2012 incorrectly identified two other metrics that were not used to calculate the Economies sub-goal.

5.47. Marine species

Update: additional year(s) and data available
Description: We limited data to all species having IUCN habitat system of "marine" based on the 2013.1 release of the IUCN Red List of Threatened Species (IUCN 2013). Updated species extinction risk category data were available, but only 15 species changed extinction risk category. However, 47 previously-assessed species were missing an extinction risk category in 2013, and 3842 new species assessments were added. For population trend, 2 species changed their trend, 7 previously-assessed species were missing trends for 2013, and 1346 species had new reported

trends in 2013. We newly included seabirds using distributions from Birdlife International (BirdLife International and NatureServe 2012). Where neither IUCN nor BirdLife International distributions were available, we again supplemented with AquaMaps (Kaschner et al. 2013) using a 0.4 or higher threshold to convert from a continuous probability of encounter to a binary range map. Table D provides the species count per taxa.

Table D. Species counts per taxa that have been formally assessed by IUCN.

Class	count
ACTINOPTERYGII	3216
ANTHOZOA	842
AVES	839
BIVALVIA	34
CEPHALASPIDOMORPHI	4
CEPHALOPODA	195
CHLOROPHYCEAE	1
CHONDRICHTHYES	1065
CRUSTACEA	256
ECHINOIDEA	1
ENOPLA	1
FLORIDEOPHYCEAE	58
GASTROPODA	806
HOLOTHUROIDEA	369
HYDROZOA	16
INSECTA	1
LILIOPSIDA	78
MAGNOLIOPSIDA	64
MAMMALIA	135
MEROSTOMATA	4
MYXINI	76
PHAEOPHYCEAE	15
POLYCHAETA	2
POLYPODIOPSIDA	3
REPTILIA	89
SARCOPTERYGII	2
ULVOPHYCEAE	1

5.48. Marine wages

Update: additional year(s) available
Description: Updated Occupational Wages around the World (OWW) data were available through 2008 from the National Bureau of Economic Research (www.nber.org/oww/). As in the 2012 assessment, we used the calibration that was recommended by the database creators. The calibration procedures used to normalize the data have been updated in the recent version (w3wl:

wage with country-specific calibration and imputation, lexicographic weighting). We multiplied their calculation of monthly wage in US dollars by 12 to get annual wages for each sector and occupation classification as reported in Table S18 of Halpern et al. (2012). We then calculated the mean annual wage across all marine occupations within the five reporting sectors (coastal fishing, shipbuilding, tourism, transportation, port and harbor services). As these data are all reported in US dollars, no historic currency exchange information was required (as described in the 2012 supplemental information).

5.49. Multispecies maximum sustainable yield (mMSY)

Update: no longer used

5.50. National percent unemployment

Update: additional year(s) available
Description: Updated unemployment data through 2012 were available from the World Bank (http://data.worldbank.org/indicator/SL.UEM.TOTL.ZS). These data are reported as the percent of the total labor force that is available to work and seeking employment but is without work. Because other data layers used for the tourism and recreation goal ended in 2011, we used 2011 values for current status calculations. Gap-filling procedures (see section 6 below) were required for >100 regions per year; in 5-20 cases this produced negative values and in 8-10 cases gap-filling procedures did not have sufficient data to produce a value. For each of these cases we set unemployment to the average per-year value from the regions with reported values (2011 = 8.78%, 2010 = 8.96%, 2009 = 9.34%, 2008 = 8.9%, 2007 = 8.3%, and 2006 8.7%). For the three EEZs that fall within the China region (China, Macau, and Hong Kong), we combined the values using a population-weighted average.

5.51. Natural Products exposure

Update: additional year(s) available
Description: Extent of rocky and coral reef habitat, which in combination with harvest rates are used to estimate this exposure term, were not updated from previous estimates. We were able to update harvest rates (see next data layer), allowing this exposure parameter to reflect those changes.

5.52. Natural Products harvest

Update: additional year(s) available.
Description: Updated data were available through 2010 for all Natural Products categories (sponges, fish oil, seaweed and plants, ornamental fish, corals, shells), which were accessed from FAO using FishstatJ 2.0.0 (www.fao.org/fishery/statistics/software/fishstatj/en). Only export data were used, with commodity types and their subcategories accessed the same way as in the 2012 assessment, although some subcategories are no longer reported (Agar agar in blocks, Laver smoked, Rock laver). The notation ‘nei’ means ‘not elsewhere included.’ See Table E. For a specific product and region (e.g. ornamental fish in Algeria), export revenue was sometimes reported yet tonnes were not. When this occurred, a linear model relating harvest (tonnes) to value (revenue) was developed for this product-region through time, and estimates based on the model were used to gapfill the missing data.

Table E. List of FAO categories included in each natural product group.

Sponges	Natural Sponges nei, Natural Sponges other than raw, Natural Sponges raw
Fish oil	Alaska pollock oil nei, Anchoveta oil, Capelin oil, Clupeoid oils nei, Cod liver oil, Fish body oils nei, Fish liver oils nei, Gadoid liver oils nei, Hake liver oil, Halibuts liver oils, Herring oil, Jack mackerel oil, Menhaden oil, Pilchard oil, Redfish oil, Sardine oil, Shark liver oil, Shark oil, Squid oil
Seaweed and plants	Agar agar in powder, Agar agar in strips, Agar agar nei, Carrageen (*Chondrus crispus*), Green laver, *Hizikia fusiforme* (brown algae), Kelp, Kelp meal, Laver, dry, Laver, nei, Other brown algae (laminaria, eisenia/ecklonia), Other edible seaweeds, Other inedible seaweeds, Other red algae, Other seaweeds and aquatic plants and products thereof, Undaria pinnafitida (brown algae)
Ornamental fish	Ornamental saltwater fish, Ornamental fish nei
Corals	Coral and the like
Shells	Abalone shells, Miscellaneous corals and shells, Mother of pearl shells, Oyster shells, Sea snail shells, Shells nei, Trochus shells

5.53. Natural Products value

Update: additional year(s) available.
Description: Updated data on the value (export revenue) for all products were available through 2010 from FAO using FishstatJ 2.0.0. For a specific product and region (e.g. ornamental fish in Algeria), export revenue was sometimes reported yet tonnes were not. When this occurred, a linear model relating harvest (tonnes) to value (revenue) was developed for this product-region through time, and estimates based on the model were used to gapfill the missing data.

5.54. Natural Products risk

Update: additional year(s) available.
Description: This data layer was previously labeled "Convention on International Trade in Endangered Species of Wild Fauna and Flora (CITES) listed species" but has been renamed here for clarity on how the data were used. Sustainability risk for each of the Natural Products commodities was assessed separately and was included in the sustainability equation when data were available. Risk assessment for corals was based on species identified by CITES; risk for all corals was set as 1 since species in both subclasses and multiple orders of extant corals in class Anthozoa are listed in CITES Appendices II and III (www.cites.org/eng/app/appendices.php). No sponges, algae or marine plants were listed in CITES and thus their risks were set at 0. Shells were also set as 0 since species were not listed individually in the FAO database and only one marine genus (*Tridacnidae* spp.) and one marine species (*Lithophaga lithophaga*) were identified in CITES Appendix II. Risk for ornamental fish was set based on assessments of cyanide or

dynamite fishing by Reefs at Risk Revisited (www.wri.org/publication/reefs-at-risk-revisited) under the assumption that most ornamental fishes are harvested from coral reefs. For fish oil sustainability, the previous data-poor estimate of stock status (Kleisner & Pauly, 2012) was replaced by a weighted geometric mean of estimated B/B_{MSY} that also corresponds to the fisheries sub-goal status (see section 4.1.1). As was true for the previous iteration, it is not possible to identify which of the species fished in the area are used to extract the fish oil. Therefore the estimate is based on all the stocks harvested.

5.55. Nutrient pollution

Update: additional year(s) available
Description: To allow for more direct comparison between assessments from last year (input data from 2003-2006) and this year (input data from 2007-2010, the most recent available), we recalculated this layer using updated data but the same methods as before. In particular, we used FAO data on annual country-level fertilizer use, averaged over the time periods, with missing values filled in by regression between fertilizer and pesticides (see chemical pollution: land-based organic, described above) when possible, and when not possible with agricultural GDP as a proxy (see Halpern et al. 2008 for methods details). These country-level values were then dasymetrically distributed over a country's landscape using global landcover data from the years 2005 (for the 2003-2006 time period) and 2009 (for the 2007-2010 time period), derived from the MODIS satellite at ~500m resolution (following methods described in Halpern et al. 2008 but with updated MODIS data not available previously). These values were then aggregated by ~140,000 global basins (developed for and presented in Halpern et al. 2008), and diffusive plumes were modeled from each basin's pourpoint. The final non-zero plumes (about ~76,000) were aggregated into ~1km Mollweide (wgs84) projection rasters to produce a single plume-aggregated pollution raster. These raw values were then log(x+1) transformed and normalized to 0-1. A simple visual and pixel-count comparison of the agriculture landcover classes shows that, globally, these classes have not changed much during the two time periods (at the 500m resolution of the data). However, global fertilizer consumption has shown a small, but significant increase globally (roughly 4-8% over our time period).

5.56. Ocean acidification

Update: no update possible; values from 2012 Index used.

5.57. Pathogen pollution

Update: additional year(s) available
Description: Updated proportion of population with access to improved sanitation facilities data were available from 1990-2011 from the World Health Organization and United Nations Children's Fund's Joint Monitoring Programme (WHO/UNICEF's JMP: www.wssinfo.org/data-estimates/table/, accessed May 2013). Processing included the function "na.locf" from the "zoo" package in R (Zeileis & Grothendieck 2005). To calculate the pressure score (number of people without access to improved sanitation), we used the most recent proportion available (2011) with current estimates of coastal population (2013), and rescaled this to 110% of this maximum.

5.58. Pesticides trends

Update: additional year(s) available

Description: Updated pesticide consumption data were available through 2010 from FAO's statistical database FAOSTAT (faostat3.fao.org/home/index.html#DOWNLOAD). Data were summed across all pesticide active ingredients and reported in metric tons. The gap-filling and processing was done in the same way as described for the fertilizer trends data (see section 5.19).

5.59. Relative stock biomass

Update: new data layer introduced in 2013
Description: Mean relative proportion of biomass represented by each stock within a given reporting region. These values were based on the global catch data updated through 2011 (see 5.68. spatially-allocated catch data).

5.60. Rocky reef

Update: no update possible; values from 2012 Index used. These data are only used to assess exposure, as a part of the sustainability assessment, in the natural products goal.

5.61. Salt marsh

Update: no update possible; values from 2012 Index used.

5.62. Sea ice

Update: although additional data are available, we did not have the time or resources to update this data layer.

5.63. Sea level rise

Update: new data layer
Description: The sea level rise data are based on Nicholls and Cazenave, 2010, which can be viewed at: www.aviso.oceanobs.com/en/news/ocean-indicators/mean-sea-level.html. The original data (MSL_Map_MERGED_Global_IB_RWT_NoGIA_Adjust.nc) were reprojected into Geographic WGS84, EPSG:4326 at 0.25x0.25 degree resolution (at the equator, 111 km/degree*0.25 degrees/pixel = ~785 km^2 pixels), and then the rate of sea level rise (mm/yr) per pixel was calculated across the time span of the dataset (Oct 1992 through Dec 2012). This produced a minimum value of -33.998 mm/yr and maximum value of 30.1 mm/yr. To produce a value of net change in sea level, these rates were then multiplied by the duration of the time series (20.167 yrs) to produce a net change in sea level, resulting in a minimum value of -685.63 mm and maximum value of 607.01 mm. For comparison to other pressure layers, these values were then rescaled to the largest absolute value, in this case preserving negative values such that the new values ranged -1.0 to 0.885. Note that this global data set does not take into account Glacial Isostatic Adjustment (GIA) of ~-0.3 mm/yr, although this does affect the normalized values (see www.aviso.oceanobs.com/en/news/ocean-indicators/mean-sea-level/processing-corrections.html#c7323). For the purpose of calculating pressures, negative values were then clamped to zero (i.e., no negative pressure), such that only positive sea level rise values mattered.

5.64. Sea surface temperature (SST) anomalies

Update: additional year(s) available
Description: Sea surface temperature (SST) data were obtained from the Coral Reef Temperature Anomaly Database (CoRTAD) (Selig *et al.* 2010), which was produced by the NOAA National Oceanographic Data Center using 4.6 km (nominally 21 km^2 at the equator) Advanced Very High Resolution Radiometer (AVHRR) Pathfinder Version 5.2 SST data (doi:10.7289/ V5WD3XHB)(Casey *et al.* 2010; Casey et al. 2011) from 1982-2010 (www.nodc.noaa.gov/SatelliteData/CoRTAD). Because SST measurements are less reliable where there is persistent ice, we created an ice mask to identify places near the poles that were almost always covered by significant sea ice. The ice mask was generated primarily from the OSI/SAF Global Daily Sea Ice Concentration Reprocessing Data Set (accession.nodc.noaa.gov/0068294), which was regridded and made available in the Pathfinder V5.2 dataset. In Pathfinder, when the OSI/SAF data are unavailable, the sea ice concentrations from the NCDC Daily OI SST data (Reynolds et al. 2007) are included. For each day of the climatological year (1 through 366), we read in the daily sea ice fraction for that day from all of the years and averaged them to create a daily, sea-ice fraction climatology. We then identified grid cells that always contained a sea ice fraction of greater than 0.15 and masked them out of the analysis.

We defined an anomaly as exceeding the standard deviation (SD) of SSTs from the climatology for that location (i.e., grid cell) and week of the year. The climatology was defined as a weekly average for each ~4 km grid cell from 1982-2010 (see Halpern *et al.* 2008 for original methods description). The frequency of anomalies was calculated for each year in the dataset. We then quantified the difference between the number of anomalies in the 5 most recent years (2005-2010) and the 5 oldest years in the dataset (1982-1986). We also recreated the metric of SST change from Halpern *et al.* (2008), which measured the difference in anomaly frequency from 2000-2005 compared to 1985-1990.

There are three main differences between the dataset used previously (Halpern et al. 2008) and the one generated for this analysis. Since the previous publication, the Pathfinder data used to generate the CoRTAD has been updated to use a different and higher resolution reference (Reynolds *et al.* 2007) dataset to help identify bad data (data are removed if they are +5/-2 from the reference dataset). The new version now more accurately resolves strong temperature gradients like those that occur along major boundary currents or near the coast. By adding in new years to the dataset, we also create a new climatology so the standard deviation thresholds will change.

5.65. Seagrass

Update: no update possible; values from 2012 Index used.

5.66. Sector Evenness

Update: no update conducted, but new values created
Description: For reporting regions added in 2013, the values were gap-filled as averages across neighboring areas, rather than using gap-filled values from single sectors. There were very few data updates available, and these were restricted to 1 or 2 sectors. Therefore, we did not use this new information to recalculate sector evenness as we deemed that these new values in combination with older, more dated, values for the other sectors would generate spurious relative proportions.

5.67. Soft-bottom subtidal condition

Update: no update possible; values from 2012 Index used.

5.68. Spatially-allocated catch data

Update: additional year(s) available

Description: This data layer is derived from annual FAO fisheries wild caught statistics, which are reported by FAO major fishing areas rather than EEZs or countries, and represents total annual catch per Index reporting region for 2007-2011. The proportion of catch in each FAO major fishing area that was spatially allocated to each *Sea Around Us* Project reporting region (Watson et al. 2004) for 2006 (as in Halpern et al. 2008, Halpern et al. 2012) was used to spatially allocate the more recent FAO catch data. In other words, all the rules used to spatially allocate the 2006 catch data were presumed to have remained the same.

A key first step for doing this spatial re-allocation of catch data over time is to resolve differences in taxonomic reporting of catch across years and regions. In some cases, FAO taxon names are different because they differ from the most current scientific naming structure used by *Sea Around Us* and found in FishBase (www.FishBase.org), countries have started reporting new taxa to FAO, or coarse taxonomic groupings found in FAO country reports were assigned to finer taxa by the *Sea Around Us* Project based on inference rules developed as part of the spatial allocation process. For all non-matching taxa in FAO, we attempted to rectify the names so that each taxon name in the FAO data was linked to a corresponding official taxonomic name used by *Sea Around Us* in their 2006 spatially allocated data (hereafter SAU_data).

FAO reports catch at one of six different taxonomic levels. The coarsest level taxa (level one) are broad miscellaneous groups (e.g., 'Finfishes' or 'Pelagic fishes'), level two consists of subphyla or subclasses (e.g., 'Crustacea' or 'Elasmobranchii'), level three consists of orders (e.g., 'Teuthida' or 'Rajiformes'), level four consists of families (e.g., 'Serranidae'), level five consists of genera (e.g., '*Thunnus*'), and level six consists of species identified by their scientific binomena (e.g., *'Thunnus obesus'*). We examined (by FAO statistical area) the taxa that could not be matched and for all non-matching taxa at level six, we identified the taxon at the next closest lower taxonomic group. For example, if *Clupea harengus* was found in the FAO data and not in SAU_data, we first looked for the genus *Clupea*, and then the family Clupeidae, and so on. For non-matching taxa at level five, we first looked in SAU_data for species belonging to the genus in question. If we located species, we checked the FAO data to see if catch for those species had been reported. If not, we allocated proportionally the genus level catch in the FAO to the species in SAU_data. This mismatch occurred frequently with the genus '*Thunnus*'. In all FAO regions, relatively large quantities of '*Thunnus*' were reported. Conversely, in SAU_data, there was much higher prevalence of individual '*Thunnus'* species due to previous disaggregation of *Thunnus* genera by the *Sea Around Us* project. For taxa at levels 2-4, we assigned the taxa to the closest lower taxonomic group found in SAU_data. These corrections were performed based on a case-by-case inspection of mismatches, rather than an automated process, as it was not possible to generate broadly applicable rules. All taxa in the FAO data were matched to a SAU taxon.

Once these naming inconsistencies were resolved, we summed the SAU_data from 2006 by species within each FAO major fishing area and matched these catches to the FAO catches in each of the most recent five years. We could then calculate the proportions of each species in the SAU_data within each EEZ in an FAO statistical area. We multiplied these proportions by the recent (2007-2011) FAO catch statistics by FAO major fishing area in order to obtain updated values per taxon per EEZ within which it was caught. The results are commercial fisheries catch time series from 1950 to 2011 at the resolution of the *Sea Around Us* reporting regions (i.e., EEZ or in some cases finer, see Table G). When gapfilling B/B_{MSY}, if a country belonged to an FAO region that improved its taxonomic reporting since SAUP 2006 data, we did not apply the

penalties for under reporting (Eq. S3) and simply used the average of available B/B_{MSY} estimates in those cases.

5.69. Stock exploitation status

Update: new data layer and different method used
Description: Stock exploitation status is a measure of the biomass level of a commercial fisheries population compared to the biomass required to sustain the fishery in the future. Previously this was assessed via an index based on the relative proportion of stocks in each of 5 exploitation categories (i.e., "developing", "fully exploited", "overexploited", "collapsed", and "rebuilding") defined by the FAO (Halpern et al. 2012). The categories are assigned through a method developed by *Sea Around Us* that uses the proportion of current catch compared to the historical peak and the direction of the trend to determine the category (Frœse et al. 2012).

For this current assessment we replaced this approach with estimates of B/B_{MSY}, the ratio of population abundance compared to the abundance required to deliver maximum sustainable yield, obtained from the catch-MSY model (Martell and Frœse, in press, see the Fisheries sub-goal description for Food Provision for more details). Although both approaches use similar empirically-derived relationships on depletion levels, the current model uses more information contained in the annual catch data and produces estimates that take into account how species with different life-history traits have different levels of resilience to fishing exploitation. An important difference from the previous approach is that the output is an index on a continuous scale, and management frameworks differ in how they define what range of B/B_{MSY} constitutes an "overfished" status, or a "depleted" status, etc. For details on how the values are converted to a score on a scale from 0 to 1, see proportion of the Fisheries sub-goal description for Food Provision. Previously stock status was only used as a sustainability coefficient for Fish Oil in the Natural Products goal; it is now an integral part of how the Fisheries sub-goal in Food Provision goal is calculated.

5.70. Targeted harvest

Update: additional year(s) available
Description: Updated counts of marine mammals and sea turtles data were available through 2011 from FAO using FishstatJ 2.0.0 (accessed in July, 2013). Pressures scores were scaled to the maximum harvest across both 2012 and 2013 OHI assessments, which was Japan's harvest in 2010.

5.71. Total population

Update: additional year(s) available
Description: Updated population data were available through 2012 from the World Bank (data.worldbank.org/indicator/SP.POP.TOTL). For the three EEZs that fall within the China region (China, Macau, and Hong Kong), we combined the values by summing. For 59 regions, data were not reported and so we searched Wikipedia for population estimates. Those estimates were for a single year, so to fill missing years we calculated the average per-year change in population across all regions in the World Bank data, and then applied those percent changes to the single year data from Wikipedia.

5.72. Tourist days per stay

Update: no longer used

5.73. Trash pollution

Update: additional year(s) available
Description: Updated International Coastal Cleanup data available for 2012 from the Ocean Conservancy (www.oceanconservancy.org/our-work/marine-debris/check-out-our-latest-trash.html) were used to calculate density of trash (pounds/mile of total beach cleaned, from both land and sea). We rescaled the trash metric per country using a log-linear scale transformation with a maximum as the largest reported metric plus 10 percent. We then computed the status score per oceanic region as 1-x, where x is the weighted average of the per country scores, weighted by the area of 3nmi inland for each coastal country in that region.

5.74. Travel and Tourism Competitiveness Index (TTCI)

Update: additional year(s) available
Description: Updated assessments of the TTCI were accessed from the 2012-2013 Report (WEF 2013, reports.weforum.org/travel-and-tourism-competitiveness-report-2013/). There were 140 economies covered in 2012-2013, up from 139 in 2010-2011. Seychelles, Guinea, Sierra Leone, Yemen and Haiti were added, Suriname was re-added (after being absent in the last edition because of a lack of data). Angola, Libya, Syria, Timor-Leste, and Tunisia were removed because of insufficient or unreliable data.

5.75. Travel and Tourism Direct Contribution to Employment

Update: new data layer
Description: These data were used in the tourism and recreation goal as an indicator of the number of tourists visiting the coast (assuming that employment in the travel and tourism sectors are dynamic and change to reflect increases or decreases in tourism). These data measure employment that is directly linked to the travel and tourism sectors (such as hotels, airlines, airports, travel agents and leisure & recreation services that deal directly with tourists). Data are available for 181 countries from 1988-2012 (www.wttc.org/research/economic-data-search-tool/); here we use data for 2007-2012.

5.76. UV radiation

Update: additional year(s) available
Description: We followed but improved upon methods developed previously (Halpern et al. 2008). We used two satellites to obtain daily Local Noon Erythemal UV Irradiance (mW/m^2) data for the last 17 years: the Earth Probe/TOMS and Aura/OMI satellites. Descriptions of these satellites can be found at disc.sci.gsfc.nasa.gov/data-holdings/PIP/erythemal_uv_irradiance.shtml. The EP/TOMS satellite provides data at 1x1.25 degree resolution from July 1996 through December 2005, spanning 180 degree latitude and 360 degrees longitude. The Aura/OMI satellite provides data at 1x1 degree resolution from September 2004 through present, spanning 180 degrees latitude and 360 degrees longitude. Raster data come in raw hdf4_eos (ep/toms) and he5/hdf5 (omi/aura) formats from NASA. For EP/TOMS data, we converted the hdf4 format to tif files through gdal_translate; for OMI/Aura, we used the NASA OpenDAP server capabilities to download the rasters in NetCDF format, which we then converted to tif files through gdal.

Both satellites record mW/m^2 (valid range: 0-500). However, the two satellites are not well calibrated relative to each other. We plotted daily mean global UV irradiance for the two satellites over the last 17 years. The irradiance values did not sync between the two satellites. Furthermore, both satellites have missing dates (e.g., 11/1996 for TOMS/EP) and calibration issues. In particular, the EP/TOMS satellite has experienced calibration problems since 2002, so trend analysis is not advised after this date (for more details, see gcmd.gsfc.nasa.gov/KeywordSearch/Metadata.do?Portal=amd&KeywordPath=Locations|VERTICAL+LOCATION|STRATOSPHERE&EntryId=GES_DISC_TOMSEPL3dery_V008&MetadataView=Full&MetadataType=0&lbnode=mdlb5). Similarly, the Aura/OMI satellite has some poorly calibrated dates (e.g., the May 2013 solar eclipse; see disc.sci.gsfc.nasa.gov/Aura/data-holdings/OMI/index.shtml). To avoid these issues, we chose a few select dates for comparison of UV values across the time period: 1997/01/01->2001/12/31 from TOMS/EP and 2008/01/01->2012/12/31 from OMI/AURA. Days missing from above periods were: OMI/AURA (Sept. 27-30, 2008) and TOMS/EP (Nov. 16-18, 1997; Dec. 5-12, 1997; Dec. 13-31, 1998; Jan. 1, 1999; and Nov. 18, 2001).

This approach produced two 5-year time periods to compare changes in UV anomalies. Because the satellite values cannot easily be compared to each other, we chose to create two mean baseline UV values against which to look for anomalies. We calculated two separate baseline means and standard deviations for the two 5-year time periods shown above. Daily irradiance values were averaged into 60 monthly mean UV values for each five year period. Mean monthly UV values exceeding the baseline mean plus one standard deviation were labeled as anomalous pixels. Both time periods had up to 19 anomalous values. Mean global number of anomalous pixels for both time periods were: 11.332, std dev: 2.96 (1997-2001 via ep/toms) and 11.287, std dev: 3.06 (2008-2012 via aura/omi). For reference, this produced a 0.4% percent increase in global 5-year mean anomaly count.

5.77. Worldwide Governance Indicators (WGI)

Update: additional year(s) available
Description: Updated Worldwide Governance Indicators (WGI) data were available through 2011 (last updated: 14-Sep-2012: info.worldbank.org/governance/wgi/index.asp) and scores for the six World Governance Indicators were averaged together. The World Bank's update includes revisions for two of the indicators: Rule of Law and Control of Corruption.

6. Data gap filling procedures

Many data layers had sufficient global coverage (i.e., data for most individual coastal nations) to merit inclusion in the calculation of the Index, yet still had gaps (i.e., regions not represented) that needed to be filled to calculate scores for all Index regions. In this current 2013 assessment, many of the data gaps occurred in the new (small) reporting regions (Table A). Data used for calculation of goal trends also required temporal replication over the previous five years (minimum), and this was not always available. In both cases (data with spatial and temporal gaps) we attempted to fill gaps in a manner as simple, transparent and reproducible as possible.

In regions where data were missing, or were considered too outdated to be informative, we adopted gap-filling procedures based on a hierarchical decision tree, presented below.. These rules were only applied to data that were missing but known to exist in relevant Index regions: gap-filling was not done for regions where the data layer

or goal was deemed not applicable. Such cases in which gap-filling was not applied because it was not applicable included: a) goals that entail local human activities (i.e., artisanal opportunities, natural products, tourism & recreation, and coastal livelihoods & economies) in uninhabited regions (as opposed to goals such as biodiversity and sense of place that matter even in uninhabited locations), b) goals capturing extractive uses (i.e., food provision, natural products) in regions that did not report them, under the assumption that such activities occur only where reported, and c) goals based on the presence of certain habitats (i.e., carbon storage, coastal protection, and biodiversity) in regions where the relevant habitats were not present based on available global maps of habitat cover. A particular case of gap-filling decisions for habitat data is for the "exposure" component of the natural products goal. There were regions that reported the harvest of a product, but, according to the habitat maps, the habitat(s) where these products would be harvested were missing in that region. For these mismatches, the habitat map was assumed to have gaps (i.e., the harvest data were assumed to be more reliable than the habitat extent map), and the habitat data were gap-filled using a georegional average (see below) of exposure values. If, however, a product was reported for a region that includes some offshore territories that in 2013 are reported as separate regions, it was assumed the product is harvested only in the regions with documented extent of the relevant habitats (see section below on "special gap-filling rules" for new reporting regions).

The hierarchical decision tree we used includes four different methods to gap-fill missing data within reporting regions: 1) temporal, by using data from previous years; 2) alternate datasets used as proxies, 3) spatial, by using averages from nearby regions, and 4) in particular cases, special rules when no others applied . To fill a time-series gap within a certain region, we preferred using a region's own data from other available years (option 1) wherever possible, based on the assumption that values from a different year within a region provide better information than values from the same year from nearby regions. For this reason, if a region was present in time-series data with years of data missing, temporal gap-filling was always attempted first, with spatial gap-filling used only when data were too outdated, or for regions that were completely absent from the data source. To decide if a region's values were outdated, we established a year prior to which the data could not be used. This "threshold year" was in most cases set to be 10 years prior to the most recent sampled year in the dataset. In some instances, we used a different dataset from the same region as a proxy for the missing values, if it was reasonable to assume there was a good correlation between the two datasets. Using proxy data in this way was preferred over spatial gap-filling because it does not require the assumption that nearby regions are very similar. This applied to cases such as nutrient input trends, chemical pollution trends, natural products monetary value, and livelihoods and economies (see data-layer sections for more details).

1) Temporal gap filling: gaps in time-series data for each reporting region were filled using one of the following three approaches, each applied when appropriate rather than hierarchically:

- *Previous year:* the value from the previous year is used to replace the current year's value. This approach assumes no change in the past 2 years and was

implemented in cases where the current year could have been missing due to a delay in reporting at the time the Index was calculated. This approach was only implemented for the natural products goal (i.e., for harvested tonnage of each product), and for the mariculture subgoal (i.e., for harvested tonnage of each species).

- *Fitted values:* the available data were used to fit a linear model to the time-series and predict missing values. Data within a 10-year window centered on the gap year (i.e., ± 5 years) were used as input in the fitted model. When the missing year was less than 5 years from the most recent year in the data set, the window was shifted to still include 10 years of data even though it was no longer centered upon the missing year. Temporal gap-filling of this kind was done when at least 2 years of data were available.
- *Fitted values for data older than 10 years:* in the cases of livelihoods & economies and the goals based on habitats (i.e., coastal protection, carbon storage and biodiversity), the 10 year rule was relaxed to include older data due to the scarcity of available data. For more details see Halpern *et al*. (2012).

2) Using other data layers as a proxy: For the evaluation of trends in pesticides (proxy for organic pollution), fertilizers (proxy for nutrient pollution), trash pollution, and natural products harvest/monetary value, missing data were filled using a proxy value from a different data layer from the same region. Fertilizer and pesticide consumption, which were used as input values to calculate pressures, were gap-filled by using the linear relationship between existing data for these two layers (when one value existed but not the other). When data to calculate both trends were missing, it was assumed that the relative rate of change would mimic that of population. The same assumption was made for trends in trash. For natural products, harvested tonnes and monetary value used as input values to calculate the status and weight of each product, respectively, were gap-filled by using a linear relationship between these two layers. Within the goal status calculation, the harvest data are rescaled from 0 to 1 by dividing the current value by the historical maximum. When there were insufficient values in the harvest time-series to calculate a relationship between the two, the current monetary value, rescaled to the historical maximum was used to replace the missing harvest value.

3) Spatial gap filling: For some reporting regions (e.g., small regions that are remote territorial holdings of countries) and/or certain data layers, no data exist or they have no data after 2002 (although see exceptions listed above), and thus temporal gap-filling was not an option. In these cases, we typically used spatial gap-filling techniques, following the same rules as in Halpern *et al*. (2012). For spatial gap-filling, we used one of the following three methods, each applied when appropriate rather than hierarchically:

- *Georegional:* in general, we assumed nearby regions (with data) could serve as reasonable proxies for a region missing data, and so we averaged values from geographically nearby regions to fill the gap. We used two levels of spatial aggregation to determine which regions defined 'nearby', derived from United Nations definitions of geopolitical regions (Table G; http://unstats.un.org/unsd/methods/m49/m49regin.htm). The first level aggregates

geographically closer regions (preferred), while the second defines much larger regions, in some cases coinciding with entire continents (used only when no countries within the 'first level' aggregation had data).

- *Sovereign country + georegional*: often data were missing for small remote islands. Several of these are under the governance of distant countries that would not fall within the same georegion. For institutional and socioeconomic data, we assumed that offshore domains would have more in common with their administrative country than with geographically closer regions. In these cases, the values from the administrative country were used to gap-fill when present, otherwise the georegional averages were used as described above.
- *Habitat regions*: for goals using habitat data (i.e., natural products, carbon storage, coastal prediction, and biodiversity), when the habitat extent data indicated that a given habitat was present, but data on its condition was missing, geo-ecological regional averages were used specific to each habitat type (see Halpern et al. 2012, Selig et al. 2013 for descriptions of these regions). Because no habitat data could be updated for this current assessment, we did not need to repeat this method, but its implications for results remain.

4) Special gap filling rules: In a few cases, we adopted a method unique and appropriate to the specific situation.

- *Inhabited Southern Islands*: For a group of small, remote islands found in the Southern oceans (see Table F), data are often missing. Due to their remote location, a spatial gap-filling approach would result in values from very distant regions, that may have no similarities with these islands, being used to gap-fill, thus leading to biased scores. We assume the tourism & recreation and coastal livelihoods & economies goals do not apply to these areas because they are so scarcely inhabited, and these goals therefore drop out. For the artisanal fishing opportunities goal, on the other hand, we assigned a perfect score because we assume that there is need for it and that it is fully satisfied, since legislative or economic constraints on people's access to artisanal fishing are unlikely in these regions. Note that this only applies to the southern islands that are inhabited (Table F); uninhabited Southern Islands get no score as do all other uninhabited regions.
- *New reporting regions*: Some gap-filling was necessary even when the data sources used were identical to those in the 2012 calculation (i.e., no updated data were available) due to the presence of new, better resolved, reporting regions added for this 2013 calculation (see section 3). Most of the new reporting regions were offshore territorial holdings that in 2012 had been aggregated with their administrative country. For spatially explicit data sources this was not an issue, as we simply re-calculated the values using the new regional delimitations (i.e., all calculations based on habitat coverage, including the soft bottom layer, the exposure layers for the natural products status, the relative weights assigned to the pressure and resilience matrices pertinent to habitat-based goals, most of the pressure layers). However, tabular data were not always reported at the scale of the smaller reporting regions added in 2013 (e.g., trash, targeted harvest, artisanal fishing high & low bycatch, habitat destruction of subtidal hard-bottom, etc.). In

these cases, values from 2012 aggregated reporting regions were disaggregated for corresponding 2013 reporting regions using one of three approaches:

a. identical value assigned, for example with some regulatory measures used in resilience measures (the World Governance Indicators (WGI), the Convention on Biological Diversity (CBD), the Global Competitiveness Index (GCI), the Mariculture Sustainability Index (MSI), the Convention on International Trade in Endangered Species of Wild Fauna and Flora (CITES), alien species regulations, and sector evenness); for the status and trend of habitat-based goals (habitat-specific "health" condition and its trend); for artisanal opportunities (access based on regulations reported by Mora *et al.* 2009); for pressures (artisanal fishing with high bycatch, and targeted harvest)
b. weighted by the relative proportions of coastal population among regions (namely: revenue, number of jobs, adjusted workforce size, pressure from artisanal fishing with low bycatch, and from intertidal habitat destruction)
c. weighted by the relative proportions of corresponding EEZ area (e.g., alien species pressure).

Table F. Uninhabited and 'Southern Islands' reporting regions. Note that not all Southern Islands are uninhabited. *Region ids* correspond to those listed in Table G.

Index region id	Index region name	Southern Island	Inhabited
4	Macquarie Island	N	N
34	Bassas da India	N	N
35	Ile Europa	N	N
36	Ile Tromelin	N	N
89	South Georgia and the South Sandwich Islands	Y	Y
90	Prince Edward Islands	Y	Y
91	Crozet Islands	Y	N
92	Amsterdam Island and Saint Paul Island	Y	Y
93	Kerguelen Islands	Y	Y
94	Heard and McDonald Islands	Y	N
105	Bouvet Island	Y	N
107	Clipperton Island	N	N
149	Jarvis Island	N	N
150	Palmyra Atoll	N	N
158	Howland Island and Baker Island	N	N
159	Johnston Atoll	N	N

Gap filling calculated scores: In two cases, gap-filling was applied to calculated values for the pertinent dimension because individually gap-filled layers would produce inaccurate results (details provided in following examples). These exceptions were:

1. the status for tourism & recreation, where number of tourism sector employees and total number of employees would not be comparable if they were derived

using different source data (e.g., the regional average for the population layer may not be commensurate to the tourism employment value); and

2. the calculation of sector evenness in cases where some of data for any of the sectors were gap-filled. The gap-filled values, even if they are derived from countries with much bigger or smaller populations, such that they are not commensurate in absolute terms to the gap-filled country's workforce, may be used when capturing no-net loss of jobs. The rationale for this comparison is that they can still provide a proxy for the rate of change in jobs, but would create potentially incomparable absolute numbers of employees per sector.

Gap filling assessment

Figure S3 summarizes the degree to which goal scores for each region rely on gap-filled versus actual data. The analysis presented here focuses on spatial gap-filling (vs temporal gap-filling or the use of proxy datasets for the same region) because it represents the most common and ubiquitous approach in this 2013 analysis (although not the most preferred approach), and because using information from other regions requires assumptions about similarities among countries that likely differ ecologically, geo-politically and socio-economically, and thus likely entails greater uncertainty than when information from the same reporting region is used to gap-fill (see details in previous section). Figure C presents regions clustered with their finest-scale georegions (r2; see Table G). When possible, the average of available data in these clustered regions was used to gap-fill missing regions.

A full treatment of the relative importance of uncertainties introduced from the full set of gap filling methods would require substantial analyses that is beyond the scope of the work presented here, but is the focus of ongoing research. For each goal in each reporting region we calculated the proportion of gap-filled data layers to total data layers used, and tracked status, resilience, and pressures separately (represented in Fig. C). Current status contributes 75% of the overall goal score (50% through the current status and an additional 25% through its role in assessing the likely future state; see methods in main manuscript), and thus contributes the most uncertainty when gap-filling occurs in the status dimension (trend contributes 12.5% of the score, while pressures and resilience contribute 6.25% each).

7. Supplementary Tables

Table G. Full list of reporting regions used to report country-level Index scores for 2013, *Sea Around Us* project (SAUP) region IDs matching to each Index region, and United Nations georegions for r2 and r1 groups (see Section 6, spatial gap-filling).

Index region ID	Index region name	Index region key	SAU_data region ID	UN geo-regional group r2	UN geo-regional group r1
1	Cocos Islands	CC	166	53	9
2	Christmas Island	CX	162	53	9
3	Norfolk Island	NF	574	53	9

4	Macquarie Island	MQ	37	53	9
5	New Caledonia	NC	540	54	9
6	Vanuatu	VU	548	54	9
7	Solomon Islands	SB	90	54	9
8	Palau	PW	585	57	9
9	Micronesia	FM	583	57	9
10	Nauru	NR	520	57	9
11	Marshall Islands	MH	584	57	9
12	Wake Island	WI	872	57	9
13	Northern Mariana Islands and Guam	MP	316, 580	57	9
14	Taiwan	TW	157	30	142
15	Philippines	PH	608	35	142
16	Australia	AU	36, 38	53	9
17	Papua New Guinea	PG	598	54	9
18	Fiji	FJ	242	54	9
19	Tuvalu	TV	798	61	9
20	South Korea	KR	408	30	142
21	North Korea	KP	410	30	142
24	Cambodia	KH	116	35	142
25	Thailand	TH	764	35	142
26	Andaman and Nicobar	AN	357	35	142
28	Comoro Islands	KM	174	14	2
29	Mayotte	YT	175	14	2
30	Glorioso Islands	GO	NA	14	2
31	Seychelles	SC	690	14	2
32	Réunion	RE	638	14	2
33	Juan de Nova Island	JN	NA	14	2
34	Bassas da India	BI	NA	14	2
35	Ile Europa	EU	NA	14	2
36	Ile Tromelin	TE	252	14	2
37	Mauritius	MU	480	14	2
38	British Indian Ocean Territory	IO	86	34	142
39	Maldives	MV	462	34	142
40	Sri Lanka	LK	144	34	142

41	Mozambique	MZ	251, 508	14	2
42	Madagascar	MG	450	14	2
43	Kenya	KE	404	14	2
44	Somalia	SO	706	14	2
45	Eritrea	ER	111	14	2
46	Djibouti	DJ	262	14	2
47	Yemen	YE	887	145	142
48	Oman	OM	512	145	142
49	Sudan	SD	736	15	2
50	Saudi Arabia	SA	682, 683, 684	145	142
51	Kuwait	KW	414	145	142
52	Bahrain	BH	48	145	142
53	Pakistan	PK	586	34	142
54	United Arab Emirates	AE	784	145	142
55	Azores	AX	622	39	150
56	Cape Verde	CV	132	11	2
57	Madeira	MD	621	39	150
58	Canary Islands	CS	723	39	150
59	Belgium	BE	56	155	150
60	Gibraltar	GI	292	39	150
61	Tunisia	TN	788	15	2
62	Morocco	MA	504	15	2
63	Western Sahara	EH	732	15	2
64	Mauritania	MR	478	11	2
65	Gambia	GM	270	11	2
66	Senegal	SN	686	11	2
67	Libya	LY	434	15	2
68	Malta	MT	470	39	150
69	Latvia	LV	428	154	150
70	Estonia	EE	233	154	150
71	Bulgaria	BG	100	151	150
72	Romania	RO	642	151	150
73	Russia	RU	643, 645, 647, 648, 649, 650, 651	151	150
74	Georgia	GE	268	145	142
75	Ukraine	UA	804	151	150
76	Turkey	TR	792, 793, 794	145	142
77	Syria	SY	760	145	142
78	Lebanon	LB	422	145	142

79	Israel	IL	376	145	142
80	Greece	GR	300	39	150
81	Cyprus	CY	196	145	142
82	Albania	AL	8	39	150
84	Algeria	DZ	12	15	2
85	Ascension	AC	855	154	150
86	Saint Helena	SH	654	154	150
88	Tristan da Cunha	TA	856	154	150
89	South Georgia and the South Sandwich Islands	GS	239	999	999
90	Prince Edward Islands	PI	711	999	999
91	Crozet Islands	CZ	896	999	999
92	Amsterdam Island and Saint Paul Island	AM	895	999	999
93	Kerguelen Islands	KG	897	999	999
94	Heard and McDonald Islands	HM	334	999	999
95	Falkland Islands	FK	238	154	150
96	Sierra Leone	SL	694	11	2
97	Liberia	LR	430	11	2
98	Togo	TG	768	11	2
99	Benin	BJ	204	11	2
100	République du Congo	CG	178	17	2
101	Namibia	NA	516	18	2
102	South Africa	ZA	710	18	2
103	Sao Tome and Principe	ST	678	17	2
104	Equatorial Guinea	GQ	226	17	2
105	Bouvet Island	BV	74	999	999
106	Ghana	GH	288	11	2
107	Clipperton Island	CP	898	13	419
108	Bermuda	BM	60	29	419
110	Bahamas	BS	44	29	419

111	Turks and Caicos Islands	TC	796	29	419
112	Cuba	CU	192	29	419
113	Cayman Islands	KY	136	29	419
114	Haiti	HT	332, 843	29	419
115	Dominican Republic	DO	214	29	419
116	Puerto Rico and Virgin Islands of the United States	PR	630, 850	29	419
117	British Virgin Islands	VG	92	29	419
118	Anguilla	AI	660	29	419
119	Saint Kitts and Nevis	KN	659	29	419
120	Antigua and Barbuda	AG	28	29	419
121	Montserrat	MS	500	29	419
122	Saint Lucia	LC	662	29	419
123	Dominica	DM	212	29	419
124	Barbados	BB	52	29	419
125	Grenada	GD	308	29	419
126	Trinidad and Tobago	TT	780	29	419
127	Saint Vincent and the Grenadines	VC	670	29	419
129	Panama	PA	591	13	419
130	Costa Rica	CR	188	13	419
131	Nicaragua	NI	558	13	419
132	Colombia	CO	170	5	419
133	Honduras	HN	340	13	419
134	El Salvador	SV	222	13	419
135	Mexico	MX	484	13	419
136	Guatemala	GT	320	13	419
137	Ecuador	EC	218, 219	5	419
138	Peru	PE	604	5	419
139	Venezuela	VE	862	5	419
140	Guadeloupe and Martinique	GX	312, 474	29	419
141	Faeroe Islands	FO	234	154	150
143	Iceland	IS	352	154	150
144	Jan Mayen	SJ	579, 744	154	150
145	Greenland	GL	304	154	150

146	Pitcairn	PN	612	61	9
147	French Polynesia	PF	258	61	9
148	Line Group	LG	NA	57	9
149	Jarvis Island	JI	845	57	9
150	Palmyra Atoll	PY	844	57	9
151	American Samoa	AS	16	61	9
152	Samoa	WS	882	61	9
153	Cook Islands	CK	184	61	9
154	Niue	NU	570	61	9
155	Tonga	TO	776	61	9
156	Tokelau	TK	772	61	9
157	Phoenix Group	PX	NA	57	9
158	Howland Island and Baker Island	HB	846	57	9
159	Johnston Atoll	JA	396	57	9
161	Wallis and Futuna	WF	876	61	9
162	New Zealand	NZ	554, 555	53	9
163	United States	US	488, 840, 841, 842, 848, 851, 852	21	19
164	Belize	BZ	84	13	419
166	Jamaica	JM	388	29	419
167	Guyana	GY	328	5	419
168	Suriname	SR	740	5	419
169	French Guiana	GF	254	5	419
171	Brazil	BR	76, 77	5	419
172	Argentina	AR	32	5	419
173	Uruguay	UY	858	5	419
174	Finland	FI	246	154	150
175	Denmark	DK	208	154	150
176	Germany	DE	276	155	150
177	Netherlands	NL	528	155	150
178	Poland	PL	616	151	150
179	France	FR	250	155	150
180	United Kingdom	GB	826, 830	154	150
181	Ireland	IE	372	154	150
182	Spain	ES	724	39	150
183	Portugal	PT	620	39	150

184	Italy	IT	380	39	150
185	Monaco	MC	492	155	150
186	Montenegro	ME	891	39	150
187	Croatia	HR	191	39	150
188	Slovenia	SI	705	39	150
189	Lithuania	LT	440	154	150
190	Qatar	QA	634	145	142
191	Iran	IR	364	34	142
192	Iraq	IQ	368	145	142
193	Guinea Bissau	GW	624	11	2
194	Guinea	GN	324	11	2
195	Ivory Coast	CI	384	11	2
196	Nigeria	NG	566	11	2
197	Cameroon	CM	120	17	2
198	Gabon	GA	266	17	2
199	Democratic Republic of the Congo	CD	180	17	2
200	Angola	AO	24	17	2
202	Tanzania	TZ	834	14	2
203	India	IN	356	34	142
204	Bangladesh	BD	50	34	142
205	Myanmar	MM	104	35	142
206	Malaysia	MY	458, 459, 460, 461, 463	35	142
207	Vietnam	VN	704	35	142
208	Singapore	SG	702	35	142
209	China	CN	156, 344, 446	30	142
210	Japan	JP	390, 392, 393	30	142
212	Kiribati	KI	296	57	9
213	Antarctica	AQ	NA		
214	Egypt	EG	818	15	2
215	Jordan	JO	400	145	142
216	Indonesia	ID	360, 361, 362	35	142
218	Canada	CA	124	21	19
219	Saint Pierre and Miquelon	PM	666	21	19
220	Sint Maarten	SX	532, 533	29	419
221	Northern Saint-Martin	MF	NA	29	419
222	Sweden	SE	752	154	150
223	Norway	NO	578	154	150

224	Chile	CL	152, 153, 154, 155	5	419
227	Jersey	JE	NA	154	150
228	Guernsey	GG	NA	154	150
231	East Timor	TL	626	35	142
232	Bosnia and Herzegovina	BA	70	39	150
237	Oecussi Ambeno	OE	NA	35	142
244	Curaçao	CW	275	29	419
245	Bonaire	BO	275	29	419
247	Brunei	BN	274	35	142
248	Saba	SQ	274	29	419
249	Sint Eustatius	EQ	274	29	419
250	Aruba	AW	275	29	419

Table H. List of models and parameters used for each goal and sub-goal.

(see following pages)

Goal	Type	Global 2012 Status Model Equations	Variables	Global 2013 Status Model Equations (if different)	Variables
Food Provision (x_{FP})	Food Provision (x_{FP}) goal model	$x_{FP} = w \cdot x_{FIS} + (1-w)x_{MAR}$<br>$w = \frac{B_T}{B_T + \sum_1^k Y_k}$	x_{FIS} = fisheries sub-goal score<br>x_{MAR} = mariculture sub-goal score<br>w = weighting factor<br>k = each mariculture species<br>Y_k= most recent non-zero mariculture harvest<br>B_T = wild-caught fishing yield		
	Fisheries (x_{FIS}) sub-goal model	$x_{FIS} = \left(1 - \frac{\delta B_T}{mMSY_R}\right) * T_C$<br>$mMSY_R = 0.75 \cdot mMSY$<br>$\delta B_T = \begin{cases} 0 & if \ \lvert mMSY_R - B_T \rvert < 0.05 \cdot mMSY \\ \lvert mMSY_R - B_T \rvert & if \ \lvert mMSY_R - B_T \rvert < mMSY_R \\ mMSY_R & otherwise \end{cases}$	δB_T = absolute difference between landed biomass and *mMSY*<br>*mMSY* = multi-species maximum sustainable yield<br>T_C = taxonomic reporting quality correction factor<br>B_T = Wild-caught fishing yield	$x_{FIS} = \left(\prod_{g=1}^{6} SS_{i,g}^{\ C_{i,g}}\right)^{\frac{1}{\sum C_{i,g}}}$<br>$SS_{A,g=6} = \begin{cases} B/B_{MSY} & if \ B/B_{MSY} < 0.95 \\ 1 & if \ 0.95 £ B/B_{MSY} £ 1.05 \\ \max\{1 - a(B/B_{MSY} - 1.05), b\} & if \ B/B_{MSY} > 1.05 \end{cases}$<br>$SS_{A,g<6} = \begin{cases} 0 & if \quad g = 1 \\ 0.10 * \min\{SS_{A,g,\forall g=6}\} & if \quad g = 2 \\ 0.25 * \min\{SS_{A,g,\forall g=6}\} & if \quad g = 3 \\ 0.50 * \min\{SS_{A,g,\forall g=6}\} & if \quad g = 4 \\ 0.75 * \min\{SS_{A,g,\forall g=6}\} & if \quad g = 5 \end{cases}$	*i* = Ocean Health Index reporting region<br>*t* = year<br>*A* = FAO major fishing area<br>*g* = level of taxonomic grouping (ISSCAAP)<br>*SS* = Stock status score<br>*C* = total catch<br>B/B_{MSY} = Population biomass/sustainable harvest biomass<br>*α* = underharvest penalty adjustment<br>*β* = minimum stock score
Food Provision (x_{FP})	Mariculture (x_{MAR}) sub-goal model	$x_{MAR} = \log_{10}(Y_C) / Max$<br>$Y_C = \frac{\sum_1^k (Y_k * S_{M,k})}{A_C}$	Y_C = current sustainably harvested total yield<br>k = each mariculture species<br>$S_{M,k}$ = Sustainability score for each species k<br>A_C = area of coastal waters (3nm)<br>Y_k = yield of each species k	$x_{MAR} = Y_M / Y_{ref}$<br>$Y_M = \frac{\sum_1^k (Y_k * S_{M,k})}{P_C}$<br>$Y_{ref} = Max\{Y_M\}$	Y_M = current sustainably harvested total yield<br>Y_{ref} = maximum sustainably harvested total yield<br>k = each mariculture species<br>$S_{M,k}$ = Sustainability score for each species k<br>P_C = Coastal population within 100km
Artisanal Fishing Opp. (x_{AO})	Artisanal Fishing Opportunities (x_{AO}) goal model	$x_{AO} = (1 - D_U) * S_{AO}$<br>$D_U = (1 - PPPpcGDP) * (1 - O_{AO})$	D_U = Unmet demand<br>S_{AO} = Sustainability of fishing methods<br>O_{AO} = access to artisanal-scale fishing<br>*PPPpcGDP* = log-transformed, rescaled purchasing power parity adjusted per capita GDP		

Natural Products (x_{NP})	Natural Products (x_{NP}) goal model	$x_{NP} = \frac{\sum_{p=1}^{N} w_p x_p}{N}$<br>$X_{Pp} = H_p * S_p$<br>$S_p = 1 - \frac{E + R}{N_S}$,<br>$S_{fo,t} = \frac{\sum_{k=1}^{5} N_k * w_k}{\sum_{k=1}^{5} N_k}$	w_p = proportional peak dollar value of each product relative to the total peak dollar value of all products<br>x_p = individual product score<br>N = number of products ever harvested<br>X_{NPp} = Status of each natural product<br>H_p = harvest level for a product relative to its own (buffered) peak reference point<br>S_p = sustainability of product harvest (all but fish oil)<br>E = exposure term<br>R = risk term<br>N_S = 1 or 2 depending on whether or not a viability term is used<br>$S_{fo,t}$ = Sustainability of fish oil harvest<br>N_k = number of species in each k category of exploitation<br>k = category of stock exploitation<br>w = weight assigned to each category of exploitation status		
Carbon Storage (x_{CS})	Carbon Storage (x_{CS}) goal model	$x_{CS} = \sum_{1}^{k} \left(\frac{C_c}{C_r} * \frac{A_k}{A_T} \right)$	C_c = current 'condition' of k habitat<br>C_r = reference 'condition' of k habitat<br>A_k = amount of area each k habitat type covers<br>A_T = total area covered by all habitats assessed		
Coastal Protection (x_{CP})	Coastal Protection (x_{CP}) goal model	$x_{CP} = \sum_{k} \left(a_k \frac{C_{c,k}}{C_{r,k}} \right)$<br>$a_k = \frac{w_k A_k}{\sum_{k} (w_k A_k)}$<br>$w_k = \frac{r_k}{\sum_{k} r_k}$	C_c = current 'condition' of k habitat<br>C_r = reference 'condition' of k habitat<br>A_k = area each k protective habitat covers<br>α_k = weighted area of each habitat k<br>r_k = rank weight of habitat k protective ability		

Coastal Livelihoods and Economies (x_{LE})	Coastal Livelihoods and Economies (x_{LE}) goal model	$x_{LE} = (x_{LIV} + x_{ECO})/2$	x_{LIV} = livelihoods sub-goal score<br>x_{ECO} = economies sub-goal score		
	Livelihoods (x_{LIV}) sub-goal model	$$x_{LIV} = \frac{\left(\frac{\sum_1^k j_{c,k}}{\sum_1^k j_{r,k}} + \frac{\sum_1^k g_{m,k}}{\sum_1^k g_{r,k}} \right)}{2}$$	j = adjusted total number of jobs within each sector<br>g = average PPP-adjusted wages per job within each sector<br>k = marine jobs sectors<br>c = most recent (current) year<br>r = reference year (for j)<br>m = reference location (for g)		
	Economies (x_{ECO}) sub-goal model	$$x_{ECO} = \sum_1^k \frac{e_{c,k}}{e_{r,k}}$$<br>$$e = \sum_1^k \left(R_{D,k} * m_k \right)$$	e = total adjusted revenue generated directly and indirectly from each sector k<br>c = most recent (current) year<br>r = reference year<br>R_D = direct revenue from each sector<br>m_k = multiplier for indirect revenue for each sector		
Tourism and Recreation (x_{TR})	Tourism and Recreation (x_{TR}) goal model	$$x_{TR} = \log \left[\left(\frac{D_t}{V_T} * S_t \right) + 1 \right]$$	D = number of tourist-days<br>t = most recent year<br>V_T = total country population size<br>S = sustainability factor	$$x_{TR} = E_t \cdot S_t$$<br>$$E_t = \frac{E_{WTTC}}{L_t - (L_t \times U_t)}$$	E = tourism employment proportion<br>S = sustainability measure<br>t = current year<br>E_{WTTC} = number of employees in sectors directly relevant to travel and tourism<br>L = total labor force<br>U = percent unemployment
Sense of Place (x_{SP})	Sense of Place (xTR)	$x_{SP} = (x_{ICO} + x_{LSP})/2$	x_{ICO}= iconic species sub-goal score<br>x_{LSP} = lasting special places sub-goal score		
	Iconic Species (x_{ICO})	$$x_{ICO} = \frac{\sum_{i=1}^6 S_i * w_i}{\sum_{i=1}^6 S_i}$$	i = each IUCN threat category<br>S_i = number of assessed species in category i<br>w_i = status weight assigned per threat category		
	Lasting Special Places (x_{LSP})	$$x_{LSP} = \frac{\left(\frac{\%_{CMPA}}{\%_{Ref\_CMPA}} + \frac{\%_{CP}}{\%_{Ref\_CP}} \right)}{2}$$	CMPA = coastal marine protected area<br>CP = coastline protected		

Clean Waters (x_{CW})		$x_{CW} = \sqrt[4]{a * u * l * d}$	*a* = the number of coastal people without access to sanitation rescaled to the global maximum<br>*u* = 1 – (nutrient input)<br>*l* = 1 – (chemical input)<br>*d* = 1 – (marine debris input)		
Biodiversity (x_{BD})		$X_{BD} = (x_{SPP} + x_{HAB})/2$	x_{SPP} = species sub-goal score<br>x_{HAB} = habitat sub-goal score		
	Species (x_{SPP})	$x_{SPP} = \frac{\sum_{k=1}^{M} \left(\frac{\sum_{i=1}^{N} w_i}{N} \right) * A_c}{A_T}$	N_C = number species in a grid cell c<br>*M* = number of grid cells in the assessment region<br>A_c = total area of grid cell c<br>A_T = total area of the assessment region<br>w_i = status weight assigned per threat category for each *i* species		
	Habitats (x_{HAB})	$x_{HAB} = \sum_{i=1}^{k} \left(C_k * \frac{A_k}{A_T} \right)$	$C_{k,c}$ = current condition of habitat k<br>$C_{k,r}$ = reference condition of habitat k<br>A_k = area of habitat k<br>A_T = total area of assessed habitats		

Table I. Full list of data layers used in the 2013 assessment. In the final column, data are indicated as updated, (y), not updated (n), new to the analysis (* [i.e., they did not exist for the 2012 assessment], or removed from the 2013 analyses.

Data Layer	Brief Description	Dimension	Start Year	End Year	Native Resolution	Reference	Updated
Alien species	Number of alien species per marine ecoregion	Pressure	2008	2008	Ecoregion (sensu Spalding et al., 2007)	Molnar et al. 2008	n
Artisanal fishing: high bycatch	Presence of artisanal blast fishing practices	Status, Trend, Pressure	2009	2009	10 km	Burke et al. 2011	n
Artisanal fishing: low bycatch	Presence of artisanal poison fishing practices	Pressure	2009	2009	10 km	Burke et al. 2011	n
Artisanal fishing: management effectiveness and opportunity	Artisanal fishing opportunity for 206 countries	Status, Resilience	2009	2009	National	Mora et al. 2009, Fig. S4	n
Artisanal fishing: need	Gross Domestic Product, per capita, adjusted by Purchasing Power Parity (PPPpcGDP)	Status, Trend	1980	2012	National	Worldwide Governance Indicators (last updated: 14-Sep-2012)	y
Chemical pollution: land-based inorganic	Modeled pollution from urban runoff from impervious surfaces	Status, Pressure	2000	2000	1 km	Halpern et al. 2008, USGS 2000	n
Chemical pollution: land-based organic	Modeled pollution from pesticides	Status, Pressure	1992	2001	1 km	Halpern et al. 2008, FAO 2010	y
Chemical pollution: ocean-based	Modeled pollution from shipping and ports	Status, Pressure	2002	2005	1 km	Halpern et al. 2008	n
Coastal human population	Human population density within 50 km from the shore	Status, Trend, Pressure	1990	2012	2.5 arcmin	CIESIN & CIAT 2005	y

Coastal land and ocean area	Land area within a fixed distance from the shore (50 mi) and ocean area within a fixed distance from the shore (3 nmi and 10km)	All	2008, 2011	2008, 2011	1 km	VLIZ 2012	y
Commercial fishing: high bycatch	Modeled demersal and pelagic high bycatch fishing pressure	Pressure	1999, 2009	2003, 2011	0.5 deg	Halpern et al. 2008	y
Commercial fishing: low bycatch	Modeled demersal and pelagic low bycatch fishing pressure	Pressure	1999, 2009	2003, 2011	0.5 deg	Halpern et al. 2008	y
Convention on Biological Diversity (CBD) signatories	List of 192 countries who signed CBD	Resilience	2011	2011	National	CBD 2011	n
Convention on Biological Diversity (CBD) survey	Answers to questions relating to alien species, habitat, mariculture, tourism, and water	Resilience	2005	2005	National	CBD 2005	n
Convention on International Trade in Endangered Species of Wild Fauna and Flora (CITES) signatories	List of 178 countries who signed CITES	Resilience	2013	2013	National	CITES 2013	y
Coral reefs	Global coral habitat extent and change in condition	Status, Trend	2002, 1980	2009, 2006	0.5 km; 1 km; Sites (points)	Burke et al. 2011, Bruno and Selig 2007, Schutte et al. 2010, Halpern et al. 2008	n
Country land area	Country land area	Status, Trend, Pressure	2008, 2011	2013, 2012	National/ National	CIA 2013/ ESRI 2012	y/ y
Ecological integrity	Status of species biological diversity	Resilience	2013	2013	National (varies)	IUCN 2013	y

Fertilizer trends	Nitrogen fertilizer consumption by agricultural industry	Trend	2002	2010	National	FAOSTAT 2013	y
Fisheries catch data	Global fisheries catch statistics in yield per species	Status, Trend	1980	2012	0.5 deg/ FAO major fishing area	Watson et al., 2004/ FishStatJ 2012	n/y
Fisheries management effectiveness	Management effectiveness of the world's marine fisheries	Resilience	2009	2009	National	Mora et al. 2009	n
Genetic escapes	Mariculture Sustainability Index (MSI): native or introduced indicator	Pressure	1994	2003	National (varies)	Trujillo 2008	n
Global Competitiveness Index (GCI)	Composite measure of 12 aspects of economic competitiveness	Pressure, Resilience	2012	2013	National	World Economic Forum 2012	y
Gross Domestic Product (GDP)	Gross Domestic Product; Adjustment to all revenue data layers to factor out global economic fluctuations, in 2012 $USD	Status, Trend	1960	2012	National	Worldwide Governance Indicators (last updated: 14-Sep-2012)	y
Habitat destruction: intertidal	Population density within 10 km of the shore	Pressure	1990/	2012	2.5 arcmin (varies)	CIESIN 2013, Halpern et al. 2008	y
Habitat destruction: subtidal hard bottom	Presence of blast and poison artisanal fishing practices	Pressure	2009	2009	10 km	Burke et al. 2011	n
Habitat destruction: subtidal soft bottom	Presence of trawling practices in soft bottom habitats	Status, Pressure	1999	2003	0.5 deg	Halpern et al. 2008	n
Human Development Index (HDI)	United Nations development indicators	Status	2012	2012	National	UNDP 2010	n
Iconic species list	WWF Priority and Flagship Species Lists	Status, Trend	2011	2011	Global; National	Halpern et al. 2012, WWF 2013	n
International arrivals	data not used in 2013						

Labor Force	Number of people aged 15 and older who could contribute to the production of goods and services	Staus, Trend	1990	2011	National	Worldwide Governance Indicators (last updated: 14-Sep-2012)	*
Mangroves	Global mangrove habitat extent, from remote sensing and assessments	Status, Trend	2000	2005	1 arcsec; National	Giri et al. 2011, FAO 2007	n
Mariculture Sustainability Index (MSI): mariculture sustainability and mariculture regulations	Mariculture Sustainability Index (MSI): Mariculture regulations include traceability and code of practice indicators. Mariculture sustainability includes fishmeal use, waste treatment, and seed and larvae origin indicators	Resilience, Status	1994	2003	National	Trujillo 2008	n
Mariculture yield	Production of finfish and invertebrates	Status, Trend	1950	2011	National	FAO FishStatJ 2013	y
Marine jobs: commercial fishing	Global Number of Fishers	Status, Trend	1990	2008	National	FAO Sources 2011	n
Marine jobs: mariculture	Global Number of Fishers database, adjusted by proportion of a country's aquaculture production that is focused on marine species	Status, Trend	1993	2008	National	FAO Sources 2011, FishStat 2012	n
Marine jobs: marine mammal watching	Jobs based on number of whale watchers in a country and a regional average number of whale watchers per employee. Includes all marine mammal watching.	Status, Trend	1998	2008	National	IFAW 2009	n

Marine jobs: tidal energy	La Rance (France) and Annapolis (Canada) tidal plants employment data	Status, Trend	2003	2010	Points (sites)	Électricité de France (EDF) 2010, Ruth Thorbourne, pers. comm. 2011	n
Marine jobs: tourism	Total contribution of tourism to employment	Status, Trend	1988	2012	National	World Tourism and Travel Council (WTTC) 2013	n
Marine protected areas and terrestrial protected areas	Relative area of MPAs within EEZ waters or within 3 nmi of shore, and relative area of designated protected areas (CP) within 1 km of shore	Status, Trend, Resilience	2010	2013	National (varies)	Visconti et al 2013, IUCN & UNEP 2013	y
Marine revenue: aquarium trade fishing	Revenue of Aquarium Trade Fishing derived from commodities database	Status, Trend	1984	2009	National	FAO FishStatJ 2013	y
Marine revenue: commercial fishing	Total revenue from commercial marine fishing	Status, Trend	1997	2007	National	FAO FishStatJ 2013	n
Marine revenue: mariculture	Total revenue from mariculture production of marine species	Status, Trend	1977	2011	National	FAO FishStatJ 2013	y
Marine revenue: marine mammal watching	Total revenue from marine mammal watching	Status, Trend	1998	2008	National	O'Connor 2009	n
Marine revenue: tidal energy	Total revenue from marine renewable energy	Status, Trend	1990/ 2001	2010/ 2008	National	UN Energy Statistics Database 2012/ US Energy Information Administration 2011	n/ n

Marine revenue: tourism	Total tourism revenue by country, adjusted by country's relative proportion of coastal area	Status, Trend	1998	2012	National	World Tourism and Travel Council (WTTC) 2013	y
Marine species	IUCN threat category and spatial distribution of marine species	Status, Trend, Resilience	2011	2013	0.5 deg	Birdlife International & NatureServe 2012, IUCN 2013, Kaschner et al. 2013	y
Marine wages	Occupations within commercial fishing, ports and harbors, ship and boat building, tourism, and transportation and shipping	Status, Trend	1989	2008	National	National Bureau of Economic Research 2012	y
Multispecies maximum sustainable yield (mMSY)	data not used in 2013						
National percent unemployment	Percent of the labor force unemployed but able to and looking for work	Status, Trend	1990	2011	National	Worldwide Governance Indicators (last updated: 14-Sep-2012)	y
Natural Products exposure	Intensity of harvest for coral, ornamental fish, sponges, shells, and seaweeds and plants	Status, Trend	1976/ 1950	2009/ 2008	National/ National	FAO FishStatJ 2013/ Halpern et al. 2008	y/ n
Natural Products harvest	Export (in tons) of coral, ornamental fish, fish oil, sponges, shells, and seaweeds and plants	Status, Trend	1976	2009	National	FAO FishStatJ 2013	y
Natural Products value	Export revenue (US dollars)	Status, Trend	1976	2009	National	FAO FishStatJ 2013	y
Natural Products risk	Assigned risk for Natural Products based on references	Status, Trend	2012	2012	National	CITES 2013 / Reefs at Burke et al., 2011	y/ n

Nutrient pollution	Modeled N input from fertilizer use	Status, Pressure	2007	2010	1 km	Halpern et al. 2008, FAOSTAT 2013	y
Ocean acidification	Change in aragonite saturation state (ASS) levels	Pressure	1870/ 2000	2009	1 deg	Halpern et al. 2008	n
Pathogen pollution	Coastal population (within 50 km) density times % population without access to improved sanitation facilities	Status, Trend, Pressure	1990/ 1990	2010/ 2011	5 km/ National	CIESIN 2013/ WHO-UNICEF Joint Monitoring Programme 2013	y/ y
Pesticide trends	Pesticide consumption by agricultural industry	Trend	1990	2010	National	FAOSTAT 2013	y
Relative stock biomass	Biomass by stock for each region	Status, Trend	2007	2011	National	FAO FishStatJ 2013	*
Rocky reef	Global rocky reef habitat extent	Status	2005	2005	2 arcmin; Points	Halpern et al. 2008	n
Salt marsh	Global salt marsh habitat extent	Status, Trend	1975	2007	National	Bridgham 2006, Dahl 2006, EEA Eionet 2008, Environment New Zealand 2007, JNCC 2004	n
Sea ice	Sea ice change in extent, both edge and shoreline metrics	Status, Trend	1979	2010	25 km	Fetterer et al. 2002	n
Sea level rise	Net change in sea level during the time series	Pressure	1992	2012	0.25 deg	AVISO 2013	*
Sea surface temperature (SST) anomalies	Sea surface temperature anomalies	Pressure	1982	2010	4 km	Halpern et al. 2008	y
Seagrass	Global seagrass habitat extent and change in condition	Status, Trend	1975	2010	1 km, National	UNEP-WCMA 2005, Waycott et al. 2009, Short 2011	n

Sector Evenness	Evenness of jobs from Shannon-Weaver diversity index	Resilience	1990	2010	National	FAO Sources 2011, WTTC 2013, O'Connor 2009, Électricité de France (EDF) 2010	n
Soft-bottom subtidal	Global soft-bottom subtidal habitat extent, and modeled status for change in condition	Status, Trend	2001	2005	0.5 deg	Halpern et al. 2008, SAUP	n
Spatialy-allocated catch data	Derived from FAO fisheries catch statistics	Status, Trend	2007	2011	National	FAO FishStatJ 2013, SAUP	y
Stock exploitation status	Exploitation status of fished stocks	Status, Trend	1950	2006	National	FAO FishStatJ 2013, SAUP	n
Targeted harvest	Catch statistics for cetaceans and marine turtles	Pressure	1950	2011	National	FAO FishStatJ 2013	y
Total population	Census populations for 221 countries	Status, Trend	1990	2012	National	Worldwide Governance Indicators (last updated: 14-Sep-2012)	y
Tourist days per stay	data not used in 2013						
Trash pollution	Trash collected on beaches (pounds/mile)	Status, Pressure	2011	2011	National	Ocean Conservancy 2012	y
Travel and Tourism Competitiveness Index (TTCI)	Sustainability of the travel and tourism industry	Status, Trend	2013	2013	National	Blanke & Chiesa, 2013	y
Travel and Tourism Direct Contribution to Employment	Employment directly linked to travel and tourism sectors (hotels, transportation, services)	Status, Trend	1988	2012	National	WTTC 2013	*
UV radiation	Anomalies in intensity of ultraviolet (UV) radiation	Pressure	1996, 2004	2005, 2013	1 deg	NASA 2013	y

Worldwide Governance Indicators (WGI)	Accountability, Political Stability and Absence of Violence, Government Effectiveness, Regulatory Quality, Rule of Law, and Control of Corruption	Pressure, Resilience	1996	2011	National	Worldwide Governance Indicators	y

Table J. Updated pressures matrix. Values come primarily from Halpern et al. (2012). Changes are only for the addition of sea level rise and targeted harvest (used in previous analysis but accidentally omitted from the table).

		ECOLOGICAL														PHYSICAL				SOCIAL
		POLLUTION				Habitat Destruction			Spp Pollution		Fishing pressure					Climate change				Social
**GOAL**	**SUB-GOAL or SUB-COMPONENT**	Chemicals (incl. Oil)	Human Pathogens	Eutrophication & hypoxia	Trash	HD subtidal softbottom	HD subtidal hardbottom	HD intertidal	Alien invasives	Genetic Escapes	Targeted harvest	Commercial High bycatch	Commercial Low bycatch	Artisanal Low bycatch	Artisanal High bycatch	SST	pH	UV	Sea level rise	1-WGI
**FOOD PROVISION**	**Fishing**	1		1		2	2	1	1	1		3	1	1	2					x
	**Mariculture**	2		3															1	x
**ARTISANAL OPPORUNITY**		1		1		1	3	1	1			2	1		3					x
**NATURAL PRODUCTS**	**aq. trade**	2		1			3		1					1	3	1				x
	**coral**	1		2			3		1							3	1	1	1	x
	**fish oil**	2		1		2			1				2			1				x
	**seaweed**	2		2				1	1							1				x
	**sponges**			1		3			1						1	1	1			x
	**Shells**			1		2		1	1							1	1			x
**CARBON STORAGE**	**Mangroves**	1		1				3											1	x
	**Seagrasses**	2		3				3	1							2	1		2	x
	**Salt marshes**	1		2				3	1										2	x
**COASTAL**	**Mangroves**	1		1				3											2	x

PROTECTION	Corals	1		2			3		1							3	1	1	2	x
	Seagrasses	2		3				3	1							2	1		3	x
	Salt marshes	1		2				3	1										3	x
	Sea ice															3			2	x
TOURISM & RECREATION		3	3	3	3														2	x
COASTAL LIVELIHOODS AND ECONOMIES	Fishing (commercial)	2	0	1	0	2	2	1	1	1		3	1	1	2	0	0	0	0	X
	Mariculture	2	0	3	0	0	0	0	0	0		0	0	0	0	0	0	0	1	X
	Tourism	3	3	3	3	0	0	0	0	0		0	0	0	0	0	0	0	2	X
	Shipping & Transport	0	0	0	0	0	0	0	1	0		0	0	0	0	0	0	0	0	X
	Marine cetacean watching	0	0	0	1	0	0	0	0	0		1	0	0	0	0	0	0	0	X
	Aquarium trade	2	0	1	0	0	3	0	1	0		0	0	1	3	1	1	0	0	X
	Ports & Harbors	0	0	0	0	0	0	0	1	0		0	0	0	0	0	0	0	2	X
	Ship & Boat building	0	0	0	0	0	0	0	0	0		0	0	0	0	0	0	0	0	X
	Marine renewable energy	0	0	0	0	0	0	0	0	0		0	0	0	0	0	0	0	1	X
	Livelihoods AVE	0.8	0.3	0.8	0.4	0.22	0.22	0.11	0.3	0.1		0.44	0.11	0.11	0.22	0	0	0	1	x
	Economies AVE	1.3	0.4	1.1	0.6	0.29	0.71	0.14	0.3	0.1		0.57	0.14	0.29	0.71	0.1	0.1	0	1	x
SENSE OF PLACE	Special Places	2		2	3		2	3	1										1	x
	Iconic Species	3		1	1		2	3	1		2	2			2	1	1			x
CLEAN WATERS		3	3	3	3															x

BIODIVERSITY	Habitats - Mangroves	1		1				3											1	x
	Habitats - Seagrasses	2		3				3	1							2	1		2	x
	Habitats - Salt marshes	1		2				3	1										2	x
	Habitats - Soft bottom	2		2		3			1			3	1	1						x
	Habitats - Corals	1		2			3		1						3	3	1	1	1	x
	Habitats - Sea-ice															3			1	x
	Species	2		3	1	3	2	2	1	1	1	3	1	1	2	1	1	1		x

Table K. Resilience matrix. Resilience measures are noted as relevant or not relevant to a goal; the potential effectiveness of the measures is currently not possible to measure globally. No changes were made from previous analyses (Halpern et al. 2012).

		Ecological Resilience												Social Resilience		
		Goal-Specific Regulations										Ecological Integrity				
GOAL	SUB-GOAL	CBD Water	CBD Tourism	CBD Mariculture	CBD Alien Sp.	Habitat Resilience (Coastal)	Habitat Resilience (EEZ)	Fishing Resilience (Coastal)	Fishing Resilience (EEZ)	MSI Regulations	CITES Signatories	Diversity Index (coastal)	Diversity Index (EZZ)	WGI	Sector Diversity	GCI
FOOD PROVISION	Fishing						X		VERSION 2				X	X		
	Mariculture	X		X						X				X		
ARTISANAL OPPORUNITY						X		VERSION 1				X		X		
NATURAL PRODUCTS	Aquar. Trade	X				X		VERSION 3			X	X		X		
	Coral	X				X					X	X		X		
	Fish oil	X				X		VERSION 1			X	X		X		
	Seaweeds	X									X	X		X		
	Sponges					X					X	X		X		
	Shells					X					X	X		X		
CARBON STORAGE	Mangroves					X								X		
	Seagrasses	X				X								X		
	Salt Marshes	X				X								X		
COASTAL PROTECTION	Mangrove					X								X		
	Corals	X				X								X		
	Seagrasses	X				X								X		
	Salt Marshes	X				X								X		
	Sea Ice													X		
TOURISM & RECREATION		X												X		
COASTAL LIVELIHOODS AND ECONOMIES	Livelihoods													X	X	X
	Economies													X		X
SENSE OF PLACE	Special Places	X					ALT VERS							X		
	Iconic Species	X					X		VERSION 2		X		X	X		
CLEAN WATERS		X												X		
BIODIVERSITY	Mangroves		X	X	X		X						X	X		
	Seagrasses	X	X	X	X		X						X	X		
	Salt Marshes	X	X	X	X		X						X	X		
	Soft Bottom	X	X	X	X		X		VERSION 1				X	X		
	Corals	X	X	X	X		X		VERSION 3				X	X		
	Sea Ice		X	X	X								X	X		
	Species	X	X	X	X		X		VERSION 2		X			X		

Table L. Index, goal and sub-goal scores for 2013, globally and for each region.

			Goal/Sub-Goal Scores																	
code	Country/EEZ	Index		FP		AO	NP	CS	CP		LE		TR		SP		CW		BD	
			FIS		MAR					LIV		ECO		ICO		LSP		HAB		SPP
**Global (area-weighted average)**		67	59	58	26	68	46	74	69	77	82	88	45	60	59	59	74	88	85	83
**1**	Cocos Islands	72	34	34		88		93	53	93	96	100	67	66	33	0	92	98	92	86
**2**	Christmas Island	71	31	31		88		93	25	93	96	100	67	50	58	66	92	98	91	85
**3**	Norfolk Island	79	44	44		89				93	96	100	68	89	70	50	90	100	93	86
**4**	Macquarie Island	87	51	51		89									100	100	100	100	94	87
**5**	New Caledonia	69	97	74	16	60	100	50	59	100	94	88	68	62	33	5	76	75	77	79
**6**	Vanuatu	67	92	91	0	45	61	63	31	100	100	100	99	56	29	2	68	84	82	81
**7**	Solomon Islands	62	98	98	0	44	62	49	58	100	100	100	28	54	36	18	67	75	78	81
**8**	Palau	62	96	96	2	51	0	64	54	65	83	100	71	60	30	0	79	98	91	83
**9**	Micronesia	63	74	74	0	45	44	93	73	75	61	48	68	48	24	0	57	98	89	80
**10**	Nauru	69	96	96	0	46			96	75	73	71	69	66	33	0	51	99	89	79
**11**	Marshall Islands	65	96	96		52	22	87	80	76	59	41	69	64	32	0	61	97	89	80
**12**	Wake Island	68	7	7		93			78	79	75	71	71	37	68	100	73	79	81	83
**13**	Northern Mariana Islands and Guam	63	18	18	0	83		94	45	77	63	48	68	57	52	46	59	96	89	81
**14**	Taiwan	55	28	30	33	69	33	96	41	87	92	97	8	56	36	16	57	98	91	85
**15**	Philippines	55	88	75	13	59	53	62	54	43	45	46	28	53	46	39	50	75	77	79
**16**	Australia	77	58	47	14	90	50	91	58	91	96	100	64	73	87	100	93	94	91	87
**17**	Papua New Guinea	59	98	98	0	51	18	78	51	100	100	100	8	55	33	12	71	85	82	78
**18**	Fiji	69	87	87	0	46	36	56	77	52	66	79	97	51	74	96	74	79	78	77
**19**	Tuvalu	70	98	98	0	45			100	100	100	100	30	59	38	17	60	100	89	78
**20**	South Korea	68	2	36	57	83	81			90	95	100	31	73	62	50	68	96	87	78
**21**	North Korea	51	54	50	14	52	21		94	92	70	48	0	68	34	0	51	100	88	75
**24**	Cambodia	54	7	8	10	65	16	52	60	49	75	100	70	43	53	64	62	81	80	78
**25**	Thailand	67	68	84	92	70	67	66	38	72	86	100	54	51	60	69	64	83	82	81
**26**	Andaman and Nicobar	54	16	16		54		73	52	64	78	92	43		23	23	63	90	85	81
**28**	Comoro Islands	56	79	79		43		85	27	93	92	92	18	56	30	4	47	88	83	78
**29**	Mayotte	70	88	86	1	48		93	36	100	100	100	54	48	74	100	47	96	91	85
**30**	Glorioso Islands	65				48			93	93	95	97	55		0	0	74	96	91	85
**31**	Seychelles	74	79	79	0	55	24	100	85	100	84	69	100	60	59	58	64	98	90	83
**32**	Reunion	68	82	80	0	48			92	59	71	84	10	82	80	77	71	96	91	86
**33**	Juan de Nova Island	65				48			93	93	95	97	55		0	0	74	96	91	86
**34**	Bassas da India	66				48			93						0	0	100	96	91	86
**35**	Ile Europa	72				48		100	94						0	0	100	96	91	85
**36**	Ile Tromelin	69	83	83		48			92						0	0	100	93	89	86
**37**	Mauritius	70	69	68	1	53	42	91	81	73	72	71	100	56	43	29	57	95	90	84
**38**	British Indian Ocean Territory	80	83	83		50			100	57	77	97	86	47	73	100	75	100	93	87
**39**	Maldives	61	80	80		48	1	69	63	100	70	41	95	56	28	0	66	91	85	80

Goal/Sub-Goal Scores

code	Country/EEZ	Index		FP		AO	NP	CS	CP		LE		TR		SP		CW		BD	
			FIS		MAR					LIV		ECO		ICO		LSP		HAB		SPP
**40**	Sri Lanka	51	35	32	0	47	42	76	29	49	68	87	30	52	37	23	59	97	89	81
**41**	Mozambique	65	76	75	0	43	56	96	69	100	100	100	25	55	47	38	51	93	87	81
**42**	Madagascar	57	55	53	1	43	92	65	40	63	81	100	12	53	41	29	61	83	80	78
**43**	Kenya	63	63	63	0	59	65	81	58	98	99	100	14	56	56	57	58	82	82	81
**44**	Somalia	48	75	75		42	36	46	35	93	94	95	5	53	27	0	52	63	69	75
**45**	Eritrea	53	68	68		56	63	85	87	0	3	7	5	47	23	0	57	92	84	76
**46**	Djibouti	64	79	79		52			98	93	97	100	5	67	35	4	61	100	90	80
**47**	Yemen	54	69	69		57	47	68	31	100	98	96	14	54	27	0	47	90	84	77
**48**	Oman	68	84	83	0	64	15		99	100	100	100	29	54	49	44	78	98	90	82
**49**	Sudan	53	68	68		50	30	82	30	48	74	99	12	50	25	0	75	95	86	76
**50**	Saudi Arabia	57	72	65	19	68	10	81	29	100	100	100	19	50	38	27	67	94	87	81
**51**	Kuwait	64	39	39	0	90		87	24	100	100	100	20	41	57	73	71	97	89	82
**52**	Bahrain	57	75	75	0	68	0	86	39	88	83	77	48	37	19	0	70	90	85	80
**53**	Pakistan	55	62	62	0	58	100	30	30	69	85	100	17	65	51	37	50	58	68	78
**54**	United Arab Emirates	74	77	77	0	75	58	93	33	100	100	100	59	59	71	82	85	98	91	85
**55**	Azores	67	43	43		69				71	85	100	76	0	43	87	67	95	86	78
**56**	Cape Verde	73	72	72		47				100	100	100	100	68	34	0	67	99	90	81
**57**	Madeira	70	60	60		69				70	83	95	76	0	48	95	65	95	88	81
**58**	Canary Islands	64	73	73		69		54	54	71	60	49	76	0	48	97	64	76	79	83
**59**	Belgium	76	64	64		77	54	100	100	64	82	100	32	51	75	100	82	99	93	87
**60**	Gibraltar	74	84	84		69				100	76	51	77	52	76	100	48	100	89	78
**61**	Tunisia	64	58	54	2	69	55			67	84	100	54	64	33	1	73	97	87	78
**62**	Morocco	68	79	79	0	60	34			100	100	100	82	56	40	24	63	90	84	78
**63**	Western Sahara	68	73	73		47				94	97	100	37	58	56	54	79	99	87	75
**64**	Mauritania	62	65	65		52	43			96	94	91	3	61	80	100	73	97	90	83
**65**	Gambia	67	67	67	0	58		61	61	100	100	100	56	74	57	40	66	76	77	78
**66**	Senegal	50	72	72	0	52	4	33	5	100	100	99	20	59	79	100	66	51	66	80
**67**	Libya	48	61	59	0	50	5			36	67	98	9	47	23	0	86	100	87	75
**68**	Malta	81	78	65	17	72		90	90	78	89	100	100	49	63	78	73	94	87	80
**69**	Latvia	75	76	76		68	61	54	100	81	91	100	36		100	100	71	87	91	94
**70**	Estonia	78	75	75	0	77	29	100	100	51	76	100	46		100	100	84	100	97	95
**71**	Bulgaria	73	89	89	5	65	83			43	72	100	37	53	76	100	71	100	92	85
**72**	Romania	60	61	61		66	12			80	90	100	20	53	77	100	60	100	94	87
**73**	Russia	66	31	31	3	73	3	100	94	86	93	100	13	72	82	92	74	100	93	86
**74**	Georgia	63	95	95		69	10			99	100	100	30	52	47	41	65	100	93	86
**75**	Ukraine	68	91	91	0	61	56	84	84	60	80	100	17	52	59	66	61	94	89	84
**76**	Turkey	58	65	56	10	72	30			100	100	100	24	48	31	13	64	89	87	85
**77**	Syria	67	50	50		75	82			100	100	100	68	46	23	0	51	96	91	86
**78**	Lebanon	66	47	47		63	100			66	83	100	69	45	23	0	53	90	88	87
**79**	Israel	68	52	35	1	77	45	91	91	100	100	100	30	42	39	36	76	95	92	89
**80**	Greece	71	64	53	32	68	53			62	64	66	96	60	71	83	75	96	91	85

Goal/Sub-Goal Scores

code	Country/EEZ	Index		FP		AO	NP	CS	CP		LE		TR		SP		CW		BD	
			FIS		MAR					LIV		ECO		ICO		LSP		HAB		SPP
**81**	Cyprus	56	47	31	14	78	0			64	67	69	61	47	44	41	77	97	93	90
**82**	Albania	57	62	41	4	69	4			100	100	100	41	47	54	60	57	100	90	80
**84**	Algeria	63	77	77	0	68				100	100	100	20	48	25	3	68	89	83	76
**85**	Ascension	64	45	45		87				86	87	88	48		0	0	87	99	91	83
**86**	Saint Helena	70	67	67		87				61	43	25	48	69	71	72	86	99	92	85
**88**	Tristan da Cunha	62	34	34		87				86	87	88	48		0	0	87	99	89	79
**89**	South Georgia and the South Sandwich Islands	77	28	28										77	88	100	100	95	92	89
**90**	Prince Edward Islands	94	85	85											100	100	100	99	89	80
**91**	Crozet Islands	86	86	86										52	76	100	100		83	83
**92**	Amsterdam Island and Saint Paul Island	70	13	13										52	76	100	100	100	91	81
**93**	Kerguelen Islands	87	79	79										52	76	100	100	100	93	85
**94**	Heard and McDonald Islands	93	78	78											100	100	100	100	94	87
**95**	Falkland Islands	61	40	40	0	87				61	39	18	48	76	38	0	87	99	91	83
**96**	Sierra Leone	45	76	76		58		23	6	54	77	99	8	54	31	8	59	51	65	79
**97**	Liberia	47	53	53		42		23	23	100	100	100	27	51	25	0	62	58	69	80
**98**	Togo	67	82	82		42		99	99	100	100	100	6	59	29	0	55	100	88	77
**99**	Benin	61	59	59		43		100	100	34	67	100	8	61	30	0	49	100	90	80
**100**	Republique du Congo	54	72	72		51		28		57	77	97	5	61	73	85	59	55	67	80
**101**	Namibia	61	35	35	16	86	17			100	76	52	24	58	79	100	80	97	89	80
**102**	South Africa	63	49	49	1	72	35	81	23	100	100	100	43	60	78	95	69	90	85	80
**103**	Sao Tome and Principe	60	54	54		44				100	100	100	47	52	26	0	56	100	90	80
**104**	Equatorial Guinea	57	50	50		58		85	84	7	13	19	13	56	68	79	60	93	84	76
**105**	Bouvet Island	54	30	30											0	0	100		88	88
**106**	Ghana	51	79	79		45	29	69	18	89	95	100	11	59	36	12	55	76	78	80
**107**	Clipperton Island	70	77	77		84									0	0	100	100	90	80
**108**	Bermuda	67	18	18		83			55	65	60	55	100	67	82	97	60	83	81	79
**110**	Bahamas	65	81	81	0	75	21	68	36	70	83	96	100	58	31	3	75	77	83	88
**111**	Turks and Caicos Islands	67	53	53	0	72	12	80	35	94	95	95	100	61	69	76	71	87	87	88
**112**	Cuba	57	20	17	2	78	11	100	31	97	92	88	22	54	70	86	69	80	83	87
**113**	Cayman Islands	68	8	8		71		92	56	100	100	100	51	60	70	80	74	87	88	90
**114**	Haiti	45	1	1		49	69	61	21	100	97	94	5	52	27	3	46	66	74	82
**115**	Dominican Republic	61	19	17	0	63	76	58	55	66	82	99	39	56	78	100	60	81	83	85
**116**	Puerto Rico and Virgin Islands of the United States	62	27	27	0	78		100	37	54	73	92	23	57	62	66	68	95	90	85
**117**	British Virgin Islands	64	10	10		71		74	48	68	75	82	100	59	39	19	74	82	84	86
**118**	Anguilla	61	3	3		71		74	39	38	62	86	100	76	38	0	73	82	84	86
**119**	Saint Kitts and Nevis	57	12	12	0	74		100	45	35	59	82	31	57	29	0	67	95	93	90

			Goal/Sub-Goal Scores																	
code	Country/EEZ	Index	FP			AO	NP	CS	CP	LE			TR	SP			CW	BD		
			FIS		MAR					LIV		ECO		ICO		LSP		HAB		SPP
**120**	Antigua and Barbuda	65	20	20		68		100	28	100	94	88	62	62	59	55	67	87	86	84
**121**	Montserrat	67	1	1		71		74	73	96	91	86	100	59	30	0	69	93	93	93
**122**	Saint Lucia	51	11	11		72	0	27	68	48	60	73	100	61	32	3	60	70	81	91
**123**	Dominica	49	11	11		71		27	7	77	84	91	55	68	36	5	75	60	73	86
**124**	Barbados	50	36	36		60	0	27	28	72	73	74	100	58	31	4	66	82	83	84
**125**	Grenada	50	31	31		56		38	38	100	85	71	27	56	28	0	72	57	72	88
**126**	Trinidad and Tobago	74	25	25		69	95	86	80	88	85	81	99	54	47	41	66	90	87	84
**127**	Saint Vincent and the Grenadines	44	5	5		57	0		73	32	27	22	46	63	32	0	65	92	91	90
**129**	Panama	55	94	92	6	60	4	25	30	100	100	100	53	56	56	55	53	63	75	87
**130**	Costa Rica	58	58	50	6	58	20	48	64	51	76	100	50	57	63	69	71	85	84	83
**131**	Nicaragua	45	54	35	15	52	18	6	9	59	79	100	38	53	75	96	62	60	73	85
**132**	Colombia	55	28	22	4	60	56	50	61	59	80	100	20	54	58	62	71	60	71	83
**133**	Honduras	52	36	14	8	52	6	61	40	88	94	100	43	55	64	72	61	80	82	85
**134**	El Salvador	53	49	47	0	70	24	44	44	42	71	100	23	53	77	100	50	71	77	84
**135**	Mexico	62	50	47	19	58	54	64	35	45	73	100	70	54	77	100	63	82	82	82
**136**	Guatemala	66	86	65	33	54	10	89	89	100	100	100	24	59	78	98	63	96	88	80
**137**	Ecuador	64	45	73	100	62	61	53	57	100	100	100	14	56	78	100	59	77	78	78
**138**	Peru	52	74	73	27	70	35	39	39	80	63	45	20	60	60	60	54	65	71	77
**139**	Venezuela	51	44	39	3	69	0	68	39	71	83	96	21	51	67	83	44	77	78	80
**140**	Guadeloupe and Martinique	52	4	4	0	75	0	100	33	98	65	32	11	58	75	92	68	96	91	85
**141**	Faeroe Islands	64	66	74	100	87	7			86	88	91	48	65	32	0	86	95	89	84
**143**	Iceland	66	59	59	63	75	53		54	85	64	43	62	63	59	56	84	73	79	85
**144**	Jan Mayen	69	64	64		82				86	88	90	49	93	47	0	85	52	71	89
**145**	Greenland	83	68	68		88			97	100	100	100	48	73	86	100	89	94	93	91
**146**	Pitcairn	69	53	53		70			100	89	81	73	33	69	34	0	85	100	92	83
**147**	French Polynesia	72	60	60	50	63	99	92	90	100	73	46	33	64	32	0	85	98	90	83
**148**	Line Group	65				55			100	78	72	66	70		0	0	72	96	86	76
**149**	Jarvis Island	87	53	53		93									100	100	100	93	87	81
**150**	Palmyra Atoll	81	52	52		93			78					56	78	100	100	94	88	82
**151**	American Samoa	64	56	56		83		94	29	99	85	71	30	60	53	46	54	98	88	79
**152**	Samoa	57	51	51	0	71		89	33	3	42	80	32	62	32	1	78	96	89	82
**153**	Cook Islands	56	47	47	0	74	35	51	85	34	45	56	31	62	32	2	76	80	78	77
**154**	Niue	60	58	58		74		51	80	80	56	32	32	61	31	0	83	80	78	76
**155**	Tonga	54	67	67	0	68	0	67	29	100	100	100	30	63	35	7	63	87	83	79
**156**	Tokelau	66	58	58		80		56	94	89	80	72	34	57	28	0	81	78	81	85
**157**	Phoenix Group	80				56			100	78	72	66	71		100	100	75	96	88	80
**158**	Howland Island and Baker Island	95	92	92		86			100						100	100	100	97	90	83
**159**	Johnston Atoll	82	53	53		93			78					56	78	100	100	94	89	84

Goal/Sub-Goal Scores

code	Country/EEZ	Index		FP		AO	NP	CS	CP		LE		TR		SP		CW		BD	
			FIS		MAR					LIV		ECO		ICO		LSP		HAB		SPP
**161**	Wallis and Futuna	72	96	96		63			100	89	81	72	32	76	38	0	74	100	92	84
**162**	New Zealand	79	49	61	100	87	75	82	66	100	100	100	71	62	70	78	91	85	85	84
**163**	United States	69	53	51	11	85	41	67	80	55	78	100	52	63	81	100	79	74	80	85
**164**	Belize	64	34	45	50	70	50	40	10	100	100	100	99	51	74	98	71	61	75	89
**166**	Jamaica	54	3	3	0	55	15	68	27	69	78	87	76	56	78	100	63	71	77	84
**167**	Guyana	53	3	3	1	53		71	71	40	69	98	31	52	26	1	75	87	82	78
**168**	Suriname	65	7	7	0	65		100	100	96	85	74	9	53	76	100	56	100	89	78
**169**	French Guiana	65	16	16		60		100	100	62	47	32	26	60	79	98	69	100	91	82
**171**	Brazil	65	65	55	4	62	14	94	86	60	80	100	31	55	78	100	68	93	87	82
**172**	Argentina	63	43	43	0	70	56			82	91	100	34	62	53	44	67	97	86	75
**173**	Uruguay	59	59	59	0	74	1			100	100	100	36	62	51	41	68	96	85	74
**174**	Finland	76	70	63	15	78	49		100	70	85	100	31		99	99	78	100	98	97
**175**	Denmark	77	64	64	10	89	48	100	94	71	72	73	38	78	89	100	81	97	93	90
**176**	Germany	74	64	34	13	78	70	100	96	83	92	100	29	37	69	100	81	95	92	88
**177**	Netherlands	76	62	54	30	78	49	100	100	55	68	82	60	57	78	100	86	84	85	87
**178**	Poland	65	76	76		76	33	69	49	65	83	100	22		100	100	63	70	82	94
**179**	France	73	67	69	73	81	55	81	39	64	82	100	66	59	79	100	93	87	85	84
**180**	United Kingdom	73	70	64	16	80	69	56	44	84	90	96	84	63	81	100	82	82	83	83
**181**	Ireland	70	75	74	68	78	79	56	56	59	79	100	30	60	80	100	91	80	82	84
**182**	Spain	67	56	59	66	72	50	54	54	57	79	100	72	58	79	100	77	75	79	82
**183**	Portugal	70	57	55	4	70	22			87	93	100	90	56	78	100	62	95	88	82
**184**	Italy	71	65	47	20	72	93	81	36	73	86	100	62	54	77	100	67	93	87	81
**185**	Monaco	72	73	73		78				72	85	99	44	43	57	72	81		86	86
**186**	Montenegro	52	69	66	6	64	0			61	79	98	22	55	28	0	70	99	88	77
**187**	Croatia	72	68	65	16	53	67			60	80	100	98	57	57	56	64	99	89	80
**188**	Slovenia	70	87	72	6	71	42			100	100	100	41	61	80	100	64	99	90	82
**189**	Lithuania	72	79	79		76	51		74	55	78	100	21		100	100	75	92	92	93
**190**	Qatar	64	80	80	0	100		90	34	57	70	84	16	48	24	0	71	98	91	85
**191**	Iran	60	58	57	7	69	100	56	17	100	80	59	14	54	59	64	75	75	77	79
**192**	Iraq	59	61	61		55		82	82	100	86	72	20	59	31	4	33	91	83	75
**193**	Guinea Bissau	48	57	57		50		25	6	96	61	26	26	53	77	100	64	58	67	76
**194**	Guinea	49	64	64		42		33	8	62	80	97	5	53	72	91	70	68	72	75
**195**	Ivory Coast	45	82	82		43	5	22	22	71	85	99	12	58	53	48	61	56	67	78
**196**	Nigeria	56	45	45	0	50	95	97	27	100	76	52	12	63	43	22	50	66	70	73
**197**	Cameroon	60	53	53		43		80	80	55	77	100	10	64	58	51	60	84	79	75
**198**	Gabon	54	62	62		64		48	47	39	59	80	6	66	77	87	62	56	66	75
**199**	Democratic Republic of the Congo	45	30	30		49		20	20	100	98	96	4	48	72	96	44	54	67	80
**200**	Angola	41	16	16		50	1	57	18	61	76	91	6	52	45	38	64	71	73	75
**202**	Tanzania	65	63	62	0	67	72	62	64	100	100	100	15	58	79	100	51	74	77	81
**203**	India	59	50	45	2	45	83	73	53	39	69	100	49	50	39	28	47	89	86	83

		Goal/Sub-Goal Scores																			
code	Country/EEZ	Index		FP		AO	NP	CS	CP		LE		TR		SP		CW		BD	
			FIS		MAR					LIV		ECO		ICO		LSP		HAB		SPP
**204**	Bangladesh	68	2	5	13	58	94	100	100	100	100	100	13	61	59	57	56	100	91	82
**205**	Myanmar	55	2	2	8	51	100	74	46	100	96	92	10	49	29	10	61	88	82	75
**206**	Malaysia	68	65	61	32	66	69	65	76	87	80	72	82	50	34	17	66	82	80	79
**207**	Vietnam	57	3	18	33	51	72	42	66	61	74	86	33	54	77	100	59	79	79	79
**208**	Singapore	63	79	78	2	84	12	91	27	93	96	100	65	54	29	3	70	88	82	77
**209**	China	62	38	75	100	77	71	54	46	93	97	100	41	53	35	17	48	77	79	81
**210**	Japan	67	29	28	25	62	42	96	97	56	78	100	25	59	77	96	76	96	90	84
**212**	Kiribati	71	70	70	0	53	86	87	82	79	90	100	69	62	31	0	55	93	86	79
**213**	Antarctica																			
**214**	Egypt	67	50	24	21	69	51	86	54	96	98	100	52	48	70	92	73	98	90	83
**215**	Jordan	58	5	5		56		88	56	76	88	100	53	52	26	0	68	88	83	79
**216**	Indonesia	62	84	75	16	47	84	50	59	43	71	99	26	55	62	68	66	74	76	79
**218**	Canada	68	60	65	93	83	44	55	96	80	90	100	25	72	50	28	76	93	91	90
**219**	Saint Pierre and Miquelon	70	71	71		78				100	82	64	40	76	48	19	82	100	91	83
**220**	Sint Maarten	59	6	6		61	18	100	32	96	90	84	100	58	30	1	54	100	94	89
**221**	Northern Saint-Martin	80				75		100	35	96	91	86	100		84	84	60	96	93	91
**222**	Sweden	76	72	71	3	73	17	69	100	100	100	100	57	73	86	100	90	94	94	95
**223**	Norway	77	67	77	100	90	37		79	85	82	79	66	62	79	96	91	93	91	90
**224**	Chile	69	75	81	100	77	12			100	100	100	32	65	82	100	81	90	86	82
**227**	Jersey	65				81		55	55	86	87	89	47		39	39	72	79	82	84
**228**	Guernsey	60				80		55	55	86	87	89	47		0	0	73	79	81	83
**231**	East Timor	48	44	44	0	50		56	17	55	27	0	41	59	58	58	64	73	74	76
**232**	Bosnia and Herzegovina	60	64	52	2	61				51	75	100	27	51	75	98	52	72	74	77
**237**	Oecussi Ambeno	53				58		28	52	63	77	90	41		36	36	59	71	73	75
**244**	Curacao	69				61		75	100	65	72	79	48	59	41	23	64	92	89	87
**245**	Bonaire	70			0	61	18	80	94	65	72	80	100	59	46	33	69	94	91	89
**247**	Brunei	64	70	28	4	86		66	99	82	80	77	26	54	36	17	73	90	86	81
**248**	Saba	76				62	19	100	100	65	72	79	100	59	71	82	64	100	95	90
**249**	Sint Eustatius	70				61	18	100	94	65	72	79	100	59	29	0	62	100	95	89
**250**	Aruba	71	3	3		62		86	82	100	100	100	100	62	31	0	86	95	91	88

Table M. Annual difference in scores between 2013 and 2012 for the Index, goals and sub-goals globally and for each region.

Goal/Sub-Goal Scores

	Country/EEZ	Index	FP			AO	NP	CS	CP	LE			TR	SP			CW	BD		
			FIS		MAR					LIV		ECO		ICO		LSP		HAB		SPP
**Global (area-weighted average)**		0.9	0.3	0.6	0.2	0.4	-1.2	0	-0.1	2.1	4.3	6.5	0	0	0.4	0.8	4	0.1	0	-0.1
**1**	Cocos Islands	0	0	0		0.5		0	0	3.8	2	0.3	-0.2	0	0	0	-2.1	0	-0.2	-0.4
**2**	Christmas Island	0	0	0		0.5		0	0	3.8	2	0.3	-0.3	-0.1	-0.2	-0.2	-2	0	-0.2	-0.4
**3**	Norfolk Island	0.2	0.6	0.6		1.7				3.8	2	0.3	-0.3	0.7	0.3	-0.1	-3.4	0	0.4	0.7
**4**	Macquarie Island	1	4.5	4.5		0.5									0	0	0	0	0	0.1
**5**	New Caledonia	-0.2	1	2.6	-2.3	0.1	0.3	0	-0.1	0	1.4	2.8	-0.4	-0.1	-0.1	0	-6.2	0	-0.1	-0.2
**6**	Vanuatu	0.5	0	0	0.1	0	12.4	-0.1	0	0	0	0	-0.7	-0.1	0	0	-5.6	-0.1	-0.2	-0.4
**7**	Solomon Islands	0.7	0.5	0.5	0	0.2	12.2	0.1	0.1	0	0	0	-0.1	0	-0.2	-0.5	-5.3	0.1	0	0
**8**	Palau	-4.6	0.5	1	-1	0.8	-55	-0.1	0	9.5	8.7	7.9	0	0	0	0	-0.7	0	-0.2	-0.3
**9**	Micronesia	3.9	1	1	0	0	35.3	0	0	3.7	3.1	2.5	-0.1	-0.1	0	0	-0.2	0	-0.2	-0.5
**10**	Nauru	0.9	0.5	0.5	0	0.2			0	1.7	7.3	13	-0.1	-0.2	-0.1	0	-0.5	0	-0.2	-0.5
**11**	Marshall Islands	-0.5	0.5	0.5		0.2	-8.9	0	0.1	3.2	2.8	2.6	0	0	0.1	0	-0.3	0	-0.1	-0.2
**12**	Wake Island	0.6	0	0		0			0	2.5	6	9.5	0	0	0	0	-0.7	0	-0.2	-0.4
**13**	Northern Mariana Islands and Guam	1	0	0	-0.1	0.2		0.1	0	-5.4	9.1	23.7	0.2	0.1	-0.1	-0.3	-0.3	0	-0.2	-0.3
**14**	Taiwan	-0.6	0.1	0.2	1.2	0.7	-4.9	0	-0.1	0	2.2	4.3	0	0	0	0	-4.8	0	-0.1	-0.1
**15**	Philippines	3.4	-2.8	-3.4	0	0.3	11.4	0.2	0.1	-8.5	-1.6	5.2	0.1	0.2	0.2	0.2	27	0.2	0.1	0.1
**16**	Australia	1	0.1	1.2	0.2	0.5	6.3	0	0	4.5	2.3	0	0.1	0.1	0.1	0	0.1	0	0	-0.1
**17**	Papua New Guinea	-1.1	1	1	0	0.1	-8.1	0	0	0	2.7	5.4	-0.1	0	0	0	-6.5	0	-0.1	-0.2
**18**	Fiji	-0.2	-1.9	-1.8	0	0.4	-2.6	0.3	0.3	0.5	6.1	11.5	-0.2	0.2	-0.2	-0.5	-4.6	0.3	0.3	0.1
**19**	Tuvalu	3.2	0.5	0.6	-0.1	-0.2			0	0	35.3	70.6	-0.4	-0.6	-0.5	-0.3	-8.9	0	-0.5	-1.1
**20**	South Korea	2.3	0	0.9	0.8	0.5	13.1			2.2	1.1	0	0	0	0	0.1	2.7	0	0	0.1
**21**	North Korea	-1.3	1	1	-0.1	0.7	-9.5		-0.6	2.4	-2.4	-7.2	0	0	0	0	-0.4	0	0	0
**24**	Cambodia	0.5	0.1	0.3	0.2	0.4	11.7	0.1	0.1	-0.6	0.8	2.2	0.1	0	-0.2	-0.3	-8.6	0.1	0.2	0.3
**25**	Thailand	0.7	-1.8	-5.5	-7.8	0.8	-1.3	0.1	0.1	25.3	12.7	0	0.1	0.2	0	0	-0.6	0.2	0.3	0.3
**26**	Andaman and Nicobar	1.6	0	0		0.6		-0.2	-0.2	-2.4	6.1	14.6	-0.1		0.1	0.1	8.5	-0.2	-0.2	-0.3
**28**	Comoro Islands	-0.5	1.1	1.1		0.1		0.1	0	-3.5	0.5	4.4	0	-0.1	-0.1	0	-6	0.1	-0.1	-0.3
**29**	Mayotte	-0.9	1	1.3	-0.1	0.2		0	-0.1	0	0	0	-0.1	-0.2	-0.2	0	-8.5	0	-0.3	-0.6
**30**	Glorioso Islands	-3.1				0.2			0	-2	-1	0	-0.9		0	0	-19.5	0	-0.3	-0.5
**31**	Seychelles	-2	2.1	2.1	0	0.9	-3	0	-0.2	0	3	6	0	-0.3	-0.6	-0.9	-22	0	-0.3	-0.5
**32**	Reunion	-1.5	2	2.8	0	0.2			-0.1	-1.3	4.5	10.3	-0.1	-0.2	-4.5	-8.7	-14.3	0	-0.3	-0.6
**33**	Juan de Nova Island	-3.2				0.1			-0.1	-2	-1	0	-0.9		0	0	-19.6	0	-0.3	-0.6
**34**	Bassas da India	0				0.2			-0.3						0	0	0	0	-0.2	-0.5
**35**	Ile Europa	0				0.1		0	0						0	0	0	0	-0.2	-0.4
**36**	Ile Tromelin	0.2	1.9	1.9		0.2			-0.4						0	0	0	0	-0.2	-0.5
**37**	Mauritius	-0.2	1.2	1.4	0.1	0.6	3.4	0	-0.1	2.6	3	3.3	0	-0.1	-0.2	-0.3	-10.1	0	-0.2	-0.4
**38**	British Indian Ocean Territory	0.6	2.1	2.1		0.2			0	6	4.1	2.3	-0.2	0	0	0	-1.3	0	-0.2	-0.4
**39**	Maldives	-0.4	1.7	1.7		0.1	-0.4	-0.3	-0.3	0	2.4	4.7	-0.4	-0.3	-0.2	0	-6.3	-0.3	-0.5	-0.6

										Goal/Sub-Goal Scores										
	Country/EEZ	Index		FP		AO	NP	CS	CP		LE		TR		SP		CW		BD	
			FIS		MAR					LIV		ECO		ICO		LSP		HAB		SPP
**40**	Sri Lanka	0.1	0.1	0.5	0	0.7	-5.5	0.1	0	0	13	25.8	0.1	0	-0.1	-0.1	-8.2	0	-0.1	-0.3
**41**	Mozambique	-1.8	-0.1	-0.2	0	-0.1	-10.1	-0.3	-0.4	0	0	0	-0.1	-0.3	-0.3	-0.3	-6.7	-0.3	-0.4	-0.5
**42**	Madagascar	-1.8	0.2	0.3	0	0.1	1	0	0	-17.2	-8.7	0	0	-0.1	-0.1	-0.2	-11.3	0	-0.2	-0.3
**43**	Kenya	-1.9	0	0	0	0	-3.2	-0.1	-0.2	-0.3	-0.1	0	0	-0.3	-0.6	-0.9	-14.6	-0.2	-0.4	-0.6
**44**	Somalia	-7.2	0.2	0.2		0.2	-64.3	0.1	-0.1	-2	2.1	6.3	0	-0.1	0	0	-9.8	0	-0.1	-0.2
**45**	Eritrea	-5	-1	-1		0.1	-37.1	0	-0.1	0	0	0	-0.1	-0.1	-0.1	0	-11.6	0	-0.1	-0.3
**46**	Djibouti	-1.7	-0.2	-0.2		0			-0.2	-2	-1	0	-0.1	-0.3	-0.2	-0.1	-12.1	0	-0.2	-0.5
**47**	Yemen	0.6	-0.2	-0.2		-0.2	6.4	-0.5	-0.2	0	6.5	12.9	0	-0.3	-0.2	0	-5	-0.4	-0.5	-0.6
**48**	Oman	-1.5	-0.4	-0.4	0	-0.9	-1.7		0	0	0	0	-0.4	-0.3	-0.4	-0.3	-9	0	-0.3	-0.7
**49**	Sudan	-1.4	0.1	0.1		0.2	-14.9	0	0	3	1.5	-0.1	0	0	0	0	-1	0.1	0.1	0
**50**	Saudi Arabia	-3.4	-0.5	-0.9	-0.6	1.6	-13.9	-0.6	-0.3	0	0	0	-0.4	-0.5	-0.5	-0.4	-17.8	-0.7	-0.8	-0.8
**51**	Kuwait	-0.5	-0.1	0	0	2.3		-0.2	-0.1	0	0	0	-0.1	-0.1	0.6	1.4	-7.2	0	-0.1	-0.4
**52**	Bahrain	-0.7	-0.3	-0.3	0	0.9	0	-0.3	-0.2	11.5	9.3	7.1	-0.3	-0.3	-0.1	0	-15.5	-0.4	-0.5	-0.6
**53**	Pakistan	-0.6	-1.1	-1	0	0	0	-0.1	-0.1	-7.9	-3.7	0.6	0	-0.2	-0.2	-0.2	-0.8	-0.1	-0.3	-0.5
**54**	United Arab Emirates	-1.7	0.3	0.1	0	3.4		0	0.1	0	0	0	0	0.3	-0.9	-2.1	-2.3	0	0.1	0.3
**55**	Azores	-1.9	-0.1	-0.1		0				0.8	0.4	0	-0.1	0	-0.3	-0.6	-12.8	0	-0.2	-0.4
**56**	Cape Verde	1.4	-2.9	-2.9		0.3				0	1.6	3.3	0	0.3	0.1	0	10.7	0	0	0
**57**	Madeira	-1.9	-1.1	-1.1		0				0.5	0.2	-0.1	0	0	0	0	-12.3	0	-0.3	-0.6
**58**	Canary Islands	-1.5	-2.1	-2.1		0.2		0	0	0.6	0.1	-0.5	0.2	0	0	0	-12	0.1	-0.1	-0.4
**59**	Belgium	0.5	2.4	2.4		0.5	-1.7	0	0	25.9	12.9	0	-0.3	-0.1	0	0	-9.1	0	0	0
**60**	Gibraltar	-1.4	0	0		0				0	-0.1	-0.1	-0.1	-0.1	-0.1	0	-9.6	0	-0.1	-0.2
**61**	Tunisia	-0.7	1.3	0.4	0.3	0.4	-5.4			-0.4	-0.1	0	0	0	0	0	-0.8	0	0.1	0.1
**62**	Morocco	-0.4	-1.3	-1.3	0	0	-1.4			0	0	0	-0.1	-0.1	0	-0.1	-0.6	-0.1	-0.3	-0.5
**63**	Western Sahara	-0.5	-2.2	-2.2		1.1				-1.7	-0.5	0.6	-0.1	-0.2	-0.1	-0.1	-1.3	0	-0.3	-0.6
**64**	Mauritania	1.9	-2	-2		0.1	5.6			-1.4	-0.4	0.7	0	0.2	0.2	0	11.4	0	0	-0.1
**65**	Gambia	1	-2	-2	0	0.1		0	0	0	0	0	0	0.2	0.2	0	10.3	0.1	0	-0.1
**66**	Senegal	-0.5	-2.8	-2.8	0.1	0.2	-20	0.1	0	0	7.1	14.2	0	0.3	0.2	0	10.5	0.1	0.1	0
**67**	Libya	1.2	-1.5	-1.5	0	5.1	4.8			-0.5	3.9	8.4	-0.1	-0.6	-0.3	0	-2.1	0	-0.4	-0.8
**68**	Malta	-1.8	-0.3	-1.1	-1	0		-0.3	-0.4	-1.7	0.5	2.7	0	-0.3	-0.6	-0.9	-13.8	-0.3	-0.4	-0.4
**69**	Latvia	4.6	2.1	2.1		0.8	28.9	-0.1	0	23.8	11.9	0	0		0	0	2.6	-0.1	-0.1	-0.1
**70**	Estonia	-3.4	1.2	1.2	0	0.8	-51	0	0	1.8	11.7	21.7	0.1		0	0	3.8	0	0	0
**71**	Bulgaria	9.3	1.1	32.5	-0.3	0.2	43.6			0	-0.1	0	-0.1	-0.2	-0.1	0	-2.3	0	-0.1	-0.1
**72**	Romania	-0.1	1.1	1.1		0.2	5.5			-3.2	-1.6	0	0	-0.2	-0.1	0	-5.6	0	-0.1	-0.2
**73**	Russia	-1.2	0	0	0.5	0.4	0.6	0	-0.9	10.9	5.4	0	-0.2	-0.2	-0.6	-0.9	-16.4	0	0.4	0.7
**74**	Georgia	1.6	1.5	1.5		0.5	-0.9			-0.1	17.7	35.5	0.1	0.1	0	0	-6.4	0	0.2	0.2
**75**	Ukraine	-4.9	1.1	2.6	0	0.2	-43.9	-0.3	-0.4	0	0	0	0	-0.3	-0.3	-0.4	-5.9	-0.2	-0.2	-0.2
**76**	Turkey	-3.3	-0.9	-1.4	0	0.3	-1.6			0	0	0	-0.2	-0.3	-0.3	-0.2	-23.2	0.1	-0.1	-0.3
**77**	Syria	-1.4	-1.4	-1.4		0	-3.6			0	0	0	-0.6	-0.5	-0.2	0	-5.3	-0.8	-0.8	-0.8
**78**	Lebanon	3.8	-1.1	-1.1		0.2				-1.5	-0.5	0.5	0	-0.1	0	0	-5.2	-0.1	-0.2	-0.3
**79**	Israel	-0.6	0.2	-1.9	0	1	-9.2	0	0	0	0	0	0.1	0.1	0	0	3.9	0	0.1	0.3
**80**	Greece	-1.9	-1.3	-0.7	0.2	-0.8	-8.9			-2.5	-3.9	-5.3	0	-0.2	-0.2	-0.2	-0.8	0	-0.2	-0.3

Goal/Sub-Goal Scores

	Country/EEZ	Index		FP		AO	NP	CS	CP		LE		TR		SP		CW		BD	
			FIS		MAR					LIV		ECO		ICO		LSP		HAB		SPP
**81**	Cyprus	0.9	0	16.8	1.1	-0.4	0			-4.6	-2.2	0.2	-0.3	-0.1	-0.4	-0.7	-5.9	0	-0.1	-0.2
**82**	Albania	-3.1	-1.2	-1.7	0	-0.1	-11.8			0	0	0	0	-0.1	-0.5	-0.7	-10.7	0	-0.1	-0.2
**84**	Algeria	-0.1	-0.1	0	0	0.2				0	0.2	0.4	-0.1	-0.1	-0.1	0	-0.9	0	-0.1	-0.2
**85**	Ascension	0.6	-0.1	-0.1		0.4				-3.7	0.6	4.9	0.1		0	0	3.7	0	-0.2	-0.4
**86**	Saint Helena	-0.9	3.3	3.3		0.4				-23.1	-13.1	-3.2	0.1	0	0	0	3.6	0	-0.2	-0.5
**88**	Tristan da Cunha	0.5	-1.2	-1.2		0.4				-3.7	0.6	4.9	0		0	0	3.7	0	0	-0.1
**89**	South Georgia and the South Sandwich Islands	11.8	-1.8	-1.8										0.6	47.6	94.7	0	2	1.5	1
**90**	Prince Edward Islands	0	0	0											0	0	0	0	0	0.1
**91**	Crozet Islands	-0.3	-1.2	-1.2										-0.1	0	0	0		-0.1	-0.1
**92**	Amsterdam Island and Saint Paul Island	-0.3	-1.1	-1.1										-0.1	-0.1	0	0	0	-0.1	-0.2
**93**	Kerguelen Islands	-1.6	-6.4	-6.4										-0.1	0	0	0	0	-0.1	-0.2
**94**	Heard and McDonald Islands	0.4	1.2	1.2											0	0	0	0	0	0.1
**95**	Falkland Islands	0	-4.1	-4.1	0	0.5				-1	-0.7	-0.5	0.1	0	-0.1	0	3.7	0	0	0
**96**	Sierra Leone	0.9	-2.3	-2.3		-0.8		-0.6	-0.1	-0.3	-0.3	-0.4	0	-0.6	3.8	8.1	9.1	-0.2	-0.6	-1
**97**	Liberia	0.7	-3	-3		0		0	0	0	0	0	0	0.1	0.1	0	9.6	-0.1	-0.1	-0.2
**98**	Togo	0.8	-1	-1		0.1		-0.1	-0.2	0	0.4	0.7	0	0.1	0.1	0	8.6	0	0	-0.2
**99**	Benin	0.5	-3	-3		0		0	0	1.3	0.6	0	0.1	0.1	0	0	7.6	0	-0.1	-0.2
**100**	Republique du Congo	1.2	-3	-3		0.1		0		0.6	12.7	24.7	0	-0.1	0	0	0.3	0	-0.4	-0.6
**101**	Namibia	1.4	0	0.1	-2.3	0.3	-6.3			0	11.1	22.2	0.1	0.1	0	0	6.1	0	-0.1	-0.2
**102**	South Africa	2.5	0	0.1	0	0.3	7.2	0	0	0	12.8	25.6	0	0.1	0.2	0.3	5.1	0	-0.1	0
**103**	Sao Tome and Principe	-0.3	-3	-3		0.1				0	0	0	0.1	0	0	0	0.6	0	-0.1	-0.2
**104**	Equatorial Guinea	-0.3	-4	-4		0.7		0	0	0.1	0	-0.1	0	0	0	0	0.4	0	-0.1	-0.4
**105**	Bouvet Island	-0.8	-3.4	-3.4											0	0	0		0.1	0.1
**106**	Ghana	-3.2	-1	-1		0.2	-45.8	-0.2	0	0	3.9	7.8	0	0.2	0.1	0.1	11.1	0	-0.1	-0.1
**107**	Clipperton Island	0.2	1	1		0.1									0	0	0	0	-0.2	-0.5
**108**	Bermuda	-1.7	0	0		-0.3			0	0	-7.9	-15.9	0	0	-0.1	0	-5.2	-0.1	-0.2	-0.5
**110**	Bahamas	-1.5	2.1	2.1	0	0.4	-25.7	0	0	13.2	7.8	2.4	0	-0.1	0	0	1.1	0	-0.1	-0.3
**111**	Turks and Caicos Islands	1	-2.2	-2.2	0	-0.2	9.7	-0.2	-0.1	1.3	1.9	2.3	0	0	0	0	2.1	-0.2	-0.2	-0.5
**112**	Cuba	0.7	0	0	0	0	1.1	0	0	-0.1	4.4	8.9	0	0	0	0.1	2.1	-0.1	0	0
**113**	Cayman Islands	0.8	0	0		-0.1		0.2	0	0	0	0	0.3	0.1	0.3	0.4	6.4	0.1	0	0
**114**	Haiti	0.2	0	0		0	-3.3	-0.3	-0.2	0	4.2	8.5	0	-0.1	0	0	1.4	-0.1	-0.2	-0.2
**115**	Dominican Republic	-0.3	-1.1	-0.7	0	0.4	-11.7	-0.1	-0.2	0.3	4.2	8.1	0	0	0.1	0	5.9	-0.1	-0.1	-0.1
**116**	Puerto Rico and Virgin Islands of the United States	2	-1	-1	0	0.8		0	0	1.2	3	4.8	0.1	0.2	0.4	0.6	14.7	0	0	0.1
**117**	British Virgin Islands	-0.3	0	0		-0.2		-0.2	-0.2	-0.4	4	8.3	0	-0.1	-0.2	0	-5.7	-0.2	-0.4	-0.7
**118**	Anguilla	-0.2	-1.1	-1.1		-0.1		-0.1	-0.2	0	-2.5	-5.1	0	0	0	0	2.8	-0.1	-0.2	-0.4
**119**	Saint Kitts and Nevis	0.1	0	0	0	0.1		0	-0.3	0	5.3	10.8	0	-0.1	0	0	-3.7	0	-0.1	-0.1

Goal/Sub-Goal Scores

	Country/EEZ	Index		FP		AO	NP	CS	CP		LE		TR		SP		CW		BD	
			FIS		MAR					LIV		ECO		ICO		LSP		HAB		SPP
**120**	Antigua and Barbuda	-0.3	-1.1	-1.1		0.7		0	-0.1	0	-4.4	-8.8	0	0	0	0.1	1.9	-0.2	-0.3	-0.4
**121**	Montserrat	0.3	0	0		-0.4		-0.4	-0.5	-0.4	2	4.3	0	-0.1	-0.1	0	1.9	-0.3	-0.3	-0.4
**122**	Saint Lucia	0.6	0.1	0.1		0.1	0	0	-0.2	-0.7	4.4	9.4	0	0	0	0	1.8	0	0	0
**123**	Dominica	0.3	0	0		0.1		-0.1	-0.1	-3.5	6	15.5	0	-0.1	-0.1	0	-3.2	-0.1	-0.2	-0.3
**124**	Barbados	-1.1	-2	-2		0.5	0	0	0	0.1	-1.6	-3.5	0	0.1	0	0	-7.1	0.1	0	-0.2
**125**	Grenada	-1.5	-1.1	-1.1		0.1		-0.1	-0.3	0	-0.2	-0.5	-0.3	-0.2	0	0	-11.3	-0.1	-0.2	-0.3
**126**	Trinidad and Tobago	1.2	0.1	0.1		0.7	6.6	0	-0.2	0	7	14.1	0.1	0	0	-0.1	-3.1	-0.1	0	-0.1
**127**	Saint Vincent and the Grenadines	-0.7	-1	-1		0.3	0		-0.3	-0.3	3	6.4	0	-0.1	-0.1	0	-7.7	0	-0.1	-0.2
**129**	Panama	-1.6	1	1.4	0	1	-0.7	0	0	0	0.2	0.4	0	0	-0.2	-0.2	-17.5	0.1	-0.1	-0.2
**130**	Costa Rica	-4.2	2	2.7	-0.3	0.4	-48.3	-0.1	-0.2	6.6	5.5	4.5	-0.1	-0.2	-0.2	-0.2	-0.9	-0.2	-0.4	-0.6
**131**	Nicaragua	1.2	0.1	-2.1	-0.8	0.2	7	0	0	-5.9	-2.9	0	0.1	0.2	0.5	0.8	8.7	0	0.1	0.1
**132**	Colombia	1.1	1.1	3.2	-1	0.6	-0.3	0.2	0.1	0.4	1.8	3.4	0.1	0.2	0.5	0.8	4.5	0.2	0.2	0.2
**133**	Honduras	-1	0.1	1.2	-0.3	0.3	6.2	0.1	0	2.9	1.5	0	0	-0.2	-0.5	-0.9	-18.3	0.1	-0.1	-0.3
**134**	El Salvador	-0.4	1.1	1.4	0	0.2	-5.6	0	0	6.7	3.4	0	0	-0.1	0	0	-3.2	0	-0.2	-0.3
**135**	Mexico	0.7	0	0	-1.5	0.4	-4.8	0	0	4.6	2.3	0	0	0.1	0.1	0	8.7	0	0	0
**136**	Guatemala	0.4	8.9	12.2	1.9	0.1	-3.7	0	0	0	0	0	0	-0.1	0	0	-3.9	-0.1	-0.1	-0.4
**137**	Ecuador	2.4	2.1	4.2	1.4	0.3	-1.5	0.1	0.1	0.6	15.6	30.5	0.1	0.1	0.1	0	5.5	0	0	-0.1
**138**	Peru	1.6	3.3	3.7	5.7	0.5	-2.7	0.1	0.2	4.5	9	13.5	0.1	0.1	0.2	0.2	5.3	0.1	0.1	0
**139**	Venezuela	1.7	1.1	-1.4	0.2	0.6	0	0	-0.1	30	18.9	7.7	0	0	0.1	0.1	-1.1	-0.1	-0.1	-0.1
**140**	Guadeloupe and Martinique	0.3	0.1	0	0	-0.1	0	0	-0.1	0	1	2	0	0	0.1	0.2	2	0	-0.1	-0.1
**141**	Faeroe Islands	1.5	0.7	2.7	0	-0.3	5.2			-3.7	0.9	5.6	0.2	-0.6	-0.3	0	3.9	0	-0.4	-0.8
**143**	Iceland	1.3	2.5	2.5	2.5	0.6	-0.2		0.7	0	1.9	3.8	0.3	0.2	0.2	0.2	3.8	3.7	2	0.3
**144**	Jan Mayen	1.4	2.4	2.4		0.8				-3.7	1.4	6.6	0.1	0	0	0	4.8	0.2	0.1	0.1
**145**	Greenland	0.9	1.2	1.2		0.7			0.4	0	0	0	0.3	0.1	0	0	4	1.1	0.7	0.2
**146**	Pitcairn	0	1.1	1.1		0			0	0.3	10.3	20.3	-0.4	-0.3	-0.1	0	-10.4	0	-0.4	-0.7
**147**	French Polynesia	0.9	1	3	4.2	0	-0.6	-0.2	-0.3	0	17.3	34.4	-0.5	-0.3	-0.2	0	-9.1	0	-0.4	-0.7
**148**	Line Group	0.6				0.3			0	0.5	4.3	8.1	0.1		0	0	-0.5	0.1	0	-0.2
**149**	Jarvis Island	0.2	1.1	1.1		0									0	0	0	0	-0.2	-0.2
**150**	Palmyra Atoll	0	0	0		0			0					0	0	0	0	0	-0.1	-0.3
**151**	American Samoa	0.3	1.1	1.1		0		0	0	0	9.7	19.4	0	-0.2	-0.3	-0.4	-7.2	0	-0.3	-0.6
**152**	Samoa	-0.6	1.2	1.2	0	0.1		0.1	0.1	0.3	6	11.7	-0.4	-0.1	0	0	-12.1	0	-0.2	-0.4
**153**	Cook Islands	-7.7	-0.6	-0.6	0	-0.7	-63.7	-0.6	-1.2	0.4	6	11.7	-0.8	-0.9	-0.5	0	-13.2	-1	-1.2	-1.5
**154**	Niue	-2.1	0.4	0.4		-0.8		-0.6	-1.1	-0.9	-0.8	-0.8	-0.8	-0.9	-0.5	0	-13.2	-1	-1.3	-1.6
**155**	Tonga	0.2	1.1	1.1	0	0.1	-1	0	0	0	12.7	25.5	-0.4	-0.1	0	-0.1	-10.4	0	-0.2	-0.3
**156**	Tokelau	-0.1	1.2	1.2		0.3		0.1	0.3	0.3	9.5	18.7	-0.3	0	0	0	-11.3	0.2	-0.1	-0.3
**157**	Phoenix Group	0.6				0.4			0	0.5	4.3	8.2	0		0	0	-0.5	0	-0.1	-0.2
**158**	Howland Island and Baker Island	0.1	0.5	0.5		0.2			0						0	0	0	0	-0.1	-0.2
**159**	Johnston Atoll	0.2	1.1	1.1		0			-0.1					0	0	0	0	0	-0.2	-0.4

Goal/Sub-Goal Scores

	Country/EEZ	Index		FP		AO	NP	CS	CP		LE		TR		SP		CW		BD	
			FIS		MAR					LIV		ECO		ICO		LSP		HAB		SPP
**161**	Wallis and Futuna	-0.3	0	0		0			0	0.3	9.7	19.1	-0.4	-0.3	-0.1	0	-11.6	0	-0.3	-0.6
**162**	New Zealand	0.9	-1	-1	0	0.6	13.1	0.2	0.1	0	1.4	2.8	-0.6	0	-0.4	-0.8	-4.7	0.2	0.1	0
**163**	United States	8.4	1.2	1.3	-0.3	1.3	1.3	0.1	1.8	-2.6	-1.3	0.1	0.6	0.4	0.2	0	78.6	0	0.5	0.9
**164**	Belize	-2.5	-0.1	5	6.2	0.1	-28.2	-0.1	-0.1	0	0	0	-0.2	-0.1	0	0	-0.6	-0.1	-0.1	-0.1
**166**	Jamaica	0.6	0	0.1	0	0.4	6.9	0.2	0	-0.7	2.3	5.4	0.3	0.1	0.1	0	-4.1	0.1	0.1	-0.1
**167**	Guyana	1.4	0	0	-0.2	0.2		-0.1	-0.1	-8.6	9.6	28	0	0	-0.1	0	2.6	-0.1	-0.1	-0.3
**168**	Suriname	1.1	0	0.1	0.1	0.5		0	0	-3	7	17.1	0	0	0	0	2	0	0	-0.1
**169**	French Guiana	0.4	-1.1	-1.1		0.4		0	0	4.4	2.4	0.3	0.1	-0.1	0	0	2.4	0	-0.1	-0.4
**171**	Brazil	2.1	0.9	0.9	0.1	0.2	-2.2	-0.1	-0.2	19.1	10.4	1.8	0	0	0	0	11.4	-0.2	-0.2	-0.4
**172**	Argentina	-1.1	-1.6	-1.6	0	0.7	1.7			-0.2	-0.1	0	-0.1	0.2	0	-0.2	-8.9	0	0.2	0.4
**173**	Uruguay	-0.8	-1.2	-1.2	0	0.4	0.5			0	0	0	0	-0.2	-0.1	-0.1	-6.3	0	-0.1	-0.2
**174**	Finland	0.2	1.2	-0.1	-0.7	0.5	-6.8		0	14.4	7.2	0	0.1		0	0	1.7	0	0	0
**175**	Denmark	1.1	0.5	0.7	-0.7	0.7	0	0	0	4.9	5.4	5.7	0.1	0.2	0.1	0	3.5	1.2	0.9	0.5
**176**	Germany	-0.4	1.4	1.8	3.3	1.2	0.5	0	0.1	6	3	0	-0.2	-0.1	0	0	-9.9	0	0	0.2
**177**	Netherlands	-0.6	1.6	1.4	0.6	0.5	-4.1	0	0	0.1	1.7	3.2	0	0	0	0	-8	4.4	2.4	0.4
**178**	Poland	-4.6	2.5	2.5		0.7		0	0.1	2.4	1.2	0	0		0	0	-13.6	0.1	0	0
**179**	France	-0.9	1.1	-0.5	-1.6	0.1	-9.6	-0.2	4	-0.2	-0.1	0	-0.5	-0.2	-0.1	0	-1.7	0.4	0.1	-0.2
**180**	United Kingdom	1.3	1.1	-0.2	-0.1	0.4	7.5	-0.1	8.9	-7.5	-1.2	5.1	-0.5	-0.1	-0.1	0	-5	5.9	3	-0.1
**181**	Ireland	0.1	2.3	1.8	-1.7	0.9	-4	-0.1	-0.1	0.1	2.2	4.1	-0.1	0	0	0	-0.2	-0.1	0.1	0.1
**182**	Spain	-0.7	1.2	-0.2	0.9	0.3	-3.3	0	0	-0.9	-0.5	0	0.1	0.1	0	0	-3	0.1	0.1	0.1
**183**	Portugal	-2	1	2.3	0.2	-0.1	-6.4			0.9	0.4	0	-0.1	-0.2	-0.1	0	-11.9	0	-0.2	-0.4
**184**	Italy	-0.2	0	4.3	-0.5	0.1	4.1	0	-0.1	4.1	2	0	-0.1	-0.1	-0.1	0	-12.9	0	-0.1	-0.2
**185**	Monaco	-1	0.8	0.8		0.1				4.1	2.1	0.1	-0.4	-0.1	-0.4	-0.7	-9		-0.4	-0.4
**186**	Montenegro	-1.7	1	1.2	-0.1	-0.2	0			-0.1	-1.3	-2.4	0	0	0	0	-12.9	0	0	-0.1
**187**	Croatia	-3	1.2	1.7	-1.6	0.4	2.6			-0.7	-0.4	0	0	-0.2	-0.5	-0.7	-27.2	0	0	-0.1
**188**	Slovenia	-3.5	0.6	3.5	-0.4	0.5	-9.6			0	0	0	-0.4	-0.2	-0.1	0	-21.9	0	0.1	0.1
**189**	Lithuania	0.7	2.1	2.1		0.7	12		-15.2	12.5	6.2	0	0		0	0	2.9	-3.2	-1.7	-0.1
**190**	Qatar	1.1	-1.3	-1.3	0	0		-0.5	-0.3	0.2	21.2	42.2	-0.2	-0.4	-0.2	0	-8.7	0	-0.2	-0.5
**191**	Iran	1.4	0.2	0	0.5	0.6	0	0.2	0	0	13.2	26.5	0.1	0.2	0.2	0.2	-0.5	0.2	0.3	0.3
**192**	Iraq	1.9	0.4	0.4		0.9		0.2	0.2	0	18.1	36	0	0.2	0.1	0.1	-2.9	0.3	0.4	0.4
**193**	Guinea Bissau	1.6	-3.1	-3.1		0		0	-0.1	-1.4	8	17.5	0	0.1	0	0	9.9	-0.1	-0.1	-0.1
**194**	Guinea	1	-2.9	-2.9		0.1		0	0	-0.1	0.2	0.4	0	0.2	0.6	0.9	11	0.2	0	0
**195**	Ivory Coast	1.4	-1.7	-1.7		0.2	-3	0.1	0	-1.5	7.7	16.9	0.1	0.2	0.4	0.5	9.7	0.2	0.1	0
**196**	Nigeria	5	-2.8	-2.8	0	0.2		0	0	0	6	11.9	0	0.1	0.1	0.1	2.6	0.1	-0.1	-0.2
**197**	Cameroon	-0.1	-2	-2		0.1		0	0	1.9	0.9	0	0	0	0	0	0.3	-0.1	-0.1	-0.2
**198**	Gabon	1.3	-1.9	-1.9		0.7		0.1	0.1	1.2	12.8	24.5	0	0	0.1	0	0.4	0.1	-0.2	-0.3
**199**	Democratic Republic of the Congo	0	-2	-2		-0.1		0	0	0	3	6	0	-0.1	0	0.1	-0.1	-0.1	-0.3	-0.6
**200**	Angola	0.8	0	0		0.1	-1.6	-0.2	-0.1	1.5	10.1	18.6	0	-0.1	-0.1	-0.1	0.4	-0.1	-0.3	-0.4
**202**	Tanzania	-1	0	-0.2	0	0	0.6	-0.2	-0.2	0	0	0	0	-0.2	-0.1	0	-9.4	-0.2	-0.3	-0.5
**203**	India	0.4	0	-2.3	-0.8	0.1	-2.3	-0.1	-0.2	8.3	4.1	0	0	-0.1	0	0	5.6	-0.2	-0.3	-0.3

	Country/EEZ	Index	Goal/Sub-Goal Scores																	
			FP			AO	NP	CS	CP	LE			TR	SP			CW	BD		
			FIS	FP	MAR					LIV	LE	ECO		ICO	SP	LSP		HAB	BD	SPP
**204**	Bangladesh	0.7	0	0.2	0.4	0.4	2.9	0	0	0	0.4	0.6	0	0.1	0.2	0.2	3.3	0	0.2	0.4
**205**	Myanmar	7.1	0	-0.1	-1.3	0.9		0.3	0.1	0	8.9	17.7	0	0.2	0.2	0.1	8.5	0.2	0.2	0.1
**206**	Malaysia	0.5	-3.2	-2.7	-0.2	0.8	22.6	-0.2	-0.2	0	-5.7	-11.5	-0.1	-0.2	-0.2	-0.2	-9	-0.2	-0.1	-0.1
**207**	Vietnam	1.1	0	4.9	1.9	0.2	-4.2	0	0	0	6.8	13.5	0	0.1	0.1	0	2.8	0.1	0.1	0.1
**208**	Singapore	1.9	-2.2	3.4	-0.2	2.2	0.5	0	0	2.1	1.1	0	0.1	0.2	0.1	0	11.8	0.1	0.2	0.3
**209**	China	0	0	0.5	0	0.3	1.4	-0.1	0.2	1.6	0.6	-0.4	0	-0.1	0	0	-2.4	0	-0.1	0
**210**	Japan	-0.2	1.1	0.5	-2.7	1.2	-3.5	0	1.2	10.8	5.3	0	0	0.3	0.2	0	-6.2	0	0	-0.2
**212**	Kiribati	0.6	0.1	0.1	-0.1	0.1	0.3	0.1	0.1	11.2	5.6	0	0.1	0.1	0	0	-0.3	0.1	0	0
**213**	Antarctica																			
**214**	Egypt	2.5	-1.2	-2.8	-2.8	-0.1	30.9	-0.7	-0.5	0	0	0	-0.5	-0.3	-0.5	-0.6	-1.3	0	-0.2	-0.4
**215**	Jordan	-0.9	0	0		0		-0.1	-0.2	-0.7	-0.4	0	0	-0.2	-0.1	0	-7	-0.2	-0.4	-0.5
**216**	Indonesia	1.1	-1.1	3.2	1.5	0.4	-2.3	0.1	0	0	15.8	31.6	0	0	-1.1	-2	-5.5	0.1	-0.1	-0.1
**218**	Canada	3.1	1.1	1.3	0.8	0.6	-7.9	0	-0.8	-4.2	-2.1	0	0.5	0.3	0.3	0.3	38.6	0	0.2	0.4
**219**	Saint Pierre and Miquelon	7.4	2.3	2.3		1				0	0.1	0.1	0.6	0.1	0.1	0.2	47.8	0	0.2	0.2
**220**	Sint Maarten	-3.1	0	0		0	-26.5	0	-0.1	-0.4	1.8	4.2	0	-0.1	-0.1	-0.1	-6.4	0	0	0
**221**	Northern Saint-Martin	0.4				-0.2		0	-0.2	-0.4	1.8	4.1	0		-0.1	-0.1	1.8	0	0	-0.1
**222**	Sweden	2.4	1.5	1.2	-0.2	1	0.9	0.1	0	0	0	0	1.1	0.4	0.2	0	19.6	0	0	0
**223**	Norway	0.2	1.2	-0.6	0	1.7	-0.5		-12.5	20	12.8	5.7	0.3	0	0	0	1.4	-1.4	-0.7	0.1
**224**	Chile	-1	2.3	0.6	0	0.6	-5.7			0	1.1	2.3	-0.1	0	0	0	-4.4	-0.1	-0.1	-0.3
**227**	Jersey	0.6				0.6		0	0	-3.7	1.2	6.2	-0.1		0	0	2.8	0	0	0
**228**	Guernsey	0.6				0.6		0	-0.1	-3.7	1.2	6.2	0		0	0	2.8	0	0	0.1
**231**	East Timor	0.8	0	0.1	0	0.2		0	0	0.1	0.1	0	0	0.1	-1.7	-3.4	8.7	0	0.1	0.2
**232**	Bosnia and Herzegovina	-0.5	1	5.9	0.1	0				0	0	0	0	-0.1	-0.3	-0.4	-9.2	0	-0.1	-0.1
**237**	Oecussi Ambeno	2				0.8		0	-0.2	-1.5	7.1	15.8	0		0.2	0.2	8	0	0.1	0.2
**244**	Curacao	0.6				-0.1		0.1	0	0	1.4	2.8	0	0.1	0.1	0.1	3.4	-0.1	0	0
**245**	Bonaire	-2.6			0.1	-0.1	-26.6	0.1	-0.3	0	1.3	2.7	0	0.1	0.1	0.1	2.1	-0.1	0	0
**247**	Brunei	0.2	-2.3	-7.4	0.1	1.6		0	0	0	-7.6	-15.4	0	0.2	0.1	0.1	14.8	0	0.1	0.1
**248**	Saba	-2.6				-0.1	-26.7	0	0	0	1.4	2.8	0	0.1	0.1	0.2	1.9	0	0	0
**249**	Sint Eustatius	-2.6				-0.1	-26.6	0	-0.4	0.1	1.4	2.7	0	0.1	0.1	0	1.8	0	0	0.1
**250**	Aruba	1.5	0	0		-7.6		-0.2	-0.3	0	0	0	0	0.3	0.1	0	21.1	-0.2	0.1	0.3

Table N. BIC model results for OHI scores as a function of HDI, human population, and GDP. The best model fit is indicated in bold, with regression statistics for this model provided below. OHI scores tended to be higher for countries with higher Human Development Index scores according to a linear regression model (R^2 = 0.30, N=212, $F_{1,210}$=88.42; see also Fig. 4 in the main text).

**Model**	**df**	**BIC**	**AIC**	**R^2**
**HDI**	**3**	**1213**	**1203**	**0.34**
ln(GDP)	3	1272	1262	0.09
ln(population)	3	1281	1272	0.04
HDI + ln(population)	4	1218	1205	0.34
HDI + ln(GDP)	4	1215	1202	0.36
ln(population) + ln(GDP)	4	1245	1232	0.24
HDI + ln(population) + ln(GDP)	5	1218	1202	0.36

**Coefficients**	**Estimate (± 95 CI)**	**St. Error**	**t-value**	**P-value**
Intercept	39.96	2.67	14.95	--
HDI	32.59	3.47	9.40	<0.001

8. Supplemental Figures

Figure A. Maps of annual difference in scores (2013 minus 2012) for each of the 10 goals.

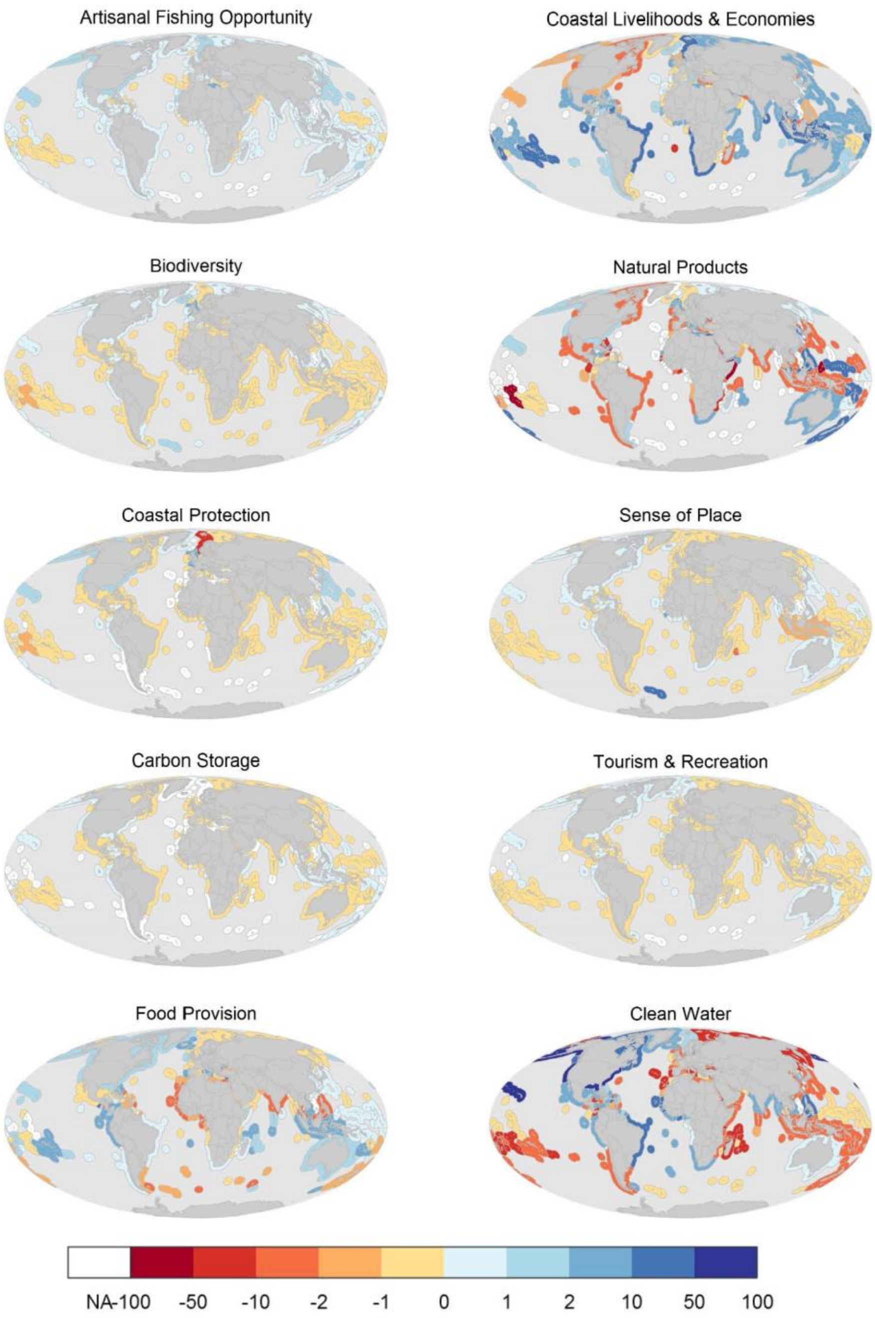

Figure B. Maps of annual difference in scores (2013 minus 2012) for each of the 8 sub-goals.

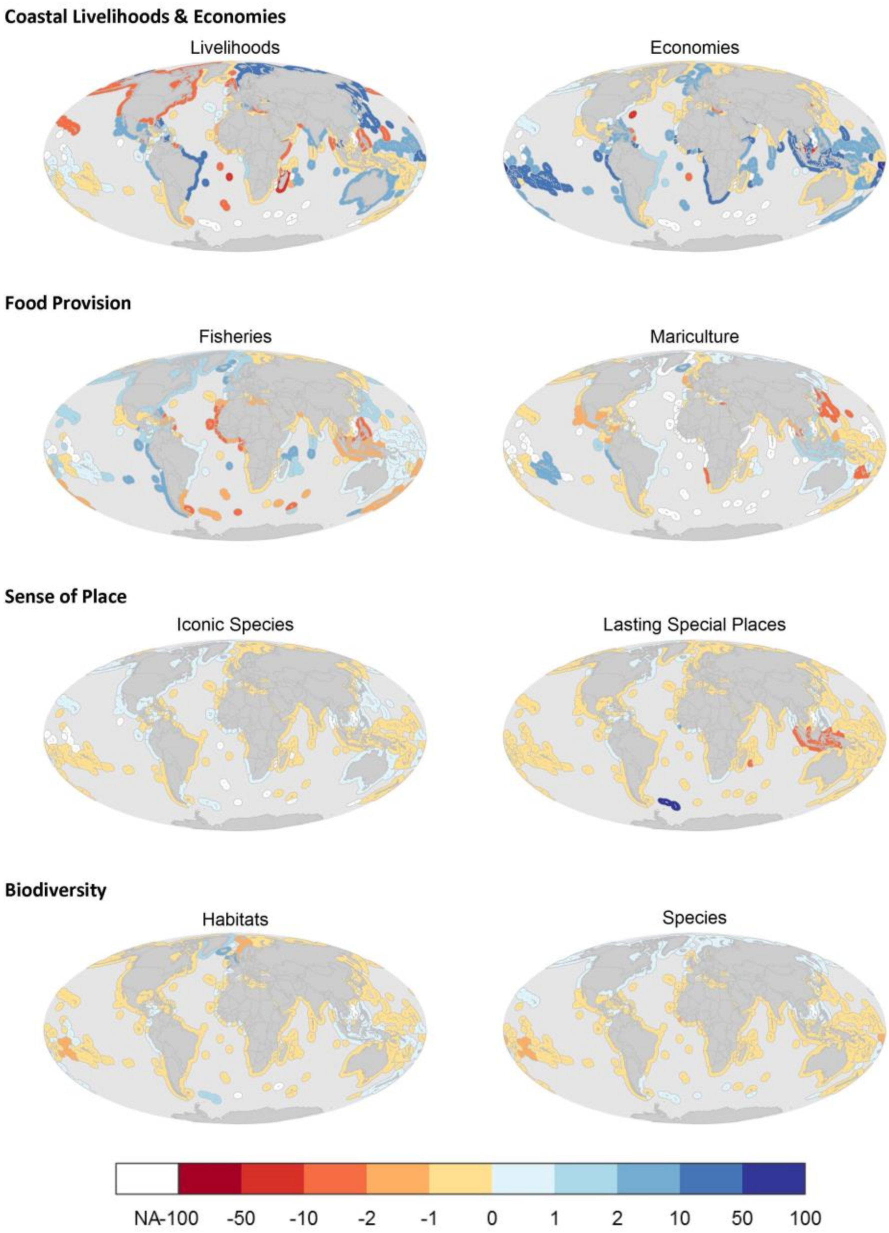

Figure C. Summary of spatial gap-filling for data within each goal for the status, pressures, and resilience measures for each reporting region. Regions (rows) are clustered by continent and larger geographies. Colors indicate no gap-filling (light blue), partial gap-filling (medium blue), and fully gap-filled (dark blue).

(see following pages)

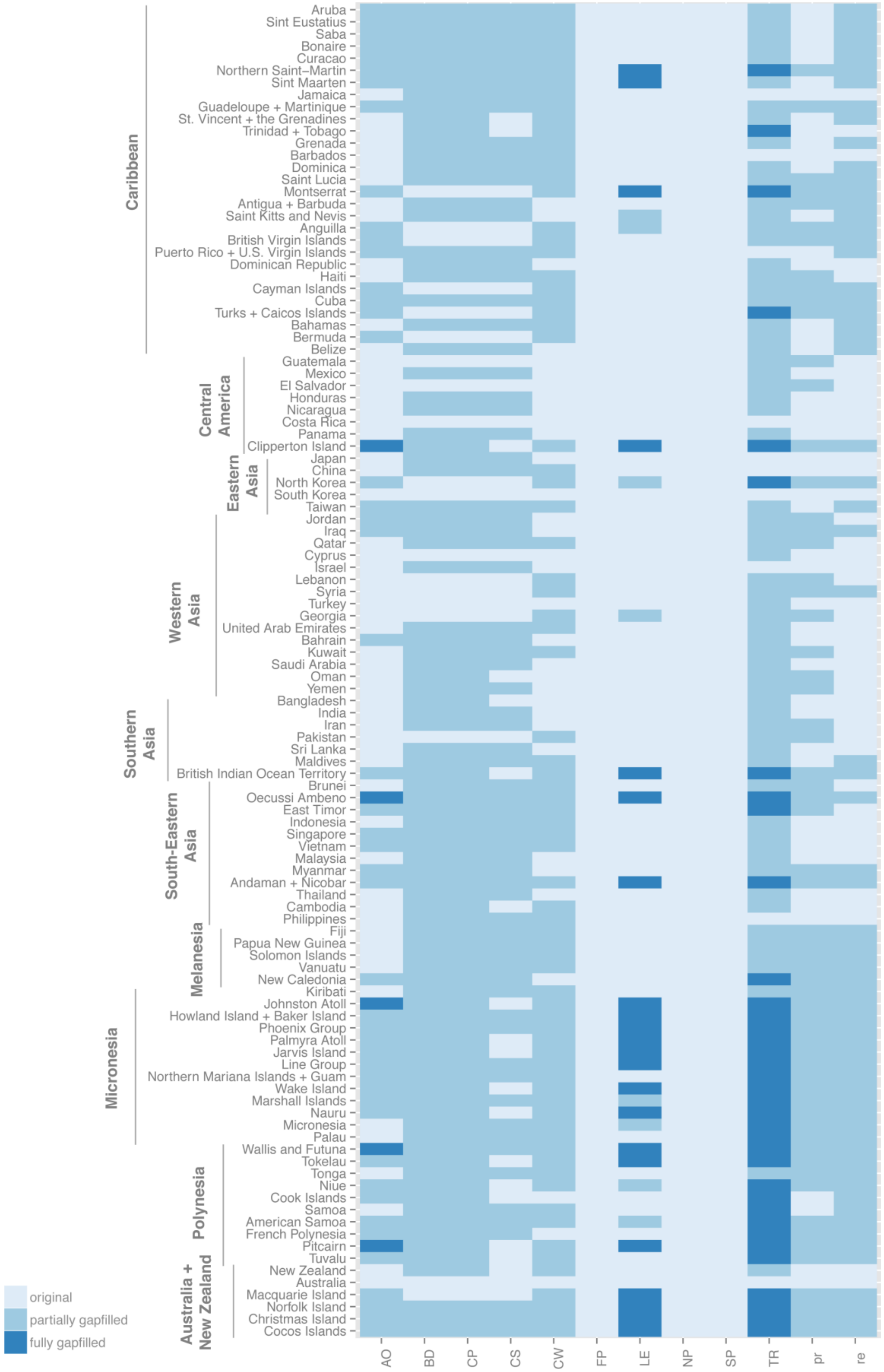
Caribbean
Central America
Eastern Asia
Western Asia
Southern Asia
South-Eastern Asia
Melanesia
Micronesia
Polynesia
Australia + New Zealand
Aruba
Sint Eustatius
Saba
Bonaire
Curacao
Northern Saint−Martin
Sint Maarten
Jamaica
Guadeloupe + Martinique
St. Vincent + the Grenadines
Trinidad + Tobago
Grenada
Barbados
Dominica
Saint Lucia
Montserrat
Antigua + Barbuda
Saint Kitts and Nevis
Anguilla
British Virgin Islands
Puerto Rico + U.S. Virgin Islands
Dominican Republic
Haiti
Cayman Islands
Cuba
Turks + Caicos Islands
Bahamas
Bermuda
Belize
Guatemala
Mexico
El Salvador
Honduras
Nicaragua
Costa Rica
Panama
Clipperton Island
Japan
China
North Korea
South Korea
Taiwan
Jordan
Iraq
Qatar
Cyprus
Israel
Lebanon
Syria
Turkey
Georgia
United Arab Emirates
Bahrain
Kuwait
Saudi Arabia
Oman
Yemen
Bangladesh
India
Iran
Pakistan
Sri Lanka
Maldives
British Indian Ocean Territory
Brunei
Oecussi Ambeno
East Timor
Indonesia
Singapore
Vietnam
Malaysia
Myanmar
Andaman + Nicobar
Thailand
Cambodia
Philippines
Fiji
Papua New Guinea
Solomon Islands
Vanuatu
New Caledonia
Kiribati
Johnston Atoll
Howland Island + Baker Island
Phoenix Group
Palmyra Atoll
Jarvis Island
Line Group
Northern Mariana Islands + Guam
Wake Island
Marshall Islands
Nauru
Micronesia
Palau
Wallis and Futuna
Tokelau
Tonga
Niue
Cook Islands
Samoa
American Samoa
French Polynesia
Pitcairn
Tuvalu
New Zealand
Australia
Macquarie Island
Norfolk Island
Christmas Island
Cocos Islands
AO
BD
CP
CS
CW
FP
LE
NP
SP
TR
pr
re
original
partially gapfilled
fully gapfilled

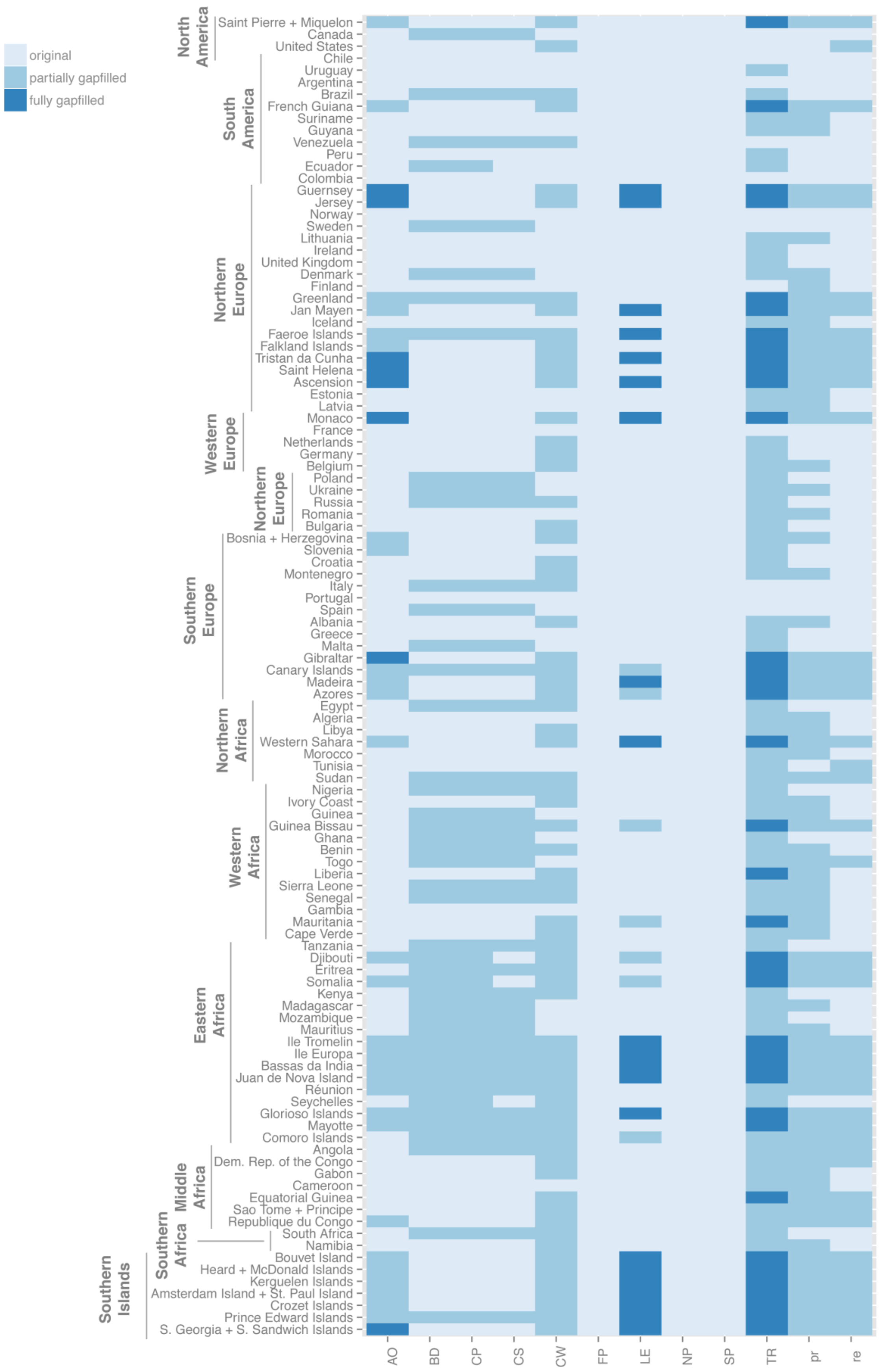

original
partially gapfilled
fully gapfilled
North America
Saint Pierre + Miquelon
Canada
United States
South America
Chile
Uruguay
Argentina
Brazil
French Guiana
Suriname
Guyana
Venezuela
Peru
Ecuador
Colombia
Northern Europe
Guernsey
Jersey
Norway
Sweden
Lithuania
Ireland
United Kingdom
Denmark
Finland
Greenland
Jan Mayen
Iceland
Faeroe Islands
Falkland Islands
Tristan da Cunha
Saint Helena
Ascension
Estonia
Latvia
Western Europe
Monaco
France
Netherlands
Germany
Belgium
Northern Europe
Poland
Ukraine
Russia
Romania
Bulgaria
Southern Europe
Bosnia + Herzegovina
Slovenia
Croatia
Montenegro
Italy
Portugal
Spain
Albania
Greece
Malta
Gibraltar
Canary Islands
Madeira
Azores
Northern Africa
Egypt
Algeria
Libya
Western Sahara
Morocco
Tunisia
Sudan
Western Africa
Nigeria
Ivory Coast
Guinea
Guinea Bissau
Ghana
Benin
Togo
Liberia
Sierra Leone
Senegal
Gambia
Mauritania
Cape Verde
Eastern Africa
Tanzania
Djibouti
Eritrea
Somalia
Kenya
Madagascar
Mozambique
Mauritius
Ile Tromelin
Ile Europa
Bassas da India
Juan de Nova Island
Réunion
Seychelles
Glorioso Islands
Mayotte
Comoro Islands
Middle Africa
Angola
Dem. Rep. of the Congo
Gabon
Cameroon
Equatorial Guinea
Sao Tome + Principe
Republique du Congo
Southern Africa
South Africa
Namibia
Southern Islands
Bouvet Island
Heard + McDonald Islands
Kerguelen Islands
Amsterdam Island + St. Paul Island
Crozet Islands
Prince Edward Islands
S. Georgia + S. Sandwich Islands
AO
BD
CP
CS
CW
FP
LE
NP
SP
TR
pr
re

9. References

AVISO (2013). www.aviso.oceanobs.com/en/news/ocean-indicators/mean-sea-level.html.

Blanke J. & Chiesa, T. (2013). The Travel and Tourism Competitiveness Report 2013. http://reports.weforum.org/travel-and-tourism-competitiveness-report-2013/

Bridgham S.D., Megonigal J.P., Keller J.K., Bliss N.B. & Trettin. (2006). The carbon balance of North American wetlands. *Wetlands* 26(4): 889-916.

Bruno J.F. & Selig E.R. (2007). Regional decline of coral cover in the Indo-Pacific: timing, extent, and subregional comparisons. *PLoS ONE* 2(8): e711.

Burke L., Reytar K., Spalding M., & Perry A. (2011). Reefs at Risk Revisited. World Resources Institute, Washington, DC

Casey K.S., Evans R.H., Baringer W., Kilpatrick K.A., Podesta G.P., Walsh S., Williams E., Brandon T.B., Byrne D.A., Foti G., Li Y., Phillips S.A., Zhang D., and Zhang Y. (2011). AVHRR Pathfinder Version 5.2 Level 3 Collated (L3C) Global 4km Sea Surface Temperature for 1981-2011. National Oceanographic Data Center, NOAA. Dataset. doi:10.7289/V5WD3XHB [accessed 02 July 2013]

Casey K.S., Brandon T.B., Cornillon P. & Evans R. (eds.) (2010). *The Past, Present, and Future of the AVHRR Pathfinder SST Program*. Springer, New York.

CBD (2005). Third Report. Convention for Biological Diversity. http://www.cbd.int/reports/default.shtml

CBD (2011). Convention for Biological Diversity. http://www.cbd.int/information/parties.shtml#tab=0.

CIA (2013). *The World Factbook* 2013-14. Washington, DC: Central Intelligence Agency, https://www.cia.gov/library/publications/the-world-factbook/index.html.

CIESIN & CIAT (2005). Center for International Earth Science Information Network (CIESIN)/Columbia University, and Centro Internacional de Agricultura Tropical (CIAT) Gridded Population of the World, Version 3 (GPWv3): Population Density Grid, Future Estimates. Palisades, NY: NASA Socioeconomic Data and Applications Center (SEDAC). http://sedac.ciesin.columbia.edu/data/set/gpw-v3-population-density-future-estimates. Accessed on 2013-08-15.

CITES (2013). http://www.cites.org/eng/disc/parties/chronolo.php.

Claus, S.; De Hauwere, N.; Vanhoorne, B.; Deckers, P.; Souza Dias, F.; Hernandez, F.; Mees, J. (2014). Marine Regions: Towards a global standard for georeferenced marine names and boundaries. *Mar. Geod.*

Costello C., Ovando D., Hilborn R., Gaines S.D., Deschenes O., & Lester S.E. (2012). Status and solutions for the world's unassessed fisheries. *Science*. doi:10.1126/science.1223389.

Dahl T.E. (2006). Status and trends of wetlands in the conterminous United States 1998 to 2004. *Fish and Wildlife Service* 112, 112.

EEA Eionet (European Environment Agency) (2008). http://www.eea.europa.eu/publications

Électricité de France (EDF) (2010). http://energie.edf.com/hydraulique/energies-marines/carte-des-implantations-marines/usine-maremotrice-de-la-rance/publications-51527.html

FAO (2007). The world's mangroves 1980-2005. FAO Forestry Paper 153. Food and Agriculture Organization of the United Nations, Rome, 2007.

FAO Sources (2011).

FAOSTAT (2013). faostat3.fao.org/home/index.html#DOWNLOAD

FAO FishStat (2012). http://www.fao.org/fishery/statistics/software/fishstat/en

FAO FishStatJ (2013). http://www.fao.org/fishery/statistics/software/fishstatj/en

Fetterer, F., Knowles K., Meier W., and Savoie M. (2002, updated 2009). Sea Ice Index. Boulder, Colorado USA: National Snow and Ice Data Center. Digital media.

Frœse R., Branch T., Proelß A., Quaas M., Sainsbury K. & Zimmermann C. (2011). Generic harvest control rules for European fisheries. *Fish and Fisheries* 12(3): 340–351.

Frœse R., Zeller D., Kleisner K. & Pauly D. (2012). What catch data can tell us about the status of global fisheries. *Marine Biology 159*(6): 1283–1292.

Giri C., Ochieng E., Tieszen E., Zhu Z., Singh A., Loveland T., Masek J., Duke N. (2011). Status and distribution of mangrove forests of the world using earth observation satellite data, *Global Ecology and Biogeography* 20(1): 154-159.

Halpern B.S., Longo C., Hardy D., McLeod K.L., Samhouri J.F., Katona S.K., Kleisner K., Lester S.E., O'Leary J., Ranelletti M., Rosenberg A.A., Scarborough C., Selig E.R., Best B.D., Brumbaugh D.R., Chapin F.S., Crowder L.B., Daly K.L., Doney S.C., Elfes C., Fogarty M.J., Gaines S.D., Jacobsen K.I., Karrer L.B., Leslie H.M., Neeley E., Pauly D., Polasky S., Ris B., St Martin K., Stone G.S., Sumaila U.R. & Zeller D. (2012). An index to assess the health and benefits of the global ocean. *Nature* 488: 615-620.

Halpern B.S., Longo C., Scarborough C., Hardy D., Best B.D., Doney S.C., Katona S.K., McLeod K.L., Rosenberg A.A. & Samhouri J.F. (2014). Assessing and improving the health of the California Current with a regional-scale application of the Ocean Health Index. *PLoS ONE* 9: e98995

Halpern B.S., Walbridge S., Selkoe K.A., Kappel C.V., Micheli F., D'Agrosa C., Bruno J.F., Casey K.S., Ebert C., Fox H.E., Fujita R., Heinemann D., Lenihan H.S., Madin E.M.P., Perry M.T., Selig E.R., Spalding M., Steneck R. & Watson R. (2008). A global map of human impact on marine ecosystems. *Science* 319: 948-952.

IFAW (2009). International Fund for Animal Welfare Annual Report.

IUCN (2013). IUCN Red List of Threatened Species. Version 2013.1. http://www.iucnredlist.org. Downloaded on 2013-07-02.

IUCN and UNEP (2013). The World Database on Protected Areas (WDPA). UNEP-WCMC. Cambridge, UK.

Joint Nature Conservation Committee (JNCC) (2004). http://jncc.defra.gov.uk/

Kaschner K., Rius-Barile J., Kesner-Reyes K., Garilao C., Kullander S.O., Rees T. & Frœse R. (2013). AquaMaps: Predicted range maps for aquatic species. World wide web electronic publication,www.aquamaps.org, Version 08/2013.

Kleisner K.M., Longo C., Coll M., Halpern B.S., Hardy D., Katona S.K., Le Manach F., Pauly D., Rosenberg A.A., Samhouri J.F., Scarborough C., Sumaila U.R., Watson R. & Zeller D. in press. Exploring patterns of seafood provision revealed in the global Ocean Health Index. *Ambio*.

Lam, W. H., & Bhushan Roy, C. (2014). Insights into the Ocean Health Index for marine renewable energy. *Renewable and Sustainable Energy Reviews*, *33*, 26-33.

Martell S. & Frœse R. (2012). A simple method for estimating MSY from catch and resilience. *Fish and Fisheries* in press. doi:10.1111/j.1467-2979.2012.00485.x

Ministry for the Environment New Zealand (2007). http://www.mfe.govt.nz/publications Molnar J. L, Gamboa R. L., Revenga C., & Spalding M. D. (2008). Assessing the global threat of invasive species to marine biodiversity. *Frontiers in Ecology and the Environment* 6(9):485–492.

Mora C., Myers R.A., Coll M., Libralato S., Pitcher T.J., Sumaila R.U., Zeller D., Gaston, K.J., & Worm B. (2009) Management effectiveness of the world's marine fisheries. *PLoS Biol* 7(6): e1000131.Musick J. (1999). Criteria to define extinction risk in marine fishes. *Fisheries* 24:, 6-14.NASA (2013). Goddard Earth Sciences Data and Information Services Center. http://disc.sci.gsfc.nasa.gov/data-holdings/PIP/erythemal_uv_irradiance.shtmlNational Bureau of Economic Research (2012). http://www.nber.org/oww/

Nicholls R.J. and Cazenave A. (2010). Sea-level rise and its impact on coastal zones. *Science* 328: 1517-1520.

O'Connor S., Campbell R., Cortez H. & Knowles T. (2009). Whale Watching Worldwide: tourism numbers, expenditures and expanding economic benefits. Special report from the International Fund for Animal Welfare, Yarmouth MA, USA, prepared by Economists at Large.

Ocean Conservancy (2012). Working for clean beaches and clean waters. http://www.oceanconservancy.org/our-work/marine-debris/check-out-our-latest-trash.html

Reynolds R.W., Smith T.M., Liu C., Chelton D.B., Casey K.S. & Schlax M.G. (2007). Daily high-resolution-blended analyses for sea surface temperature. *Journal of Climate* 20: 5473-5496.

Ricard D., Minto C., Jensen O. P. & Baum J. K. (2012). Examining the knowledge base and status of commercially exploited marine species with the RAM Legacy Stock Assessment Database. *Fish and Fisheries 13*(4): 380–398.

Rosenberg A.R., Fogarty M.J., Cooper A.B., Dickey-Collas M., Fulton B., Gutiérrez, N.L., Hyde K.J.W., Kleisner K., Kristiansen T., Longo C., Minte-Vera C., Minto C., Mosqueira I., Osio G.C., Ovando D., Selig E., Thorson J.T. 2014. Developing new approaches to global stock status assessment and maximum sustainable production of the seas. *FAO Fisheries and Aquaculture Report.*

Samhouri J.F., Lester S.E., Selig E.R., Halpern B.S., Fogarty M.J., Longo C. & McLeod K.L. (2012). Sea sick? Setting targets to assess ocean health and ecosystem services. *Ecosphere*.

SAUP (*Sea Around Us Project*) http://www.seaaroundus.org/eez/

Schaefer M. (1954). Some aspects of the dynamics of populations important to the managemetn of commercial marine fisheries. *Bulletin of the International Americal Tropical Tuna Commission* 1: 26-56.

Schutte V.G.W., Selig E.R. & Bruno J.F. (2010). Regional spatio-temporal trends in Caribbean coral reef benthic communities. *Marine Ecology-Progress Series* 402: 115-122.

Schwab K. (2013). The Global Competitiveness Report 2012-2013. World Economic Forum. http://reports.weforum.org/global-competitiveness-report-2012-2013

Selig E.R., Casey K.S. & Bruno J.B. (2010). New insights into global patterns of ocean temperature anomalies: implications for coral reef health and management. *Global Ecology and Biogeography* 19: 397-411.

Short F.T., Polidoro B., Livingstone S.R., Carpenter K.E., Bandeira S., Bujang J.S., Calumpong H.P., Carruthers T.J.B., Coles R.G., Dennison W.C., Erftemeijer P.L.A., Fortes M.D., Freeman A.S., Jagtap T.G., Kamal A.H.M., Kendrick G.A., Kenworthy W.J., La Nafie Y.A., Nasution I.M., Orth R.J., Prathep A., Sanciangco J.C., van Tussenbroek B., Vergara S.G., Waycott M., Zieman J.C. (2011). Extinction risk assessment of the world's seagrass species. *Biological Conservation* 144(7): 1961–1971.

Spalding M.D., Fox H.E., Allen G.R., Davidson N., Ferdaña Z.A., Finlayson M.A.X., Halpern B.S., Jorge M.A., Lombana A.L., Lourie S.A., Martin K.D., Manus M.C., Molnar J., Recchia C.A. & Robertson J. (2007). Marine ecoregions of the world: a bioregionalization of coastal and shelf areas 57(7): 573–583.

Srinivasan U.T., Cheung W.W.L., Watson R. & Sumaila U.R. (2010). Food security implications of global marine catch losses due to overfishing. *Journal of Bioeconomics 12*(3): 183–200.

Thorson J.T., Minto C., Minte-Vera C.V, Kleisner K. & Longo C. (2013). A new role for effort dynamics in the theory of harvested populations and data-poor stock assessment. *Canadian Journal of Fisheries and Aquatic Science*, in press. doi:10.1139/cjfas-2013-0280

Trujillo P. (2008). *in*: The Performance of 53 Countries in Managing Marine Resources, edited by J. Alder and D. Pauly (Fisheries Centre Research Reports Fisheries Centre, University of British Columbia, Vancouver, Canada, 2008).

UN Energy Statistics Database (2012). http://data.un.org/Data.aspx?d=EDATA&f=cmID:EO;trID:01&c=2,5,6,7,8&s=_crEngNameOrderBy:asc,_enID:asc,yr:desc&v=1

US Energy Information Administration (2011). http://www.eia.gov/countries/prices/electricity_industry.cfm

UNDP, Human Development Report (2010).

Schwab K. (2012). The Global Competitiveness Report 2012–2013: Full Data Edition. World Economic Forum, Geneva, 2012.

Visconti P., Di Marco M., Álvarez-Romero J.G., Januchowski-Hartley S.R., Pressey R.L., Weeks R. & Rondinini C. (2013). Effects of errors and gaps in spatial data sets on assessment of conservation progress. *Conservation Biology* 27(5): 1000-1010.

VLIZ (2012). Maritime Boundaries Geodatabase, version 7. Available online at http://www.marineregions.org/. Accessed on 2013-07-19.

Watson R., Kitchingman A., Gelchu A. & Pauly D. (2004). Mapping global fisheries: sharpening our focus. *Fish and Fisheries* 5(2): 168–177.

WEF (World Economic Forum) 2013. www.weforum.org/issues/travel-and-tourism-competitiveness

WHO-UNICEF Joint Monitoring Programme (2013). http://www.wssinfo.org/

Worldwide Governance Indicators (last updated: 14-Sep-2012): http://www.govindicators.org.

World Tourism Travel Council (2013). http://www.wttc.org/research/economic-data-search-tool/

WTTC World Travel and Tourism Council. 2013. www.wttc.org/research/economicimpact-research/methodology/

WWF (2013). Priority and endangered species.
http://wwf.panda.org/what_we_do/endangered_species/
Zeileis A. & Grothendieck G. (2005). zoo:S3 Infrastructure for regular and irregular time series. *Journal of Statistical Software* 14: 1-27.
